# Using narratives to impact health policy-making: a systematic review

**DOI:** 10.1186/s12961-019-0423-4

**Published:** 2019-03-05

**Authors:** Racha Fadlallah, Fadi El-Jardali, Mohamed Nomier, Nour Hemadi, Khurram Arif, Etienne V. Langlois, Elie A. Akl

**Affiliations:** 1 Center for Systematic Review for Health Policy and Systems Research, American University of Beirut, Beirut, Lebanon; 2 Department of Health Management and Policy, Faculty of Health Sciences, American University of Beirut, Beirut, Lebanon; 3 Alliance for Health Policy and Systems Research, World Health Organization, Geneva, Switzerland; 4 Department of Internal Medicine, American University of Beirut, Beirut, Lebanon

**Keywords:** Narrative, storytelling, health policy-making, systematic review

## Abstract

**Background:**

There is increased interest in using narratives or storytelling to influence health policies. We aimed to systematically review the evidence on the use of narratives to impact the health policy-making process.

**Methods:**

Eligible study designs included randomised studies, non-randomised studies, process evaluation studies, economic studies, qualitative studies, stakeholder analyses, policy analyses, and case studies. The MEDLINE, PsycINFO, Cochrane Library, Cumulative Index to Nursing and Allied Health Literature (CINAHL), WHO Global Health Library, Communication and Mass Media Complete, and Google Scholar databases were searched. We followed standard systematic review methodology for study selection, data abstraction and risk of bias assessment. We synthesised the findings narratively and presented the results stratified according to the following stages of the policy cycle: (1) agenda-setting, (2) policy formulation, (3) policy adoption, (4) policy implementation and (5) policy evaluation. Additionally, we presented the knowledge gaps relevant to using narrative to impact health policy-making.

**Results:**

Eighteen studies met the eligibility criteria, and included case studies (*n* = 15), participatory action research (*n* = 1), documentary analysis (*n* = 1) and biographical method (*n* = 1). The majority were of very low methodological quality. In addition, none of the studies formally evaluated the effectiveness of the narrative-based interventions. Findings suggest that narratives may have a positive influence when used as inspiration and empowerment tools to stimulate policy inquiries, as educational and awareness tools to initiate policy discussions and gain public support, and as advocacy and lobbying tools to formulate, adopt or implement policy. There is also evidence of undesirable effects of using narratives. In one case study, narrative use led to widespread insurance reimbursement of a therapy for breast cancer that was later proven to be ineffective. Another case study described how the use of narrative inappropriately exaggerated the perceived risk of a procedure, which led to limiting its use and preventing a large number of patients from its benefits. A third case study described how optimistic ‘cure’ or ‘hope’ stories of children with cancer were selectively used to raise money for cancer research that ignored the negative realities. The majority of included studies did not provide information on the definition or content of narratives, the theoretical framework underlying the narrative intervention or the possible predictors of the success of narrative interventions.

**Conclusion:**

The existing evidence base precludes any robust inferences about the impact of narrative interventions on health policy-making. We discuss the implications of the findings for research and policy.

**Trial registration:**

The review protocol is registered in PROSPERO International prospective register of systematic reviews (ID = CRD42018085011).

**Electronic supplementary material:**

The online version of this article (10.1186/s12961-019-0423-4) contains supplementary material, which is available to authorized users.

## Background

Narratives, also referred to as storytelling, exemplars and testimonials, can be used to convey information because they are easily understandable and memorable [1, 2]. While there is no commonly accepted definition of a narrative, it is generally agreed that it should provide an account of an individual’s experience and include appealing detail, characters and some plot [3–7]. It may be communicated through a wide range of formats, including verbal (narrated), print, audio and video [8, 9].

Narratives can facilitate information processing and provide value and emotional appeal to the information provided [8, 10]. Additionally, people can relate to narrative information regardless of their level of literacy, expertise or culture [11, 12]. These narratives have been demonstrated to be both memorable and persuasive [13, 14]. Several theoretical perspectives have been considered to explain the persuasiveness of narrative information [8, 15–20]; see Table 1 for a description of these models.

**Table 1:** Description of models relevant to narratives

Model	Description
Elaboration likelihood model (ELM)	Under this widely adopted ELM, persuasive situations involve a central and a peripheral route [15]. In the central or systematic route, people think deeply about the message and assess the validity of the information. In the peripheral or heuristic route, people assess characteristics of the message, such as its attractiveness; the personal relevance of the message determines the route that will be used for its perception [15]
Green’s transportation-imagery model	This model states that the persuasiveness of a narrative depends on the extent to which people are engaged in the story events, a phenomenon called “*transportation*” [17]. In this model, recipients are totally immersed in the story and engage emotionally with the story characters and events. Therefore, they are less likely to counter-argue the narrative claims; this contrasts with the ELM, where recipients elaborate on the story with their thoughts, ideas and previous experiences [16]
Entertainment overcoming resistance model	According to this model, when recipients are engaged within the story (Transportation), or when they relate to characters similar to themselves (Identification), they are less likely to be resistant to receive the message and change their attitude and behaviour [18, 20]. According to this theory, several narrative characteristics could determine how much the recipients can accept the message such as the level of suspense and imagery in the narrative and the degree of realism that recipients can associate with the story [19]
Kahneman’s two-system way of thinking	Kahneman distinguishes between two systems of thinking: ‘System 1’ (Thinking Fast) is the intuitive way of thinking and making decisions (i.e. relying on heuristic or cognitive shortcuts that develop as part of people’s experiences), while ‘System 2’ (Thinking Slow) is the analytical and deliberate way of making decisions (i.e. weighing the advantages and disadvantages) [7]. Systems 1 and 2 are differentially activated by different aspects of narratives

The communication literature offers some insights on the effects of different ways of communicating information on behaviour. First, individuals make choices based on incorporating both factual and narrative information; narrative information influences individuals’ choices directly (system 1) and indirectly via cognitions (system 2), and the persuasiveness of narrative or statistical information varies depending on the characteristics and experiences of the recipients [21, 22]. These insights imply that it is naive to assume that it is sufficient to present people with ‘facts’, and expect that they will weigh these in a rational manner and act accordingly [23].

There is growing recognition among experts in the field of public policy-making of the need to incorporate narrative as an important component of the broad evidence base required to inform complex policy-making processes [23, 24]. This is particularly so given that policy decisions are often value-driven and political, not just evidence-based choices [25], and that policy-makers and public health professionals operate on a different hierarchy of evidence compared to researchers [26]. For instance, policy-makers prefer information that is concise, appealing and relevant to current health policy debates [27–29].

As highlighted by Cairney and Kwiatkowski [30], policy-makers attach cognitive and emotional shortcuts to thoughts and action, often without fully understanding the reasons for that action; therefore, ‘bombarding’ them with evidence can be less effective than presenting them with compelling stories or using other framing techniques to harness their cognitive biases. In this sense, narrative information can be more useful than statistical data, partly because the latter can be seen as too complex, not policy-relevant, tedious or lacking context sensitivity [31, 32].

The use of narratives in the policy environment can help identify important policy issues, point to problems with existing policies, provide evidence that a programme or law is working as intended, and assist policy-makers in thinking about the consequences of policy options [10, 27, 31, 33]. For instance, personal stories of breast cancer have been key in creating significant changes in health policies and legislative allocations in the United States [33]. While there is a growing number of reports on the use of narrative-based interventions to shape policy-making, we are not aware of any systematic synthesis of that body of evidence. The objective of this study was to systematically synthesise the evidence on the use of narratives to impact health policy-making.

## Methods

### Study design and definitions

We conducted a systematic review of the literature, following standard methodology. We registered the review protocol in the PROSPERO International prospective register of systematic reviews (ID = CRD42018085011) and we followed the PRISMA guidelines for reporting systematic reviews.

For the purpose of this study, we conceptualised a narrative as an illustration of an experience in a story-like format, presented in either the first or the third person [2]. The terms that we considered as referring to ‘narratives’ include storytelling, anecdotes, exemplars, testimonials and policy narratives [7, 21]. Given that the goal of this review was to inform those interested in using narrative information to affect health policy-making, we restricted our eligibility to studies where the primary purpose of using a narrative was to affect policy-making (i.e. narrative as a planned intervention). The narrative could be presented in any format (e.g. verbal, print, audio/radio, video) or perspective (first- or third-person narrative).

Public policy refers to government policy and includes programmes, plans, rules, legislation, guidelines, statements or positions taken by government or governmental departments with the aim of achieving population-level change (whether at the sub-national, national or international level) [34]. This excludes policies confined to one institution only or those related to individual-level clinical interventions [35]. We only considered public policies pertaining to health.

### Eligibility criteria

We used the following eligibility criteria:

Type of studies: We included a range of types of studies to account for the diverse literature on narratives and the complex nature of evidence in the policy sector [36]. Specifically, we included randomised studies, non-randomised studies (e.g. cohort studies, before and after studies, retrospective studies, and cross-sectional studies), process evaluation studies, economic studies, qualitative studies, stakeholder analyses, policy analyses and case studies. We excluded news articles, books, letters, commentaries, opinion pieces, proposals, reviews and studies published in abstract format only. We also excluded studies where narrative was mentioned as part of the background information only.

We did not exclude studies based on date of publication or language.

- Population: We included studies where the narrative intervention targeted legislators, policy-makers, representative of professional associations, governmental representatives or any other individuals involved in health policy-making. We excluded studies where the narrative intervention targeted patients or people in their individual capacity (e.g. in a clinical setting).
- Interventions: Narratives used as standalone or as part of a multi-component intervention with the primary purpose of influencing health policy-making in a real-world setting. We excluded studies where the narrative was not an explicit or deliberate component of the intervention. We also excluded studies that assessed message-framing only or that used narrative for information delivery without any link to the policy cycle.
- Comparison: We included studies regardless of whether or not they have a comparison group.
- Outcomes: We included studies that examined the influence of narrative information on any of the stages of health policy-making in a real-world setting [37]. We stratified findings according to the stages of policy-making, as defined by the Stages Heuristic framework, as follows: (1) agenda-setting, (2) policy formulation, (3) policy adoption, (4) policy implementation and (5) policy evaluation (see Table 2 for a detailed definition of the different stages) [37–40]. We excluded studies that assessed proxy outcomes such as changes in knowledge, beliefs, attitude, preferences or intentions. We also excluded studies that involved individuals making hypothetical decisions. Additionally, we excluded studies that assessed the impact of narrative on public opinion only or that examined policy-making processes beyond the health or health-related sector.
- Settings: We included any country, state or community

**Table 2:** Description of stages heuristic framework

Stage	Description
Agenda-setting	Process through which an issue or problem reaches the policy agenda and gets the attention of policy-makers; this usually occurs when an interest group demands government action on a problem, or when there is public disagreement over ways in which a problem should be addressed
Policy formation	A set of policy alternatives and solutions is generated and the public administration concerned examines the various policy options. Coalitions of actors strive, through the use of advocacy strategies, to gain priority for one specific option.
Policy adoption	Decisions are made at the governmental level, resulting in a decision that favours one or more options to addressing a given problem. Decisions are given a legal force or legitimised as a result of the public statements or actions of government officials; this includes executive orders, laws and appropriations, rules and regulations, and administrative and court decisions that set policy directions
Policy implementation	This includes the actions and mechanisms whereby adopted policies are brought into practice; social, economic, technological, and political conditions significantly influence the implementation stage of public policies
Policy evaluation	This assesses whether policies have achieved their intended goals and objectives; it covers the appraisal of their content, their implementation and their effects

### Search strategy

We searched the following electronic databases up to February 2017: MEDLINE, PsycINFO, Cochrane Library, Cumulative Index to Nursing and Allied Health Literature (CINAHL), WHO Global Health Library, and Communication and Mass Media Complete. The search strategy combined the two different concepts of ‘narrative’ and ‘public policy’. To generate a list of search terms for each concept, we first undertook an initial targeted search of the literature, followed by an analysis of the text words contained in the title and abstract of potentially relevant studies as well as of the index terms used to describe the article. Additionally, we reviewed the search strategies of relevant systematic reviews. This helped generate an initial list of terms relevant to each of the two concepts of ‘narrative’ and ‘public policy’, respectively including, for example, narrative, narration, testimonial, anecdote, exemplar, and story, and policy, public policy, health policy, reform, lobbying, regulation, law enforcement, policy-making, government, law, legislation, decree, jurisprudence, advocacy, decision-making, etc.

The search included both free-text words and medical subject headings. We used the Boolean operator ‘OR’ to combine the terms within each concept and the Boolean operator ‘AND’ to combine the different concepts. We did not use any search filter for study type, language or date of publication. The search strategy was validated by an experienced medical librarian (Additional file 1).

We complemented the electronic database searches with a variety of approaches to identify additional literature, including grey literature. We manually searched Google Scholar and relevant journals like *Health Affairs*. We also screened the reference lists of included studies and relevant systematic reviews. In addition, we contacted the authors of relevant articles and conference proceedings for further information or additional material.

### Study selection

We conducted the selection process in two phases, namely title and abstract screening, wherein teams of two reviewers screened the titles and abstracts of identified citations, in duplicate and independently, for potential eligibility and retrieved the full text of studies judged as potentially eligible by at least one of the two reviewers, and full-text screening, wherein two reviewers screened the full texts in duplicate and independently for eligibility, using a standardised and pilot-tested screening form, and resolving disagreements by discussion or with the help of a third reviewer.

Prior to the selection process, all the reviewers participated in a calibration exercise using a randomly selected sample of 100 citations. The calibration exercise allowed us to pilot the eligibility criteria to ensure they are applied in the same way across reviewers, thus enhancing the validity of the process.

### Data abstraction

Two reviewers abstracted data from eligible studies in duplicate and independently using standardised and pilot-tested data abstraction forms. They conducted a calibration exercise on a randomly chosen sample to ensure adequate agreement and resolved disagreement by discussion or with the help of a third reviewer if consensus could not be reached.

Abstracted data focused on the variables of study design, timeframe and background story; health topic, organiser, target population and setting; narrative definition and theoretical framework used; format of narrative (verbal, print, audio, video) and characteristics of the narrative information (plot and characters); characteristics of the intervention used to deliver the narrative; and policy outcomes assessed.

### Quality assessment

We planned to assess risk of bias and quality of reporting of the included studies, using tools appropriate for the study design. However, we did not assess the risk of bias of included studies given that existing tools to critically appraise the types of studies retrieved have not been validated, and thus have strong limitations at this stage (as well as lack of consensus). We did assess the quality of reporting of case studies according to the reporting standards developed by Rodgers et al. [41]. These standards consist of 13 items grouped into four sections, as (1) describing the design (4 standards); (2) describing the data collection (3 standards); (3) describing the data analysis (1 standard); and (4) interpreting the results (5 standards).

### Data synthesis

We conducted thematic analysis and presented the results in a narrative way, stratified according to the following stages of the policy cycle: (1) agenda-setting, (2) policy formulation, (3) policy adoption, (4) policy implementation and (5) policy evaluation [37]. Two reviewers were involved in the identification of the themes. Where applicable, we provided direct quotations. Additionally, we assessed the knowledge gaps related to our topic. We assessed five domains most commonly used in published evidence gap maps, i.e. study design, interventions, setting, population and outcomes [42].

## Results

### Study selection

**Fig. 1:**
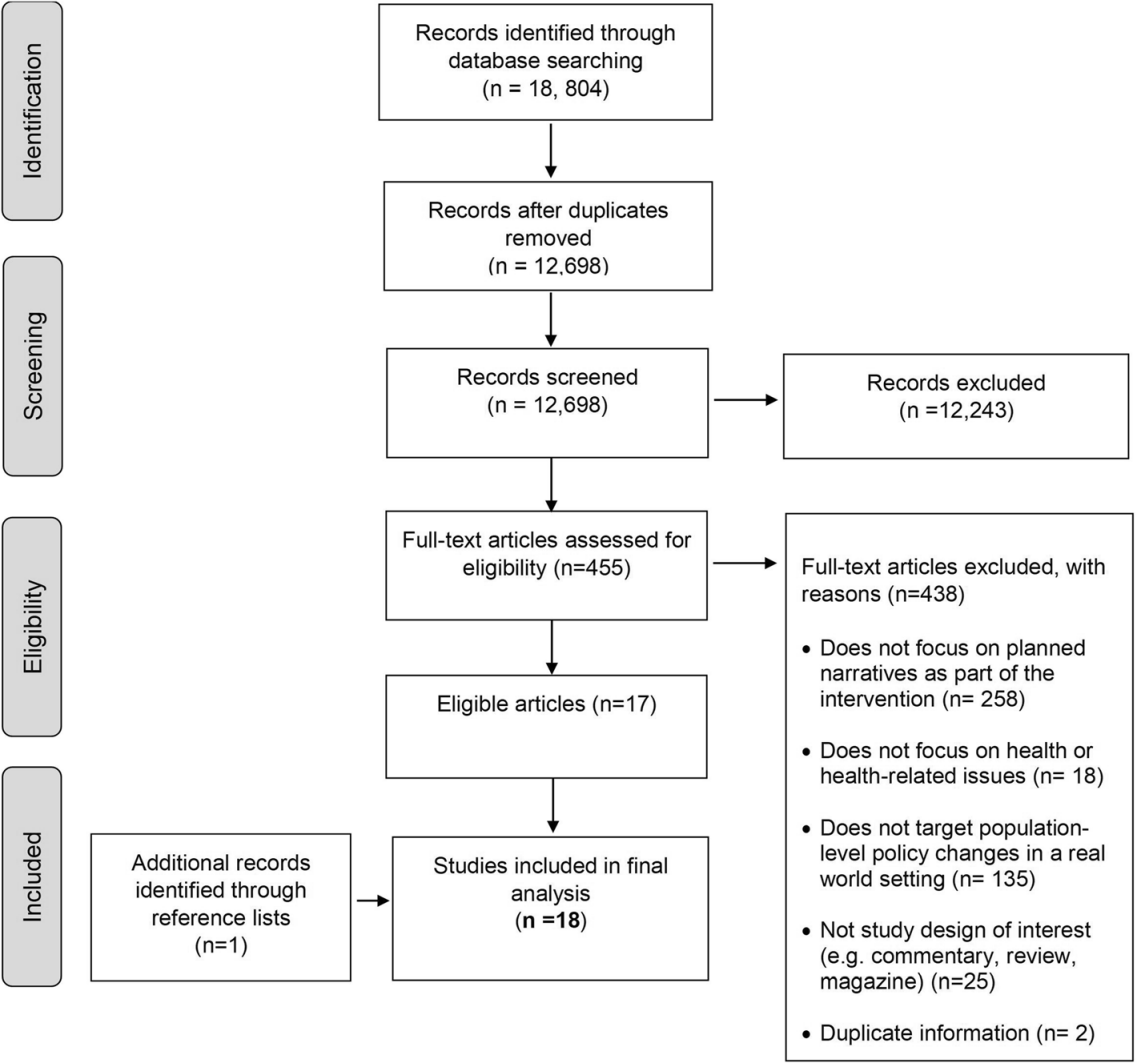
Study flowchart

Figure 1 shows the PRISMA flow chart summarising the study selection process. Out of 12,698 citations, we identified 18 eligible studies. We excluded 438 articles at the full-text screening phase because they did not focus on planned narratives as part of the intervention (*n* = 258), did not focus on health or health-related issues (*n* = 18), did not target population-level policy changes in a real world setting (*n* = 135), were not a study design of interest (e.g. commentary, review, magazine) (*n* = 25), or contained duplicate information (*n* = 2) (Additional file 2).

### Characteristics of studies

Table 3 describes the characteristics of the 18 included studies in terms of study design, timeframe, background story, health topic, target population, organisers of narrative-based interventions, country, narrative definition and theoretical framework used, format of narrative (verbal, print, audio, video), characteristics of the narrative information (plot and characters), characteristics of the intervention used to deliver the narrative, and policy outcomes assessed.

**Table 3:** Characteristics of included studies

Study name Study design Time frame Background story	Health topic, Target population, Organiser, Country	Definition of narrative and theoretical framework adopted	Format (verbal, print, audio, video) and characteristics of narratives (plot and characters)	Characteristics of the intervention used to deliver the narrative	Policy outcome assessed (agenda-setting, policy formulation, policy adoption, policy implementation, policy evaluation)
Beck et al., 2014 [58] Documentary analysis Timeframe not reported Data of about 157 celebrities, including athletes, actors, musicians, and politicians, who disclosed private information regarding a personal health situation (or that of a loved one) with others in the public domain were collected by authors	Health topic: Multiple sclerosis, cancer, Parkinson’s disease, Alzheimer’s disease, HIV, Hugh Downs, bipolar II disorder, eating disorder, diabetes, paralysis, cystic fibrosis, amyotrophic lateral sclerosis Target population: Congress, national research organisations, professional associations, and general public Organiser: Celebrities or their family members Country: United States of America	Narrative boundaries, informed by the theory of Communication Privacy Management “*Construction of narrative may be understood from performative and dialogical perspectives as always a joint enterprise with an active audience*”	Format: Brief comments in the tabloids, interview on television or for a magazine about a condition, and verbal (narrated) Characteristics of narratives: Not reported	Some celebrities have established foundations which focus on raising money and/or awareness while others have testified before Congress on behalf of themselves or their loved ones for specific diseases	Agenda-setting: Key functions that celebrity health narratives performed included education, inspiration and activism (e.g. after hearing the story of Michael Zaslow, one senator committed to doubling funding for research at the National Institutes of Health so that they can treat and cure diseases such as amyotrophic lateral sclerosis). Although celebrities bring much attention to a cause on behalf of individuals suffering from it, the authors questioned whether such advocacy should frame federal priorities in terms of funding
Fitzgerald, 2013 [44] Case study In 2006, 2010 and 2011, attempts were made by health policy advocates to ignite policy debate about the introduction of supervised injecting facilities in Australia; despite numerous attempts, only one facility has been introduced in Sydney, thus this article retraces the policy narratives to reflect on the causes for policy impermeability	Health topic: Supervised injecting facilities (SIF) for drug users Target population: Government Organiser: Author and other advocates for implementation of policies supporting supervised injecting facilities Country: Australia	Narrative Policy Framework approach “*A policy narrative is the story about a policy idea embedded in documentation about the idea. Like any story, the narrative describes the idea, frames the policy and connects the idea with other ideas and ultimately directs those in the policy arena to solutions to problems*”	Format: Photographs and videos portraying drug users Characteristics of narratives: In Sydney, villains of police corruption and police cynicism were prominent in the Wood Royal Commission that framed SIF policy development, whereas in the Melbourne context, the policy narratives focused on health and police were cast alongside the drug outreach workers as heroes who would clean up the streets with the real villains being drug users	In Sydney and Melbourne, two front-page newspaper photographs reoriented the SIF policy narrative, and the policy narratives took different directions: In Sydney, the *Sydney Sun Herald* photograph of a teenage boy injecting in the backstreets of Redfern was the catalyst for commitment to the 1999 drug summit and mobilising support for a SIF, especially since the police could not be trusted to provide order. In Melbourne, the *Melbourne Herald Sun* page 1 photograph captioned “*How can we stop this?*” reinforced a zero-tolerance policing response with quest for order reinforcing the authority of the police, thus undermining the legitimacy of the SIF policy Pictures were complemented by trials, reports, royal commissions and advocacy	Policy adoption: In Sydney, 22 months after the Drug Summit Legislative Response Act 1999 was passed for the trial of the supervised injecting facilities, the service opened in May 2001. In Victoria, the bill failed to pass through both Houses of the Victorian Parliament
Johnson et al., 2014 [43] Case study Time frame not reported Concerns about barriers facing individuals with intellectual disabilities in expressing their sexuality or in having sexual relationships, prompted individuals with intellectual disabilities to conduct research where they tell their life stories and talk about sexuality and relationships, with findings subsequently used to promote change	Health topic: Sexual lives and relationships of people with intellectual disabilities Target population: Government and general public Organiser: Individuals with intellectual disabilities and their advocates Country: Australia and the Republic of Ireland	Not reported	Format: Short films, theatres and plays, and formal presentations Australia: Three short films of the stories of people with intellectual disabilities Ireland: Stories of people with intellectual disabilities provided the basis for a creative analysis in the form of theatre and plays Characteristics of narratives: not reported	Australia: Short films of the stories of people with intellectual disabilities were used by organisations that had been part of the advisory group to lobby government for a change to the existing policy in relation to people with disability and sexuality Ireland: Use of theatre and plays brought the group to the notice of the media. Research, drama and media appearances brought the issue of the prohibition under Irish law of penetrative sexual activity with a person with intellectual disabilities to public attention and two representatives from the research group were invited to consultations with government legislative advisory body with regard to reviewing the 1993 Act in March 2011	Australia – Policy formulation: Drafting of a new policy for state government in relation to sexuality and relationships and people with disabilities which state that people with disabilities had rights to relationships and to a sexual life, and set out rights and responsibilities for people with intellectual disabilities and service providers about relationships and sexuality Ireland – Agenda-setting: The 1993 Act in the Republic has not yet been changed, but there is now a significant voice from people with intellectual disabilities informing discussion of how this might happen
Krueger, 2007 [51] Case study Throughout mid-to-late twentieth century, cancer-related organisations began integrating the voices, images and stories of young sufferers into their annual campaigns for the disease, although children represented only a small fraction of all persons with cancer	Health topic: Early cancer detection Target population: Donors, philanthropists, public and politicians Organiser: Publicists and cancer campaigners in the mid-twentieth century Country: United States of America	Not reported	Format: Poster children with all-American attributes and interests (i.e. appealing to regional donors, personified cancer and served as reminder that cancer did not spare children) Characteristics of narratives: “*Heroic accounts and images of children illustrating possibility of remission and tumor regression continued to be selective in nature, carefully crafting optimistic ‘cure’ or ‘hope’ stories to raise money for cancer research that ignored negative realities*”	Staged photographs of children with cancer standing side by side with famous personalities, vividly demonstrating the efficacy of new cancer therapies at annual fund-raising conventions, or celebrating holidays in hospital wards elevated viewers’ emotional tie to the cancer. Sentimental images paired with frightening captions and text sent parents a stern warning that any child, every child or even their child was vulnerable to the threat of this disease	Agenda-setting: More attention to children cancers: “*Poster children were strategically used throughout the mid-to-late twentieth century to advance principles of early cancer detection and prompt treatment; to illustrate or, at times, exaggerate promising biomedical advances in the field; and to elicit emotional responses and donations from a wide audience during the escalation of the war against cancer*”
Lander, 2007 [59] Biographical method Mifepristone, which could halt the growth of certain tumours, is unapproved in Australia because it interferes with progesterone levels (i.e. an abortifacient); in 2005, the author triggered the campaign to amend the legal status of the drug when she was diagnosed with meningioma	Health topic: Approval of the drug mifepristone for use as a treatment for meningioma Target population: Legislators Organiser: Mary Lander (article author) who was diagnosed with meningioma Country: Australia	Not reported	Format: Verbal (face-to-face encounters) and broadcasts Characteristics of narratives: Not reported	Mary Lander contacted the Senator leading the Australian Democrats. The Senator found out that the drug could be obtained through the Special Access Scheme of the Therapeutic Goods Administration. On 7 September 2005, the Senator gave a speech in Parliament entitled “Matters of public importance – mifepristone”, launching the campaign to amend the legal status of the drug. Mary Lander participated in some broadcasts	Policy adoption: Parliament successfully voted to repeal ministerial responsibility for approval of the drug mifepristone in early 2006
Leith and Phillips, 2006 [46] Case study In 1994, the Optional Coverage for Women and Infants programme under Medicaid was greatly endangered due to proposed legislative budget cuts. Advocacy summaries were used to demonstrate to legislators the need for continuing the programme	Health topic: Optional Coverage for Women and Infants programme under Medicaid Target population: Legislators Organiser: A state agency in a south-eastern state Country: United States of America	Not reported	Format: Advocacy summaries that gave a qualitative description of personal experiences of callers with the programme Characteristics of narratives: Not reported	State agency ran a Medicaid Advocacy Hotline, which handed calls from pregnant women, mothers of young children and other family members from across the state. Information collected from the hotline was used to create thousands of advocacy summaries consisting of brief narratives of 3 to 8 sentences, compiled by county and by month/year. Advocacy summaries were shared on a monthly basis with a state-wide interagency maternal and child health ‘Access Task Force’	Policy adoption: Optional Coverage for Women and Infants programme was “*spared from proposed legislative budget cuts*”
Lorenzo, 2008 [57] Qualitative research approach using participatory action research (PAR) Time frame not reported People from impoverished communities struggled to gain daily access to economic and social resources because of long distances, inadequate public transport facilities as well as overall poor levels of service	Health topic: Disabled women mobilising for an accessible public transport system Target population: Government and disabled women Organiser: Disabled People South Africa’s Disabled Women’s Programme in the Western Cape, the South African Christian Leadership Assembly Health Project, and Department of Occupational Therapy at Cape Town Country: South Africa	Interpretive Critical Theory Paradigm	Format: A series of narrative action reflection workshops as an innovative method that enables storytelling and action learning collectively rather than on a one-to-one basis Characteristics of narratives: Not reported	Storytelling and action learning between workshops were captured through videotapes, audiotapes, scribing, field notes and photographs. Findings were disseminated through seminars, conferences, narrative action reflection workshop, documentations, and writing up research	Agenda-setting: “*Findings contributed to increased understanding and awareness of disability issues for individuals involved in policy development and implementation across the various sectors of government to improve service delivery mobilising for an accessible public transport system*”
MacGregor and Mills, 2011 [52] Case study In 2003, as part of a campaign to secure treatment for HIV drugs for all, a group of women involved with the Treatment Action Campaign and Medicines Sans Frontières participated in an initiative to ‘map’ their bodies as affected by HIV	Health topic: Right to HIV treatment Target population: Government Organiser: Medicines Sans Frontières Country: South Africa	Not reported	Format: Body mapping (an innovative narrative and art therapy) through which women tell how their lives have been transformed by antiretroviral treatment Characteristics of narratives: Not reported	A book, ‘Long Life: Positive HIV Stories’, containing the women’s body maps and narratives was published and used as a political tool to add the ‘voices’ of ordinary people to the campaign to lobby for treatment for all	Policy implementation: Roll out of universal access to antiretroviral treatment drug in the public sector
MacKenzie et al., 2008 [54] Case study using frame analysis In 2007, Clare Oliver was dying from melanoma at the age of 26; in the last months of her life, she campaigned strongly against the use of tanning beds and called for a ban	Health topic: Solarium tanning Target population: Government, media and the general public Organiser: Clare Oliver Country: Australia	Not reported	Format: First person accounts, television and print media coverage of the campaign Characteristics of narratives: Not reported	The policy changes were thought to result from the combination of powerful personal stories of people whose melanoma was associated with use of tanning beds, media advocacy, political representation and lobbying, and epidemiological evidence	Policy adoption: regulation of the solarium industry (e.g. banning access to tanning beds for those younger than 18 years)
Marcus, 2010 [53] Case study A cancer survivor who became a patient advocate and navigator shared his experiences with TV writers; the storyline, entitled “You Don’t Cut into Cancer”, first aired on April 21, 2005	Health topic: Patient navigators for chronic diseases Target population: Congress Organiser: Representatives of the National Cancer Institute at the National Institutes of Health and writers from the NBC medical drama *ER*, as well as Hollywood, Health and Society advisory board Country: United States of America	Not reported	Format: Primetime network TV storyline addressing cancer patient navigators Characteristics of narratives: Storyline featured an elderly African-American woman who avoided surgery for a lump in her breast because she believed that cutting into cancer would cause it to spread. A physician arranges for a visit with an African-American breast cancer survivor who is also a patient navigator. The survivor shares her experience with Mrs Graham and offers to help. As the episode ends, Mrs Graham appears willing to receive treatment	Clips of the episode were shown to raise awareness of patient navigators in a Congressional Committee meeting. Congressional staffers report that the episode provided a tangible example of how patient navigator programmes would work	Policy adoption: The storyline contributed to critical policy discussion that led to passage of United States law HR 1812, the Patient Navigator Outreach and Chronic Disease Prevention Act of 2005
Neuhausen, 2013 [48] Case study Grady, the fifth-largest public hospital in and the largest provider of indigent care in Georgia, was caught in a ‘perfect storm’ of economic troubles. Medical students led a year-long campaign in 2007 to fight for a vital safety-net hospital in Georgia	Health topic: Safety-net hospital ‘Grady’ for poor and uninsured residents Target population: Legislators and governors Organiser: Students and resident physicians under the lead of ‘Health Students Taking Action Together (HealthSTAT)’ Country: Georgia	Not reported	Format: Scripted narrative (stories from Grady patients were collected to create patient story cards with photos of patients on the front and quotes on the back) Characteristics of narratives: In one card, the front included a middle-aged African American man looking back with tired eyes. The back of his card read: “*When my health was failing, I lost my job and was left without insurance. I turned to Grady when my heart failure worsened to the point where I had to be hospitalised. Since then, I have gotten back on track, working with my doctors and nurses at Grady to make sure that I keep myself healthy and out of the hospital. I want to give what I can to help this hospital because I know there are many more people out there just like me who cannot afford to have Grady close*”	Stories collected from Grady patients of the life-saving care provided by Grady, were described as the most powerful advocacy tool. Patient stories were complemented by a range of activities, including State-wide campaign to get state legislature to increase state funding; writing to governor, lieutenant governor, or speaker of the House, asking them to increase state support to Grady; talking one-on-one with the legislators; inviting legislators to visit Grady to gain first-hand experience; and testifying on day of Commissioners’ vote on Grady’s central role in caring for the working poor	Policy adoption: Commissioners voted to approve the non-profit conversion which was critical to Grady’s survival. Georgia General Assembly approved increases in Medicaid reimbursement rates to trauma hospitals and authorised US$58 million to support uncompensated care at state trauma centres
Rosenbaum, 2016 [56] Case study In 2013, Amy Reed, underwent a hysterectomy with intraoperative morcellation for presumptively benign uterine fibroids. The tumour turned out to contain leiomyosarcoma (LMS), a rare aggressive cancer, and the procedure resulted in dissemination of the cancer	Health topic: Use of morcellators in gynaecologic surgeries Target population: Legislators, government, medical profession, public Organiser: Amy Reed’s husband Hooman Noorchashm, then a cardiac surgery fellow Country: United States of America	Not reported	Format: Verbal and print Characteristics of narratives: Availability bias exaggerated the risk of LMS with media coverage featuring the faces of women dying of LMS, ravaged by chemotherapy, flanked in photos by their husbands and young children. Meanwhile, the benefits of morcellation are largely invisible and thus “*unavailable*”	Reed’s husband launched a campaign to ban morcellators. The *Wall Street Journal* picked up the story in December 2013. Subsequently, the Food and Drug Administration undertook a review to quantify the risk of disseminating occult uterine cancers that cannot be reliably detected preoperatively. The resultant safety communication, was issued in April 2014	Policy implementation: FDA issued a black-box warning stating that morcellation was contraindicated in perimenopausal or postmenopausal women and in “*candidates for en bloc tissue removal*”. Many institutions banned morcellation and some insurers stopped covering the procedure or began requiring prior authorisation. Author argues that there may be greater population benefits and lesser risks from continuing than from discontinuing morcellator use, thus claiming anecdote can skew risk perception
Sharf, 2001 [33] Case study In 1975, Kushner, a journalist and cancer survivor, put her investigative skills to use in understanding the cancer that afflicted her. In 1993, Nelene Fox, a 38-year-old mother was diagnosed with advanced breast cancer and was advised that her only remaining chance for survival was an autologous bone marrow transplant (ABMT), which her insurance refused to pay for ($US 140,000) because of insufficient scientific evidence to prove its efficacy; Senator Tom Harkin’s interest in breast cancer was influenced by the death of his only two sisters at a young age from breast cancer, without ever doing a mammogram	Health topic: Breast cancer Target population: Cancer establishments, legislators, Congress, public Organiser: Patients with breast cancer and advocates Country: United States of America	“*Personal narratives are powerful, rhetorical strategies, as well as humane expressions of suffering and memorials to loved ones. The riveting communication of such narratives enlightens our understanding of what it means to live with breast cancer*”	Format: Personal narratives of patients living with breast cancer elicited through conversations, popular books, newspapers and magazines, and television Characteristics of is: Not reported	Kushner wrote a book, entitled ‘Breast Cancer: A Personal History and an Investigative Report’, which provided a brief account of her own illness, with a lengthy analysis and critique of the then current epidemiological and clinical approaches to breast cancer. Her book was excerpted in newspapers and women’s magazines, and remained in circulation until the early 1990s. Fox’s brother, a lawyer, sued the insurance and convinced the jury to award $89,000 in damages to her family. Similar lawsuits with similar results soon followed, further fuelled by media publicity. Questions that many physicians had about the efficacy of ABMT were compounded by prolonged difficulty in recruiting enough subjects for controlled clinical trials. Senator Harkin has championed funding of medical research for breast cancer over the past decade, characterising it as “*a leading killer*”	Policy implementation: Kushner’s efforts resulted in a change of standard clinical procedure to the two-step biopsy and treatment decision. Fox’s story succeeded in forcing widespread insurance reimbursement for ABMT even though there was little or no data to support this choice, further discouraging patients from enrolling in clinical trials. Thus, conclusions about the lack of efficacy of the treatment for patients with breast cancer were delayed until 1999. Senator Harkin’s legislative achievements included dramatically increasing funding of breast cancer research and creating treatment, prevention, and screening programmes for lower-income women
Shi, 2014 [49] Case study In June 2012, Feng Jianmei, a 23-year-old Chinese woman, was forced to have late-term abortion by local birth-control officials	Health topic: Birth control; Target population: Public, government Organiser: Family members of Feng Jianmei Country: China	Not reported	Format: Graphic photos of the woman with the aborted fetus, micro-blogs and online forums Characteristics of narratives: Not reported	Jianmei's story was exposed by family members and went viral, provoking public outrage and widespread condemnation on social media. Story was quickly picked up by major media outlets in China and subsequently spread overseas. Provincial and municipal governments were under great pressure from the public	Agenda-setting: The National Population and Family Planning Commission announced that it would send 10 inspection teams to 19 provinces to review the policy enforcement of local family planning officials
Slaton et al., 2012 [50] Case study Timeframe not reported National family movement has advocated for meaningful engagement of families and youth who are the focus population of the federal Children’s Mental Health Initiative	Health topic: Children’s Mental Health Initiative Target population: Legislators Organiser: Children’s mental health family movement Country: United States of America	Not reported	Format: First person accounts of a family leader’s involvement in legislative policy as a result of her son’s mental health Characteristics of narratives: Not reported	Strategies that support effective family leadership at making macro-level improvements, such as using data to increase impact of personal stories; asking legislator to make a call on behalf of parents to whatever entity may have created a barrier to care for their child; designing a range of simple ways for parents to directly contact legislators about pending bills; devising ways to funnel information from large numbers of parents into family-run organisation’s public statements; working with the media; working with other groups in a coalition	Policy adoption: Legislators direct their staff to call family-run organisations to better understand how certain provisions will affect children with mental health needs and their families. Parents have been invited to see legislation they have worked on signed by the governor
Trossman, 1999 [55] Case study In January 1999, nurse Karen Daley got infected with HIV and hepatitis C as a result of being stuck bycontaminated needle while performing her job as an emergency room nurse	Health topic: Needle stick legislation Target population: Legislators and nurses Organiser: Nurse Karen Daley Country: United States of America	Not reported	Format: Testimony of nurse Karen Daley, President of Massachusetts Nurses Associations, who is now HIV- and hepatitis C-positive Characteristics of narratives: Not reported	On April 6, Nurse Daley walked into the Massachusetts State House to present her testimony and let legislators who consider the merits of a proposed needlestick bill to see first-hand that behind every injury, there is a real person. The testimony was covered by press and she has appeared on the public television show “Greater Boston”	Policy adoption: The day after Daley testified, the Massachusetts health committee put its support behind the bill, allowing it to move forward in the legislative process. The Massachusetts Nurse Association helped draft the language use in the House Bill 969
Umuhoza, 2013 [45] Case study In 2009, Rutgers WPF, a Dutch NGO working on sexual and reproductive health and rights supported work on these issues in the global South. A study tour in the Netherlands for six Youth Action Movement (YAM) from Rwanda, Malawi, Mali, Tanzania and Bangladesh was organised. YAM Rwanda’s members decided that their national action plan was going to focus on the issue of safe abortion. Throughout 2010 and 2011, YAM Rwanda took the challenge to improve the situation related to abortion in Rwanda	Health topic: Safe abortion Target population: Ministries, Parliamentarians, civil society organisations, public Organiser: Rwandan Youth Action Movement Country: Rwanda	Not reported	Format: Booklets with testimonies of young women in prison Characteristics of narratives: One of the testimonies read: “*My name is Anne. I am 20 years old… I have been in Karubanda prison since 2007 for committing abortion. I am the 3rd born in the family and the only girl; I was raised by my dad after my mum died when I was still young. I was in the 5th year of my secondary education when a teacher started dating me. I needed school materials and since I could not afford them, I allowed to have sexual intercourse with this teacher at that tender age. With limited knowledge of contraceptive use, I got pregnant and had to drop out of school since it’s against regulations. I decided to have an abortion. I am supposed to serve a period of 9 years of which I have so far completed 3*”	Booklets were distributed at different government offices and were also used by Rwandan Youth Action Movement members during strategic and political follow-up events in 2011. Booklets were complemented by a range of activities to achieve success, including data on extent of unsafe abortion; organised debates; value-clarification exercises; interviews and a survey; launching of a petition for law reform; production of awareness-raising materials; media engagement; and meetings with representatives from government ministries, the national women’s and youth councils, and parliamentarians	Policy adoption: Activities played a significant role in the advocacy process for amendment of the law, which was revised when the penal code came up for review in June 2012. Efforts coincided with important policy events, like the revision of Rwanda’s penal code
Wilcock et al., 2013 [47] Case study Timeframe not reported To initiate quality improvements within the NHS Modernisation Agency collaborative programmes that are patient- and carer-focused, it is critical to first identify their needs and concerns	Health topic: Quality improvement in healthcare Target population: NHS Modernisation Agency collaborative programmes Organiser: CHD Collaborative and Critical Care Collaborative Country: United Kingdom	Narrative is described as “*a story that tells a sequence of events that are significant to the narrator and his or her audience. Every narrative describes a sequence of events that have happened*”	Format: Verbal (narrated) elicited through Discovery Interview Process, a technique for listening to patients and carers and using their narratives to improve care Characteristics of narratives: Not reported	Patient narratives were gathered by trained interviewers and subsequently analysed by the NHS staff who provide their care. Stories were read out at a national meeting of the Critical Care Programme and the Coronary Heart Disease Collaborative at a plenary session attended by professionals throughout England	Policy implementation: Narratives led to patient-centred changes as part of a quality improvement project, e.g. in an East London hospital, nurses began to spend time talking with patients who were due to be moved, explaining the reasons and what the patient might expect. Improvements were also made at the pre-assessment phase of an elective patient journey, with introduction of nurses who visit all preoperative patients, identifying particular needs and talking to them about the critical care environment

The study designs were case studies (*n* = 15) [33, 43–56], participatory action research (*n* = 1) [57], documentary analyses (*n* = 1) [58] and biographical methods (*n* = 1) [59]. Thirteen studies were from high-income countries (Australia (*n* = 4), Republic of Ireland (*n* = 1), United States of America (*n* = 8) and United Kingdom (*n* = 1)), four studies were from middle-income countries (Georgia (*n* = 1), China (*n* = 1), and South Africa (*n* = 2)) and one study was from low-income countries (Rwanda (*n* = 1)); one study included data from both Republic of Ireland and Australia [43].

Eight studies targeted policy-makers, legislators and/or governors only [44, 46, 48, 50, 52, 53, 55, 59], one targeted national healthcare organisations only [47], and nine targeted multiple stakeholders including government and the public [33, 43, 45, 49, 51, 54, 56–58]. In one of the studies, the target audience was TV viewers; however, we only focused on the findings specific to policy-makers which was relevant to our question [53].

The included studies covered the topics of solarium tanning (*n* = 1), HIV/AIDS (*n* = 1), individuals with disabilities (*n* = 2), patient navigators for chronic diseases (*n* = 1), supervised injecting facilities for drug users (*n* = 1), abortion (*n* = 2), Medicaid Optional Coverage for Women and Infants programme (*n* = 1), needlestick injuries (*n* = 1), safety-net hospital for poor and uninsured residents (*n* = 1), birth control (*n* = 1), mental health initiatives for children (*n* = 1), cancer (*n* = 2), use of morcellators in gynaecologic surgeries (*n* = 1), approval of mifepristone for use as treatment for meningioma (*n* = 1), and quality improvement in healthcare (*n* = 1). One study focusing on personal celebrity health narratives examined a range of diseases, including multiple sclerosis, cancer, Parkinson’s disease, Alzheimer’s disease, HIV, Hugh Downs, bipolar II disorder, eating disorder, type 1 diabetes, paralysis, cystic fibrosis and amyotrophic lateral sclerosis [58].

Four of the included studies provided a definition for narrative [33, 44, 47, 58] and three described the theoretical framework underpinning the narrative intervention [44, 57, 58]. All but four studies used a meta-narrative, which combined the stories of a large number of people to convey a thematic, systemic story as opposed to focusing on a single event or individual (i.e. episodic stories). The narrative information was presented in different formats, with some studies utilising more than one format. These formats included television appearances (*n* = 3) [54, 58, 59], entertainment education (prime-time network TV storyline) (*n* = 1) [53], short films, theatre and plays (*n* = 1) [43], magazines and journal prints (*n* = 2) [56, 58], books (*n* = 2) [33, 52], narrative action reflection workshops (*n* = 1) [57], video materials (*n* = 2) [44, 56], posters and photos (*n* = 3) [44, 49, 51], booklets with testimonies (*n* = 1) [45], advocacy summaries of personal experiences (*n* = 1) [46], story cards with photos of patients on front and quotes on back (*n* = 1) [48], micro-blogs and online forums (*n* = 1) [49], and verbal (narrated) (*n* = 6) [33, 47, 50, 55, 58, 59].

In seven studies, the narrative information was presented alone [33, 47, 51, 52, 55, 58, 59], whereas in 11 studies, the narrative was part of a multi-component intervention leading to the reported change in outcome [43–46, 48–50, 54, 56, 57]. Components besides narratives included organised debates, values clarification exercises, interviews and surveys in four universities, a petition for law reform and media involvement [45]; quantitative data [46]; state-wide campaign, writing to governor, lieutenant governor, or speaker of the House, talking one-on-one with the legislators, and inviting legislators to gain first-hand experience [48]; parental meetings, working with other groups in a coalition, quantitative data and media engagement [50]; research and media appearances [43]; seminars, documentation and conferences [57]; trials, reports, royal commissions and advocacy efforts [44]; media advocacy, direct political representation and lobbying and epidemiological evidence [54]; and campaign, media coverage, and review of research [56].

The included studies examined the influence of narratives on the policy outcomes of agenda-setting (*n* = 5), policy formulation (*n* = 1), policy adoption (*n* = 9), and policy implementation (*n* = 4); none looked at policy evaluation. One of the studies included data from two countries, and reported impact at different levels of the policy-making process [43]. None of the studies conducted actual evaluation of effectiveness or provided explicit evidence for the link between the intervention and the outcome.

### Methodological appraisal

Additional file 3 presents the detailed assessment of the reporting of included case studies according to the standards by Rodgers et al. [41]. None of the cases studies met all 13 reporting standards. The median score was 4 (out of a maximum score of 13). Only four studies met more than half of the reporting standards [43, 44, 47, 54].

### Influence on policy outcomes

We present below the findings stratified according to the stages heuristic framework of the public policy process.

#### Agenda-setting

One participatory action research [57], one documentary analysis [58] and three case studies [43, 49, 51] examined the influence of narrative on agenda-setting.

Lorenzo [57] reported that a series of narrative action reflection workshops enabled storytelling and action learning by women with disability in Cape Town. These women were able to mobilise collectively for change regarding an accessible public transport system as a strategy for social inclusion. The findings have “*contributed to increasing the understanding and awareness of disability issues for people involved in policy development and implementation across the various sectors of government to improve service delivery mobilizing for an accessible public transport system so that they had equal opportunities to participation in social and economic development*” [57].

Johnson et al. [43] examined the contribution of inclusive qualitative research studies (via life stories) to policy-making related to people with disabilities’ right to relationships and to a sexual life in Ireland. As a result of research, drama and media appearances, the issue of the prohibition of penetrative sexual activity with a person with intellectual disabilities under Irish law was brought to public attention. In March 2011, two representatives from the research group were invited to consultations with the government’s legislative advisory body, the Law Reform Commission, with regard to reviewing the 1993 Act – “*The Act in the Republic has not yet been changed, but there is now a significant voice from people with intellectual disabilities informing discussion of how this might happen*” [43].

Beck et al. [58] conducted a documentary analysis of newspaper articles, letters to the editors of magazines and newspapers, televised interviews, and online databanks to identify celebrities, including athletes, actors, musicians and politicians, who have publicly shared personal narratives regarding their health situation (or that of a loved one). The authors found that the key functions that celebrity health narratives performed were education, inspiration and activism. Celebrity narratives have contributed to raising money and awareness and have also led to doubling funding for research at the National Institutes of Health for certain diseases.

Krueger [51] conducted a case study of how cancer-related organisations were integrating, beginning in the late 1940s, the voices, images and stories of young sufferers into their annual campaigns as tools for education, awareness and inspiration. The author remarked: “*Poster children were strategically used throughout the mid-to-late twentieth century to advance principles of early cancer detection and prompt treatment; to illustrate or, at times, exaggerate promising biomedical advances in the field; and to elicit emotional responses and donations from a wide audience during the escalation of the war against cancer”* [51].

Shi [49] described the story of Feng Jianmei, a 23-year-old Chinese woman who was forced to have late-term abortion by local birth-control officials. Her story was exposed by family members through graphic photos, micro-blogs and online forums, provoking public outrage and widespread condemnation on social media sites. In response to tremendous pressure from the public, the National Population and Family Planning Commission announced that it would send 10 inspection teams to 19 provinces to review the policy enforcement of local family planning officials.

#### Policy formulation

Johnson et al. (discussed above) [43], explored the contribution of inclusive qualitative research studies to change in policy and legislation related to people with disabilities’ right to relationships and to a sexual life, also in Australia. Twenty-five people with intellectual disabilities told their life stories and talked about sexuality and relationships. Three short films of the stories were subsequently produced and used to lobby government for a change to the existing policy. The new policy draft clearly set out rights and responsibilities for people with intellectual disabilities and service providers about relationships and sexuality.

#### Policy adoption

Eight case studies [44–46, 48, 50, 53–55] and one biographical study [59] examined the influence of narrative on policy adoption.

MacKenzie et al. [54] reviewed television and print media coverage of the campaign to regulate solaria initiated by Clare Oliver before her death from melanoma in late 2007. A frame analysis was conducted of all direct and attributed statements about the causes of, and responsibility for, Oliver’s melanoma, and about the legacy of her campaign. Oliver’s story was influential in securing regulations across all states and territories to ban access to tanning beds for those younger than 18 years.

Marcus et al. [53] assessed the educational and behavioural impact of an *ER* (NBC drama) storyline that addressed cancer patient navigators on primetime TV viewers. Clips of the TV episode were used by Congressional staffers to raise awareness of patient navigators in a Congressional Committee meeting. Congressional staffers reported that the episode provided a tangible example of how patient navigator programmes would work and contributed to “*critical policy discussion that led to passage of US law HR 1812, the Patient Navigator Outreach and Chronic Disease Prevention Act of 2005*” that provides funds to model programmes that would help patients to access healthcare services.

Fitzgerald [44] retraced the policy narratives related to the introduction of supervised injecting facilities (SIFs) in Australia in documentary materials. Narrative was used as an advocacy tool to inform government of the impact of street injecting on a local community. The narrative intervention was one component of a multi-faceted intervention, including trials, reports and advocacy to promote SIF in two different parts of Australia (Sydney and Melbourne). In Sydney, SIF policy development was framed as a response to police corruption (i.e. police were viewed as villains), whereas in Melbourne, the policy narratives focused on health and welfare of drug users (with the police, here, viewed as heroes). These two framings created a different pathway for policy development. In Sydney, 22 months after the Drug Summit Legislative Response Act 1999 was passed for the trial of the SIF, the service opened in May 2001. In Melbourne, the bill failed to pass through both Houses of the Victorian Parliament.

Umuhoza et al. [45] reflected on the use of personal stories as advocacy tools to mobilise action for law reform. Narrative was part of a multicomponent intervention initiated by Rwandan Youth Action Movement to put the new abortion law into effect in 2012. The use of testimonies and stories of young people gave a ‘face’ to the issue of unsafe abortion. These activities “*played a significant role in the advocacy process for amendment of the* [abortion] *law which was revised when the penal code came up for review in June 2012*” [45]. These efforts also coincided with important policy events, which opened a window of opportunity for action.

Leith and Phillips [46] presented three short case studies illustrating how qualitative techniques and consumer narratives can serve as advocacy tools to inform public policy. We focused on the case study on Optional Coverage for Women and Infants Medicaid programme as it is health related. To demonstrate to state legislators the need for continuing the programme, the state agency in a south-eastern state provided state legislators with advocacy summaries from their constituent areas. Hotline information was used to create thousands of these advocacy summaries consisting of brief narratives of three to eight sentences that gave a qualitative description of the personal experiences of callers with the system. The narratives were also supplemented by data regarding the success of the programme. Based on these narrative accounts, “*legislators were able to see first hand the impossible situations families in poverty were experiencing, and the Optional Coverage for Women and Infants program was ultimately spared from proposed legislative budget cuts*” [46].

Neuhausen [48] reported how hundreds of students and resident physicians fought for the survival of ‘Grady’, the fifth-largest public hospital and the largest provider of indigent care in Georgia. Narrative was one component of a national campaign and advocacy efforts to get state legislature to approve the non-profit conversion of Grady and to increase state funding. Stories from Grady patients describing the life-saving care provided by Grady were collected to create story cards, with photos of patients on the front and quotes on the back. These stories were described as the most powerful advocacy tool. The commissioners voted to approve the non-profit conversion which was critical to Grady’s survival. The Georgia General Assembly also approved “*increases in Medicaid reimbursement rates to trauma hospitals and authorized $58 million to support uncompensated care at state trauma centers*” [48].

Slaton et al. [50] described the stories of four family-led organisations and the impact of their advocacy efforts on systems of care for mental health. We specifically focused on one family leader’s story of involvement in legislative policy. Narratives of parents of children with mental health problems was one component of a multifaceted approach that also included networking with other families and organisations, speaking with legislators, engaging in advocacy and involving the media. According to the family leader involved in that case study, legislators now direct their staff to call the family-led organisation to better understand how certain provisions will affect children with mental health needs and their families. Additionally, parents have been invited to see legislation they have worked on being signed by the governor [50].

Trossman [55] described how the President of Massachusetts Nurses Associations used her personal story to push for needlestick legislation in Massachusetts. Following her infection with HIV and hepatitis C as a result of being pricked by a contaminated needle, Nurse Daley presented her testimony at the Massachusetts State House and let legislators who consider the merits of a proposed needlestick bill to see that there is a real person behind every injury. The day after she testified, the Massachusetts health committee put its support behind the bill, allowing it to move forward in the legislative process.

Lander [59] described how her campaign to amend the legal status of the drug mifepristone in Australia was triggered in 2005 when she was diagnosed with meningioma. Although the drug could halt the growth of tumours, it was unapproved in Australia because of its potential use as an abortifacient. The campaign involved email exchanges and ongoing contact with a Senator as well as participation in broadcasts to help raise awareness of the drug. In 2006, parliament successfully voted to repeal ministerial responsibility for approval of the drug mifepristone.

#### Policy implementation

Four case studies examined the role of narratives on policy implementation [33, 47, 52, 56].

MacGregor and Mills [52] described how, in an attempt to enhance access to antiretroviral drugs in South Africa in 2003, a group of women involved with the Treatment Action Campaign and Medicines Sans Frontières participated in an initiative to ‘map’ their bodies as affected by HIV. Through the body maps and personal accounts published alongside the maps, women told how their lives have been transformed by antiretroviral treatment. The women’s body maps and narratives were published in a book, ‘Long Life’, which was subsequently used as a political tool to add the ‘voices’ of ordinary people to the campaign. These efforts contributed to the roll out of universal access to antiretroviral treatment drug in the public sector.

Wilcock et al. [47] reflected on the use of patient stories to inspire quality improvement within the NHS Modernisation Agency collaborative programmes. Patients’ narratives were gathered by trained interviewers in one-to-one semi-structured qualitative interviews and analysed by the NHS staff who provide their care. The stories were read out at a national meeting of the Critical Care Programme and the Coronary Heart Disease Collaborative. The authors presented two case studies illustrating how these narratives led to patient-centred changes as part of quality improvement projects within the NHS Modernisation Agency collaborative programmes in England.

Sharf [33] provided a historical view of how personal narratives of women living with breast cancer affected health policy. The author reported that personal breast cancer stories have inspired efforts by citizen advocates and legislators to provide better care and more resources for the disease (for example, changing of standard clinical procedure and creating treatment, prevention and screening programmes for lower-income women). However, the author also shed light on a case where narrative of breast cancer led to undesirable outcomes; in 1993, after being diagnosed with advanced breast cancer, Nelene Fox was advised by her doctors that her only remaining chance for survival was an autologous bone marrow transplant. Her insurance refused to pay for the procedure because the treatment was classified as experimental due to insufficient scientific evidence that it extended a patient’s life. Fox’s brother, a lawyer, sued the insurance and convinced the jury to award $89,000 in damages to her family. Media publicity about the Fox case succeeded in forcing widespread insurance reimbursement, further discouraging patients from enrolling in clinical trials. Conclusions about the efficacy of the treatment were delayed until 1999 when the National Cancer Institute announced that autologous bone marrow transplant does not benefit people with breast cancer.

Rosenbaum [56] reflected on the case of 40-year-old Amy Reed who underwent a hysterectomy with intraoperative morcellation for presumptively benign uterine fibroids (which unknowingly contained leiomyosarcoma), thus causing it to disperse. Following her death, Reed’s husband launched a campaign to ban morcellators which was picked up by *The Wall Street Journal.* Extensive media coverage featuring the faces of women dying of leiomyosarcoma exaggerated the risk of leiomyosarcoma, while the benefits of morcellation remained largely invisible and, thus, ‘unavailable’. Subsequently, the Food and Drug Administration undertook a review to quantify the risk of disseminating occult uterine cancers that cannot be reliably detected preoperatively, 6 months later issuing a black-box warning stating that morcellation was contraindicated in perimenopausal or postmenopausal women and in “*candidates for en bloc tissue removal*”. Many institutions banned morcellation and some insurers stopped covering the procedure or began requiring prior authorisation. The author explained that medical products are associated with two types of risk, those caused by using the products and those caused by preventing their use; the morcellation controversy is an example of the latter case given that “*there may be greater population benefits and lesser risks from continuing than from discontinuing morcellator use*” [56]. However, disproportionate focus on harms caused by use rather than non-use skewed risk perception*.*

#### Policy evaluation

We found no studies examining the role of narratives in policy evaluation.

### Knowledge gaps

Our systematic review highlighted key knowledge gaps concerning the use of narrative interventions in health policy-making. We present, in Table 4, the knowledge gaps relevant to the study design, interventions, setting, population and outcomes in the area of using a narrative to impact health policy-making [42].

**Table 4:** Identified knowledge gaps

Domain	Identified knowledge gaps
Study design	• Lack of experimental or interventional studies on the impact of narratives on health policy-making in real-life settings • Lack of qualitative studies to better understand the knowledge, beliefs and attitudes of policy-makers towards narratives
Intervention	• Limited evidence on the effects of narratives (as standalone) independent of other interventions (e.g. the use of social media) • Limited description of the narrative intervention (including frequency and duration of exposure, content of narrative (e.g. plot and characters) and perceived credibility of speaker of message) • Lack of evidence comparing different narrative formats (e.g. verbal print, audio, video) and perspectives (first- versus third-person narrative) • Limited description of the framework or theory guiding intervention development • Variations in definitions and operationalisation of narrative information
Study setting	• Limited evidence on the effects of narratives on health policy-making across cultural settings and countries • Limited evidence from low-income countries • Lack of evidence from the Eastern Mediterranean Region, Region of the Americas and Western Pacific Region
Population	• Lack of evidence on the effect of moderators, such as policy-makers’ characteristics
Outcomes	• Limited evidence on the impact on policy stages (in general) • Lack of evidence evaluating the impact on policy evaluation stage • No valid measurement of the impact on the outcomes of interest (e.g. no actual evaluation or assessment based on perceptions of respondents)

## Discussion

### Summary and interpretation of findings

This systematic review identified 18 eligible studies examining the effects of narratives on the different stages of the health policy-making process, except for the policy evaluation stage. The vast majority of included articles describe case studies.

The existing evidence base precludes any robust inferences about the impact of narrative interventions on health policy-making. Nonetheless, the findings suggest that narratives may have a positive influence when used as inspiration and empowerment tools to stimulate policy inquiries; as educational and awareness tools to initiate policy discussions (and gain public support leading to policy prioritisation); and as advocacy and lobbying tools to formulate, adopt or implement policy.

However, there is also evidence of undesirable effects of using narratives [33, 51, 56]. In one case study, narrative use led to widespread insurance reimbursement of a therapy for breast cancer that was later proven to be ineffective [33]. Another case study described how the use of narrative inappropriately exaggerated the perceived risk of a procedure, which led to limiting its use and preventing a large number of patients from its benefits [56]. A third case study described how optimistic ‘cure’ or ‘hope’ stories of children with cancer were selectively used to raise money for cancer research that ignored the negative realities such as limited gains made in certain paediatric cancers, the high costs of treatment, and the high prevalence of mental and physical disabilities caused by experimental chemotherapy protocols [51].

The majority of included studies did not provide information on the definition of narratives, the theoretical framework underlying the narrative intervention or the possible predictors of the success of narrative-based interventions. Only one study explicitly discussed how the framing of the attributes of narratives (i.e. sequenced events, characters, time, location, etc.) influenced policy-making differentially in two different parts of Australia (Sydney and Melbourne) [44]. Thus, uncertainties remain about how to construct and present narrative information.

Having said this, we highlight two emerging patterns that might inform the optimal use of narratives. First, all but four studies used a meta-narrative that combined the stories of a large number of people to convey a thematic, systemic story as opposed to focusing on a single event or individual (i.e. episodic stories). Second, the importance of establishing a relationship with media outlets and maximising opportunities to disseminate the narrative information was emphasised in several studies. Indeed, six of the included studies highlighted the involvement of media as an important catalyst for policy change [43, 45, 49, 50, 54, 56]. In four studies, the narrative was picked up by media [43, 45, 49, 56] whereas, in two studies, active effort was made to engage the media and maximise the reach of the narrative information [50, 54]. Unfortunately, there is also a lack of reliable evidence on the use of media interventions to influence health policy-making [60].

### Strengths and limitations

To our knowledge, this is the first systematic review examining the effects of narrative-based interventions on the health policy-making process. Strengths of our methodology include the pre-publication of a protocol, a rigorous and transparent review process, and adherence to standard methods for reporting systematic reviews [61, 62]. In addition, we searched multiple databases and included both published and the grey literature.

A major challenge in conducting this review was how to best conceptualise and categorise narratives given the absence of clear definition and operationalisation of narrative information. This made it difficult to decide on the eligibility of some of the studies and to abstract data. This is why we relied on a consensus approach to screening and data abstraction, and iteratively revised our conceptualisation of narrative. Although we did search resources that include grey literature (e.g. Google Scholar and the WHO Global Health Library and Communication and Mass Media Complete), we could have searched additional resources such as websites of NGOs, advocacy groups and donors. Further, while some might criticise our use of stages heuristic framework, considered by some scholars to assume linearity of the policy-making process, we opted to use this framework as it is considered one of the most prominent public policy frameworks. More importantly, it did facilitate synthesis of findings and provide a simplified and useful way to view the entire policy process [37, 38].

### Knowledge gaps

A major gap relates to the poor methodological rigor of the included studies. All included studies lacked actual evaluation of effectiveness or explicit evidence for the link between the intervention and the outcome. Additionally, in the majority of the included studies, the narrative component was part of a multicomponent intervention, and thus the evidence associated with the narrative may be indirect or confounded by other components of the intervention [34]. The aforementioned limitations made it difficult to make any inferences on effectiveness of narrative interventions in the health policy-making process.

Moreover, the very limited description of the narrative interventions was challenging, particularly given that they qualify as complex interventions [63, 64]. Important aspects of these interventions include frequency and duration of exposure to a narrative, content of a narrative (for example, plot, characters, and moral of story) and perceived credibility of the speaker or message. These aspects are important to understand the specific narrative intervention that was tested, what component of the intervention was effective, and the superiority of one format over another.

Additionally, the majority of included studies failed to provide information about what framework or theory guided intervention development and outcome measurement. Several theories of narrative persuasion have been identified in the literature [15, 17, 18]. Thus, without such information, it would be difficult to understand the mechanism by which narrative interventions persuade health policy-makers and lead to change. Moreover, we could not determine the effect of moderators, such as the policy-makers’ characteristics on the effectiveness of the narratives.

While beyond the scope of this systematic review, we have identified studies assessing message-framing involving hypothetical scenarios on the attitudes, beliefs and intentions of health policy-makers and legislators [65–68]. These warrant further exploration to complement the evidence base on the use of narratives in health policy-making in real settings.

### Comparison to other systematic reviews

While we did not identify any other systematic review of narratives in the field of health policy-making, we identified many in the field of clinical decision-making [2, 21, 69]. Perrier and Ginis found “*consistent evidence supporting the efficacy of narratives at changing screening behavior*” [69], but mixed evidence supporting an advantage of narratives over providing statistical information for screening behaviour and its determinants. Winterbottom et al. [2] found limited evidence suggesting that narratives affected an individual’s medical decisions, but it was unclear why narratives affected the decision-making process or whether they facilitated or biased decision-making. Bekker et al. [21] concluded that there is insufficient evidence that adding personal stories to patient decision aids enhances their effectiveness to support people to make informed decisions.

### Implications for research

Despite the increased interest in narratives [6, 23, 24, 70], the evidence base on their impact on health policy-making is of very low certainty. This systematic review highlights the challenges of assessing the impact of narrative interventions on health policy-making given the complex nature of these interventions, the difficulties in using experimental methods, and the multiple factors influencing the policy-making process [34, 64, 71]. Therefore, more rigorous primary research is needed to gain a better understanding of narrative interventions beyond whether or not they are effective to why and under what circumstances.

Given that narratives qualify as complex interventions, a particular focus should be on conducting realist evaluation studies. Unlike traditional impact evaluation that establishes whether change in outcomes can be directly attributed to an intervention, realist evaluation focuses on the processes and contexts of implementation that yield impact [72]. Thus, by examining ‘what works, for whom and why’, insights are gained about the interactions between interventions, implementers and health systems that make interventions more or less successful [73, 74]. This is critical to inform the design of context-specific strategies and understand how the influence of narrative information can differ across various health systems and socioeconomic realities. Additionally, qualitative studies can help explore the knowledge, beliefs and attitudes of policy-makers towards narrative information, including their role in the policy-making process.

In addition, researchers are encouraged to promote better reporting of studies in this field, taking into account guidelines for reporting of complex interventions when describing the narrative interventions [75, 76]. Importantly, experts in the field should establish clear definition and operationalisation of narratives to allow better research and communication on the topic.

Funding agencies have an important role in advancing knowledge and un-tapping the potential for using narrative to influence health policy-making by supporting studies that address the aforementioned knowledge gaps. This would also align with global calls for more effective and innovative approaches to bridge the gap between research and policy-making [77–80].

### Implications for policy

Our findings suggest that, while narratives may have a positive influence on health policy change, they may sometimes lead to undesirable outcomes. Findings also allude to potential pitfalls and ethical concerns that should be taken into consideration when using narratives. First, because of the selective nature of narratives, narrators may omit details of a story or exaggerate it, so the story may not be representative of the larger reality (for example, the case of childhood cancer where optimistic ‘cure’ or ‘hope’ stories were selectively used that ignored the negative realities). Second, the reliance on narratives without scientific evidence may lead policy-makers to adopt policies that may be ineffective or even harmful or waste resources (for example, the case of reimbursing a therapy for breast cancer that was later proven to be ineffective). Third, narratives may produce biased results based on the views of one or a select number of individuals (for example, the case of discontinuing morcellator use). Because of the affective nature of narratives, policy-makers may give higher priority to diseases with more tragic stories such as cancer and HIV at the expense of other diseases with a similar or higher burden such as cardiovascular diseases [33, 58].

These limitations and potential pitfalls do not mean that there should be no place for narratives in informing health policy-making. However, it does mean that narratives need to be held to standards of validity [6, 81, 82]. For instance, Hyman [83] insists that those using narratives provide persuasive evidence of typicality and completeness before assigning any weight to their stories. Sharf [33] calls for effectively combining the emotional pathos of stories with rhetorical proof.

In light of the above, we suggest using narratives that are rooted in evidence to influence health policy-making. Those designing or using narrative information need to consider all the above discussed challenges and potential pitfalls. This would be best achieved by building strong and effective partnerships between ‘evidence experts’ and those involved in advocacy. Additionally, narratives could be used to support policies that are based on widely agreed on principles, such as those of human rights and medical ethics like access to basic health services and non-discriminatory health policies.

## Conclusion

Despite the increased interest in narratives, the existing evidence base precludes any robust inferences about the impact of narrative interventions on health policy-making. Rigorous research supporting impact on health policy-making is still needed.

## Additional files

Additional file 1: Search strategy. (DOCX 21 kb)

Additional file 2: Excluded studies and reasons for exclusion. (PDF 767 kb)

Additional file 3: Assessment of the reporting of included case studies. (PDF 338 kb)
