# Supplementary material for: Using narratives to impact health policy-making: a systematic review
Source: Health Res Policy Syst. 2019 Mar 5;17:26. doi: 10.1186/s12961-019-0423-4 (PMC6402129; doi:10.1186/s12961-019-0423-4)
Supplement: Supplementary file 1 — Search strategy. (DOCX 21 kb) [file 12961_2019_423_MOESM1_ESM.docx]

Additional file 1: Search strategy

| **Databases** | **# Hits** | **Date of search** |
| --- | --- | --- |
| Medline | 6022 | February 15 2017 |
| Global Health Library | 5653 | February 15 2017 |
| CINAHL | 3576 | February 16 2017 |
| PSYSCH INFO | 1478 | February 16 2017 |
| Communication and Mass Media | 1900 | February 16 2017 |
| Cochrane | 75 | February 16 2017 |
| Google scholar | 100 | February 16 2017 |
| **Total** | **18, 804** |  |
| **Duplicates** | **6,106** |  |
| **Total after removing duplicates** | **12, 698** |  |

Database: **Ovid MEDLINE(R)** Epub Ahead of Print, In-Process & Other Non-Indexed Citations, Ovid MEDLINE(R) Daily and Ovid MEDLINE(R) <1946 to Present>

Search Strategy:

--------------------------------------------------------------------------------

1 exp personal narratives/ (5990)

2 exp Narration/ (6558)

3 exp anecdotes as topic/ or exp personal narratives as topic/ (4903)

4 (narrative* or narration# or testimonial# or anecdot* or exemplar* or story* or stories).ti,ab. (69364)

5 1 or 2 or 3 or 4 (81623)

6 policy/ or smoke-free policy/ or social control policies/ or public policy/ or health policy/ or health care reform/ or lobbying/ or Jurisprudence/ or environmental policy/ or family planning policy/ or nutrition policy/ or legislation as topic/ or exp legislation, drug/ or legislation, food/ or legislation, hospital/ or legislation, medical/ or legislation, nursing/ or legislation, pharmacy/ or social control, formal/ or government regulation/ or law enforcement/ (234682)

7 exp Policy Making/ (22073)

8 government/ (12693)

9 Decision Making/ (79286)

10 8 and 9 (402)

11 Government Programs/ (4318)

12 ((government* or national) adj2 (decision* or regulation# or program#)).ti,ab. (4038)

13 (agenda* adj3 setting*).ti,ab. (648)

14 (law or laws or legal or legislation# or decree# or jurisprudence).ti,ab. (148686)

15 (policymak* or policy-mak* or policy or policies).ti,ab. (193972)

16 exp child advocacy/ or exp consumer advocacy/ (7148)

17 patient advocacy/ (23290)

18 (clinical or treatment).ti,ab. (5535740)

19 17 not 18 (19978)

20 ((health* adj2 (reform or reforms or reforming)) or lobby* or advocacy or advocating or advocacies or advocate).ti,ab. (44027)

21 6 or 7 or 10 or 11 or 12 or 13 or 14 or 15 or 16 or 19 or 20 (554664)

22 5 and 21 (**6022**)

***************************

**Global health library**

*Two separate searches were conducted, and results combined*

Search 1:

(ti:((narrative* or narration* or testimonial* or anecdot* or exemplar* or story* or stories) and ((government* regulation*) or (government* decision*) or (government* polic*) or (national decision*) or (national regulation*) or (national polic*)))) OR (ab:((narrative* or narration* or testimonial* or anecdot* or exemplar* or story* or stories) and ((government* regulation*) or (government* decision*) or (government* polic*) or (national decision*) or (national regulation*) or (national polic*)))) : **1018**

Search 2:

(ti:((narrative* or narration* or testimonial* or anecdot* or exemplar* or story* or stories) and (policy* or policies or law or laws or legislation* or legal* or decree or decrees or jurisprudence or advocacy or advocacies or advocate or advocating or lobby*))) OR (ab:((narrative* or narration* or testimonial* or anecdot* or exemplar* or story* or stories) and (policy* or policies or law or laws or legislation* or legal* or decree or decrees or jurisprudence or advocacy or advocacies or advocate or advocating or lobby*))) : **4635**

**CINAHL**

| **#** | **Query** | **Results** |
| --- | --- | --- |
| S18 | S3 AND S17 | **3,576** |
| S17 | S15 OR S16 | 273,556 |
| S16 | TI ( (policy* or policies or law or laws or legislative or legislatives or legislature or legislatures or legislation or legislations* or decree or decrees or jurisprudence or (agenda* setting*) or (government* regulation) or (government* regulations) or (government* decision*) or (national decision*) or (national regulation) or (national regulations) or (public decision*) or (health* advocacy) or (health* advocacies) or (health* advocate*) or (health* lobby*)) ) OR AB ( (policy* or policies or law or laws or legislative or legislatives or legislature or legislatures or legislation or legislations* or decree or decrees or jurisprudence or (agenda* setting*) or (government* regulation) or (government* regulations) or (government* decision*) or (national decision*) or (national regulation) or (national regulations) or (public decision*) or (health* advocacy) or (health* advocacies) or (health* advocate*) or (health* lobby*)) ) | 113,055 |
| S15 | S4 OR S5 OR S6 OR S7 OR S8 OR S9 OR S10 OR S11 OR S12 OR S13 OR S14 | 199,521 |
| S14 | (MH "Government Programs") | 4,691 |
| S13 | (MH "Health Care Reform") | 26,079 |
| S12 | (MH "Patient Advocacy") | 10,616 |
| S11 | (MH "Consumer Advocacy") OR (MH "Child Advocacy") | 5,379 |
| S10 | (MH "Lobbying") | 4,299 |
| S9 | (MH "Policy Making") | 8,481 |
| S8 | (MH "Government Regulations") | 13,323 |
| S7 | (MH "Jurisprudence+") | 70,336 |
| S6 | (MH "Legislation, Drug+") OR (MH "Legislation, Medical+") OR (MH "Legislation, Nursing+") OR (MH "Legislation, Hospital") | 8,912 |
| S5 | (MH "Legislation") | 15,731 |
| S4 | (MH "Public Policy+") OR (MH "Health Policy+") OR (MH "Nutrition Policy") | 79,908 |
| S3 | S1 OR S2 | 42,642 |
| S2 | TI ( (narrative* or narration or narrations or testimonial* or anecdot* or exemplar* or story* or stories) ) OR AB ( ( narrative* or narration or narrations or testimonial* or anecdot* or exemplar* or story* or stories ) ) | 35,421 |
| S1 | (MH "Narratives") OR (MH "Storytelling") OR (MH "Biographies+") | 14,545 |

**Psych Info**

| **#** | **Query** | **Results** |
| --- | --- | --- |
| S5 | S1 OR S4 | **1,478** |
| S4 | S2 AND S3 | 296 |
| S3 | ((((((DE "Policy Making" OR DE "Education Policy" OR DE "Health Care Policy" OR DE "Government Policy Making" OR DE "Abortion Laws" OR DE "Disability Laws" OR DE "Discrimination Laws" OR DE "Drug Laws") OR (DE "Law (Government)" OR DE "Law Enforcement" OR DE "Legal Decisions" OR DE "Legal Processes" OR DE "Legislative Processes")) OR (DE "Drug Laws")) OR (DE "Community Advocacy" OR DE "Government Policy Making" OR DE "Legislative Processes")) OR (DE "Health Care Reform")) OR (DE "Environmental Policy")) OR (DE "Social Control") | 70,331 |
| S2 | (DE "Narratives" OR DE "Storytelling") OR (DE "Biography") OR DE "Conversation" | 30,987 |
| S1 | TI ( (narrative* or narration or narrations or testimonial* or anecdot* or exemplar* or story* or stories) N10 (policy* or policies or law or laws or legislative or legislatives or legislature or legislatures or legislation or legislations* or decree or decrees or jurisprudence or (agenda* setting*) or (government* regulation) or (government* regulations) or (government* decision*) or (national decision*) or (national regulation) or (national regulations) or (public decision*) ) ) OR AB ( (narrative* or narration or narrations or testimonial* or anecdot* or exemplar* or story* or stories) N10 (policy* or policies or law or laws or legislative or legislatives or legislature or legislatures or legislation or legislations* or decree or decrees or jurisprudence or (agenda* setting*) or (government* regulation) or (government* regulations) or (government* decision*) or (national decision*) or (national regulation) or (national regulations) or (public decision*) ) ) OR SU ( (narrative* or narration or narrations or testimonial* or anecdot* or exemplar* or story* or stories) N10 (policy* or policies or law or laws or legislative or legislatives or legislature or legislatures or legislation or legislations* or decree or decrees or jurisprudence or (agenda* setting*) or (government* regulation) or (government* regulations) or (government* decision*) or (national decision*) or (national regulation) or (national regulations) or (public decision*) ) ) | 1,262 |

**Communication and Mass Media**

| **#** | **Query** | **Results** |
| --- | --- | --- |
| S11 | S6 AND S10 | **1,900** |
| S10 | S7 OR S8 OR S9 | 39,075 |
| S9 | TI ( (policy* or policies or law or laws or legislative or legislatives or legislature or legislatures or legislation or legislations or decree or decrees or jurisprudence or (agenda* setting*) or (government* regulation) or (government* regulations) or (government* decision*) or (national decision*) or (national regulation) or (national regulations) or (public decision*) or (health* advocacy) or (health* advocacies) or (health* advocate*) or (health* lobby*)) ) OR AB ( (policy* or policies or law or laws or legislative or legislatives or legislature or legislatures or legislation or legislations or decree or decrees or jurisprudence or (agenda* setting*) or (government* regulation) or (government* regulations) or (government* decision*) or (national decision*) or (national regulation) or (national regulations) or (public decision*) or (health* advocacy) or (health* advocacies) or (health* advocate*) or (health* lobby*)) ) | 36,538 |
| S8 | DE "POLITICAL science" | 2,939 |
| S7 | DE "PROCLAMATIONS" | 10 |
| S6 | S1 OR S2 OR S3 OR S4 OR S5 | 32,334 |
| S5 | TI ( (narrative* or narration or narrations or testimonial* or anecdot* or exemplar* or story* or stories ) ) OR AB ( (narrative* or narration or narrations or testimonial* or anecdot* or exemplar* or story* or stories ) ) | 31,527 |
| S4 | DE "REPORTAGE literature" | 39 |
| S3 | DE "ANECDOTES" | 435 |
| S2 | DE "NARRATIVES" OR DE "NARRATORS" | 1,374 |
| S1 | DE "NARRATION (Rhetoric)" OR DE "MULTIPLE person narrative" OR DE "NARRATIVE paradigm theory" OR DE "NARRATIVE discourse analysis" OR DE "FIRST person narrative" OR DE "SECOND person narrative" OR DE "NARRATIVE paradigm theory -- Research" OR DE "NARRATOLOGY" OR DE "TRANSMEDIA storytelling" | 1,820 |

**Cochrane**

#1 MeSH descriptor: [Narration] explode all trees 143

#2 MeSH descriptor: [Personal Narratives] explode all trees 0

#3 MeSH descriptor: [Personal Narratives as Topic] explode all trees 3

#4 MeSH descriptor: [Anecdotes as Topic] explode all trees 13

#5 #1 or #2 or #3 or #4 158

#6 (narrative* or narration* or testimonial* or anecdot* or exemplar*

or story* or stories) and (policy* or policies or law or laws or

legislation* or legal* or decree or decrees or lobby* or advocac*) 1755

#7 #5 or #6 1910

Limit to trials = **75**

**Google scholar**

(narrative* OR narration* OR testimonial* OR story* OR stories) AND (policy* OR policies OR law OR laws OR legislation* OR legal* OR decree OR decrees OR jurisprudence OR advocacy OR advocacies OR advocate OR advocating OR lobby*)

*The first 100 articles were retrieved, “sorted by relevance”*
