# Supplementary material for: Using narratives to impact health policy-making: a systematic review
Source: Health Res Policy Syst. 2019 Mar 5;17:26. doi: 10.1186/s12961-019-0423-4 (PMC6402129; doi:10.1186/s12961-019-0423-4)
Supplement: Supplementary file 2 — Excluded studies and reasons for exclusion. (PDF 767 kb) [file 12961_2019_423_MOESM2_ESM.pdf]

## Additional file 2: Excluded studies and reasons for exclusion

| Coded reason for exclusion | Reason for exclusion | Number of studies |
|---|---|---|
| 1 | Does not focus on planned narratives as the main intervention | **258** |
| 2 | Does not focus on health or health-related issues | **18** |
| 3 | Does not target population-level policy changes in a real world setting | **135** |
| 4 | Not study design of interest (e.g. commentary, review, magazine) | **25** |
| 5 | Duplicate information | **2** |
| **Total** | | **438** |

## Excluded studies

| Citation | Coded reason for exclusion | Additional comment |
|---|---|---|
| **(Abou Taam et al., 2014)** | **3** | |
| **(Abeysinghe & White, 2011)** | **1** | |
| **(Akintola, Lavis, & Hoskins, 2015)** | **1** | |
| **(K. A. Anderson, 2011)** | **1** | |
| **(Allison et al., 2016)** | **3** | |
| **(Anaf, Newman, Baum, Ziersch, & Jolley, 2013)** | **3** | |
| **(Andersen, 2013)** | **4** | |

| | | |
|---|---|---|
| 1999 Bangladesh: "success story" in health and family welfare.(Anonymous, 1999) | 1 | |
| (Atkinson & Rubinelli, 2012) | 3 | |
| (Bailey, 2010) | 1 | |
| (J. I. Baker, Griffin, Brauneis, Rue, & McGwin, 2010) | 1 | |
| (T. A. Baker & Wang, 2006) | 1 | |
| Bingley, Thomas, Brown, Reeve, & Payne, 2008) | 1 | |
| (Booth, 2000) | 1 | |
| (Butchko, Tschanz, & Kotsonis, 1994) | 1 | |
| (Blackman et al., 2012) | 3 | |
| (Bovbjerg, 2001) | 1 | |
| (Burger, 2003) | 1 | |
| (Cardey, 2013) | 1 | |
| (Caulfield, Cook-Deegan, Kieff, & Walsh, 2006) | 1 | |
| (Callahan, 2005) | 3 | |
| (Calnan, Wainwright, O'Neill, Winterbottom, & Watkins, 2007) | 1 | |
| (Cameron, 2015) | 3 | |

| | | |
|---|---|---|
| (Cayton, 2004) | 4 | |
| (Charon, 2004) | 3 | |
| (J. Clarke & van Amerom, 2008) | 4 | |
| (Cohen, 2001) | 3 | |
| (Coughlin, 2009) | 4 | |
| (Donovan, 1997) | 1 | |
| (El Feki et al., 2014) | 1 | |
| (Edwards, 2003) | 4 | |
| (Forsyth, 2007) | 1 | |
| (Garzón-Díaz, 2015) | 1 | |
| (Gavin & Rogers, 2006) | 1 | |
| (Gollust et al., 2014) | 1 | |
| (Falk-Rafael & Bradley, 2014) | 3 | |
| (Ficklen, 2009) | 4 | |
| (Gerbensky-Kerber, 2015) | 3 | |
| (Higgs, 2004) | 1 | |
| (Hitchcock, 2013) | 1 | |
| (Horga, Gerdts, & Potts, 2013) | 1 | |
| (Hui & Hilsenrath, 2016) | 1 | |
| (Goldwyn, 2010) | 3 | |
| (Heisey et al., 2011) | 3 | |
| (Heschel, Crowley, & Cohen, 2005) | 1 | |

| | | |
|---|---|---|
| **(D. Hodgetts & Chamberlain, 2003)** | **3** | |
| **(K. Hodgetts, Elshaug, & Hiller, 2012)** | **3** | |
| **(Itkonen & Ream, 2013)** | **1** | |
| **(Jones, 2007)** | **1** | |
| **(Jensen, 2012)** | **1** | |
| **(Adams, 1993)** | **1** | |
| **(Armitage, 1995)** | **3** | |
| **(Arnold, 1997)** | **3** | |
| **(Ball, 2014)** | **3** | |
| **(Bartlett, 2005)** | **3** | |
| **(Bertholds, 2007)** | **4** | |
| **(Bowler, 2009)** | **3** | |
| **(Burda, 1993)** | **3** | |
| **(Caldwell, 2003)** | **3** | |
| **(Casey, 1995)** | **3** | |
| **(Checkland & Harrison, 2010)** | **3** | |
| **(Crow & Berggren, 2014)** | **1** | |
| **(Dickman, 2000)** | **3** | |
| **(Doggett, 2009)** | **1** | |
| **(Epstein, Farina, & Heidt, 2014)** | **3** | |
| **(Bohensky et al., 2010)** | **3** | |

| | | |
|---|---|---|
| **(Giglione, 2009)** | **3** | |
| **(Hayes, 2012)** | **3** | |
| **(Healy, 2003)** | **1** | |
| **(Hoftyzer, 2013)** | | Need to be confirmed |
| **(Lüngen, Schröer-Günther, Passon, Gerber, & Lauterbach, 2009)** | **3** | |
| **Gerber, & Lauterbach, 2009)** | **1** | |
| **(Holtz, 2007)** | **5** | See Marcus 2010 |
| **(Rubotzky, 2000)** | **1** | |
| **(Ryan, 2003)** | **3** | |
| **(Sellman, 1997)** | **3** | |
| **(Pulkingham, Fuller, & Kershaw, 2010)** | **1** | |
| **(Siegal & Ruoff, 2015)** | **1** | |
| **(Silvén Hagström, 2014)** | **3** | |
| **(Sistrom, 2010)** | **1** | |
| **(Slasberg & Beresford, 2016)** | **2** | |
| **(Slater, Hayes, & Chung, 2015)** | **3** | |
| **(Slater, Long, & Ford, 2006)** | **3** | |
| **(Sofer, 2017)** | **1** | |
| **(Spike, 2007)** | **3** | |
| **(Spike, 2011)** | **3** | |

| | | |
|---|---|---|
| **(Spivack, 1994)** | **1** | |
| **(Squier, 1999)** | **3** | |
| **(R. G. Stein, 2010)** | **3** | |
| **(Steiner, 2005)** | **3** | |
| **(Stelfox et al., 2015)** | **1** | |
| **(Stewart, 2007)** | **1** | |
| **(Stilgoe, 2007)** | **1** | |
| **(Swann, 2016)** | **1** | |
| **(Swann, 2016)** | **3** | |
| **(Tickle, 2006)** | **3** | |
| **(Tiefer, 2006)** | **3** | |
| **(Torronen, 2003)** | **3** | |
| **(Trickett, Trimble, & Allen, 2014)** | **1** | |
| **(True & Phipps, 1999)** | **3** | |
| **(Tumber, 2004)** | **2** | |
| **(Tutton, 2009)** | **4** | |
| **(J. E. van Bekkum & S. Hilton, 2013)** | **3** | |
| **(Jennifer E. van Bekkum & Shona Hilton, 2013)** | **3** | |
| **(Vargas et al., 2016)** | **1** | |
| **(von Tigerstrom, 2000)** | **2** | |
| **(H. Wald, A. Richard, V. V. Dickson, & E. Capezuti, 2012)** | **3** | |

| | | |
|---|---|---|
| **(Heidi Wald, Angela Richard, Victoria Vaughan Dickson, & Elizabeth Capezuti, 2012)** | **3** | |
| **(Heidi Wald et al., 2012)** | **3** | |
| **(Wallington, Blake, Taylor-Clark, & Viswanath, 2010)** | **2** | |
| **(Wallington, Blake, Taylor-Clark, Viswanath, et al., 2010)** | **3** | |
| **(Walton, 2004)** | **1** | |
| **(Warner, 2004)** | **3** | |
| **(Weiler-Ravell, Leventhal, Berlowitz, Rishpon, & Chemtob, 2004)** | **1** | |
| **(Weinstock, 2007)** | **1** | |
| **(Whelan, Asbridge, & Haydt, 2011)** | **3** | |
| **(Wiethoff, 2002)** | **2** | |
| **(Wilson-Clay et al., 2005)** | **1** | |
| **(Winett & Lawrence, 2005)** | **3** | |
| **(Wittenstein, 2013)** | **4** | |
| **(Wollaston, 2013)** | **3** | |
| **(A. Wood, 2003)** | **2** | |
| **(W. Wood, 2007)** | **1** | |

| | | |
|---|---|---|
| **(N. Wright, Bartlett, & Callaghan, 2008)** | **1** | |
| **(Wynia, 2007)** | **1** | |
| **(Yankah, 1992)** | **3** | |
| **(Yanovitzky & Stryker, 2001)** | **1** | |
| **(Yapp, Hopcraft, & Parashos, 2011)** | **1** | |
| **(Yaris, Dikici, Akbulut, Yaris, & Sabuncu, 2004)** | **1** | |
| **(Krischke, 2014)** | **3** | |
| **(Kupfer, 2010)** | **1** | |
| **(Kurlantzick, 1977)** | **3** | |
| **(Lamb, 2015)** | **1** | |
| **(Levinson & Richardson, 1976)** | **3** | |
| **(Lewis, Johnson, Farris, & Will, 2004)** | **3** | |
| **(Limb, 2009)** | **1** | |
| **(G. M. Lindsay, 2001)** | **3** | |
| **(S. Lindsay, Selvaraj, Macdonald, & Godden, 2004)** | **3** | |
| **(Greenhalgh, Collard, & Begum, 2005; Lombardo, 2008)** | **1** | |
| **(Long, 1998)** | **1** | |

| | | |
|---|---|---|
| **(Lovgren, Engstrom, & Norberg, 1996)** | **3** | |
| **(Mannion, Freeman, Millar, & Davies, 2016)** | **1** | |
| **(Morrison & Gillett, 2014)** | **1** | |
| **(Neville & Chenery, 1996)** | **3** | |
| **(McFarlane, 1993)** | **1** | |
| **(Penn, 2015)** | **1** | |
| **(C. Smith, 1998)** | **3** | |
| **(Muench, 2010)** | **3** | |
| **(Moodie, 2004)** | **1** | |
| **(McNutt, 2009)** | **3** | |
| **(Pentecost, 2004)** | **1** | |
| **(Van Allen, 1996)** | **1** | |
| **(McGowan, 1990)** | **1** | |
| **(Meyer, 1999)** | **4** | |
| **(Moore, 2012)** | **3** | |
| **(Monteith, 1997)** | **1** | |
| **(Lovgren et al., 1996)** | **3** | |
| **(McGowan, 1990)** | **2** | |
| **(Oman, 2009)** | **2** | |
| **(Strack, 2010)** | **4** | |
| **(Walter, 1983)** | **3** | |
| **(Abou Taam et al., 2014)** | **1** | |

| | | |
|---|---|---|
| **(Kelly, Cooley, & Klinger, 2014)** | **1** | |
| **(Keown & Darzi, 2015)** | **1** | |
| **(Blevins & Werth, 2009)** | **1** | |
| **(LaMontagne, Hunter, Vallance, & Holloway, 2008)** | **1** | |
| **(Lantos, 2012)** | **1** | |
| **(Lasagna, 1980)** | **1** | |
| **(Lee, 2002)** | **1** | |
| **(Leichter, 1994)** | **1** | |
| **(Lemke & Harris-Wai, 2015)** | **1** | |
| **(Leonard, 2006)** | **1** | |
| **(Levi, 2009)** | **1** | |
| **(Levin, 2015)** | **1** | |
| **(Lieberman, 1995)** | **1** | |
| **(R. Lindsay & Graham, 2000)** | **1** | |
| **(Lipschitz, 2010)** | **1** | |
| **(Macdonnell, 2011)** | **1** | |
| **(MacDougall, Riggs, & Lee, 2014)** | **1** | |
| **(Maclennan, Kypri, Room, & Langley, 2013)** | **1** | |
| **(Maier, 2008)** | **1** | |
| **(Malin & Race, 2010)** | **1** | |
| **(Manchikanti, 2006)** | **1** | |
| **(Marmor, 1991)** | **1** | |

| | | |
|---|---|---|
| **(Martins, 2007)** | **1** | |
| **(Mason et al., 2011)** | **1** | |
| **(Matorin & Ringel, 2011)** | **1** | |
| **(M. P. Mattson, 2011)** | **1** | |
| **(Mayes, 2013)** | **1** | |
| **(Mays, 2012)** | **1** | |
| **(McCoyd, 2010)** | **1** | |
| **(Meade, 2010)** | **1** | |
| **(Middleton, 2011)** | **1** | |
| **(D. H. Miller, 1999)** | **1** | |
| **(J. G. Miller, 1997)** | **1** | |
| **(Minot, 2005)** | **1** | |
| **(Monterrosa et al., 2015)** | **1** | |
| **(Morantz & Heymann, 2010)** | **1** | |
| **(Mucciaroni & Killian, 2004)** | **1** | |
| **(Murphy, 2009)** | **1** | |
| **(Nedlund & Nordh, 2015)** | **1** | |
| **(Nettleton & Harding, 1994)** | **1** | |
| **(Newdick & Danbury, 2015)** | **1** | |
| **(Newman & Persson, 2009)** | **1** | |
| **(L. T. Nichols, 1997)** | **1** | |

| | | |
|---|---|---|
| **(Niederdeppe, Kim, Lundell, Fazili, & Frazier, 2012)** | **1** | |
| **(Niederdeppe, Robert, & Kindig, 2011)** | **1** | |
| **(Niederdeppe, Roh, & Dreisbach, 2016)** | **1** | |
| **(Niederdeppe, Heley, & Barry, 2015; Niederdeppe, Roh, & Shapiro, 2015)** | **1** | |
| **(Niederdeppe, Roh, Shapiro, & Kim, 2013)** | **1** | |
| **(Nunes, Juca, & Valentim, 2007)** | **1** | |
| **(Nunn, Dickman, Nattrass, Cornwall, & Gruskin, 2012)** | **1** | |
| **(O'Donovan, 2006)** | **1** | |
| **(Ohemeng, 2013)** | **1** | |
| **(Pachem, 2008)** | **1** | |
| **(Pavlish & Ceronsky, 2009)** | **1** | |
| **(Perez-Botella & Downe, 2006)** | **1** | |
| **(Pestka & Shea, 2016)** | **1** | |
| **(Petrunik & Weisman, 2005)** | **1** | |
| **(Pickering, 2003)** | **1** | |
| **(Ridings, 2013)** | **1** | |
| **(Rodkey, 2016)** | **1** | |
| **(Rochefort, 1998)** | **1** | |

| | | |
|---|---|---|
| **(Rosenberg, 2009)** | **1** | |
| **(Rubin, 2012)** | **1** | |
| **(Sass, 1999)** | **1** | |
| **(Scarparo, Accorssi, & Pizzinato, 2011)** | **1** | |
| **(Scherger, 2014)** | **1** | |
| **(Schmied, Sheehan, & Barclay, 2001)** | **1** | |
| **(Schuftan, 2015)** | **1** | |
| **(Sentell et al., 2016)** | **1** | |
| **(Shanner, 1997)** | **1** | |
| **(Shaw, 2010)** | **1** | |
| **(Sheringham, Baraitser, Simms, Hart, & Raine, 2012)** | **1** | |
| **(Shickle et al., 2014)** | **1** | |
| **(Siciliano, 2012)** | **1** | |
| **(Smidt, 2012)** | **1** | |
| **(Solomon, 1996)** | **1** | |
| **(Stamm, 2007)** | **1** | |
| **(Stanley, Plessis, & Austrin, 2011)** | **1** | |
| **(Starr & Zawacki, 1999)** | **1** | |
| **(Steele, Mebane, Viswanath, & Solomon, 2005)** | **1** | |
| **(P. Stein, 2004)** | **1** | |
| **(P. S. Stein, 1997)** | **1** | |
| **(Stephens, 2012)** | **1** | |

| | | |
|---|---|---|
| **(Stevens & Ritter, 2013)** | **1** | |
| **(Strauss, 2009)** | **1** | |
| **(Sy & Spinelli, 2016)** | **1** | |
| **(Szostak, 2010)** | **1** | |
| **(Tastet, 2010)** | **1** | |
| **(Tolman, Hirschman, & Impett, 2005)** | **1** | |
| **(Trupin, 1993)** | **1** | |
| **(Turner, 1994; Udell & Mehta, 2008; van Amerongen, 1999)** | **1** | |
| **(Vilasboas & Paim, 2008)** | **1** | |
| **(N. Walker, Bryce, & Black, 2007)** | **1** | |
| **(Wallis & Guyer, 2006)** | **1** | |
| **(H. H. Wang, Wang, Zhou, Wang, & Xu, 2013)** | **1** | |
| **(Weaver, 1990)** | **1** | |
| **(Weeks, 2016; Weiss, Watson, & Xuan, 2014)** | **1** | |
| **(Wenger, 2015)** | **1** | |
| **(Wexler, 2012)** | **1** | |
| **(Whitehead et al., 2004)** | **1** | |
| **(Whitworth, 2016)** | **1** | |
| **(Wilson & Johnson, 2003)** | **1** | |
| **(Winchester, 2016)** | **1** | |

| | | |
|---|---|---|
| **(Witson, Fan, Harwood, & Wagenaar, 2004)** | **1** | |
| **(Woeppel, 2013)** | **1** | |
| **(Wohlberg, 2013)** | **1** | |
| **(Wohlers, 2013)** | **1** | |
| **(Woldeguiorguis, 2003)** | **1** | |
| **(Wolfe-Dawson, 2009)** | **1** | |
| **(J. F. Wood, 2005)** | **1** | |
| **(Worthy, 2016)** | **1** | |
| **(Wye et al., 2015)** | **1** | |
| **(Yarborough, 2005)** | **1** | |
| **(Yeboah, 2000)** | **1** | |
| **(Ying, 2016)** | **1** | |
| **(Yoon & Lee, 2008)** | **1** | |
| **(Zebregs, van den Putte, de Graaf, Lammers, & Neijens, 2015)** | **1** | |
| **(Zwald, Jernigan, Payne, & Farris, 2013)** | **1** | |
| **(Mi Kyung & Miyoung, 2013)** | **1** | |
| **(McCullough, 2006)** | **1** | |
| **(Sammy, 2014)** | **1** | |
| **(Wear & Jones, 2010; M. L. White & Fletcher, 1990)** | **1** | |
| **(M. Wright, 2015)** | **1** | |

| | | |
|---|---|---|
| (Willis, DeLeon, Haldane, & Heldring, 2014) | 1 | |
| (Smyth, 1992) | 1 | |
| (Pavlish & Ceronsky, 2009) | 1 | |
| (S. L. Nichols, Friedland, Rojas, Cho, & Shah, 2006) | 1 | |
| (Rifkin, 2017) | | |
| (R. Paterson, 2015) | 3 | |
| (C. A. Walker, Cohen, & Jenkins, 2016) | 1 | |
| (Winakur, 2010) | 3 | |
| (Len-Rios, Onyebadi, Qiu, & Phillips) | 1 | |
| (MacGregor, 2013b) | 1 | |
| (O'Connor, 2005) | 3 | |
| (Oliveira Friestino, 2016) | 1 | |
| (Roberts et al., 2012) | 1 | |
| (Sherrod, 2006) | 1 | |
| (C. J. Smith, 2007) | 1 | |
| (Stockton, 1966) | 1 | |
| (Tiffany, 1891c) | 1 | |
| (Tiffany, 1891e) | 1 | |
| (Tiffany, 1891d) | 1 | |
| (Tiffany, 1891a) | 1 | |
| (Tumber, 2004) | 2 | |

| | | |
|---|---|---|
| **(J. Y. Wang, Thistlethwaite, & Ross, 2015)** | **1** | |
| **(A. Watkins, 2011)** | **3** | |
| **(J. Watkins et al., 2011)** | **3** | |
| **(Miser, 2005)** | **1** | |
| **(Short, 2006)** | **1** | |
| **(Wylie & Nebauer, 2011)** | **2** | |
| **(Poltorak, Leach, Fairhead, & Cassell, 2005)** | **3** | |
| **(Hall, 2002)** | **3** | |
| **(K. C. Smith, Terry-McElrath, Wakefield, & Durrant, 2005)** | **1** | |
| **(Sudhanshu, Pankaj, Sorabh, & Nidhi, 2014)** | **2** | |
| **(DiCenso et al., 2012)** | **1** | |
| **(Burton, 2003)** | **1** | |
| **(Grant, Anderson, & Machila, 2015)** | **3** | |
| **(Laite, 2014)** | **1** | |
| **(Lockey, Maidment, & O'Brien, 2011)** | **4** | |
| **(Macfarlane, Exworthy, Wilmott, & Greenhalgh, 2011)** | **3** | |
| **(Mears, 2003)** | **3** | |
| **(Payne et al., 2013)** | **3** | |
| **(Silver, 2001)** | **3** | |

| | | |
|---|---|---|
| **(D. C. Smith, 2014)** | **2** | |
| **(M. Smith, 1993)** | **3** | |
| **(A. Smith, 2008)** | **3** | |
| **(J. A. Smith, 2014)** | **1** | |
| **(R. F. Smith, 2015)** | **3** | |
| **(Tiffany, 1891b)** | **3** | |
| **(Weishaar, Collin, & Amos, 2016)** | **1** | |
| **(Weishaar, Dorfman, et al., 2016)** | **3** | |
| **(England, 2007)** | **1** | |
| **(Hoftyzer, 2013)** | | |
| **(K. Mullen, 1989)** | **1** | |
| **(Afeef & Alkhoulli, 2010)** | **3** | |
| **(M. Anderson & Asnani, 2016)** | **3** | |
| **(Anonymous, 1997a)** | **1** | |
| **(Anonymous, 1997b)** | **1** | |
| **(Anonymous, 1998b)** | **1** | |
| **(Anonymous, 1998a)** | **3** | |
| **(Anonymous, 2000c)** | **3** | |
| **(Anonymous, 2000b)** | **3** | |
| **(Anonymous, 2000a)** | **3** | |
| **(Anonymous, 2002)** | **1** | |
| **(Anonymous, 2003)** | **1** | |
| **(Anonymous, 2009)** | **3** | |

| | | |
|---|---|---|
| **("Conversation with Martin Jarvis," 2011)** | **4** | |
| **(Braun, 2007)** | **3** | |
| **(Cambell, 2003)** | **4** | **Magazine** |
| **(Dawson, Farmer, & Thomson, 2011)** | **3** | |
| **(Gardner, 2009)** | **3** | **Narrative promoted advocacy (without being linked to policy cycle)** |
| **(Greenhalgh et al., 2005)** | **3** | |
| **(Holtz, 2007)** | **4** | |
| **(Lafferton, 2006)** | **1** | **Narrative not planned or deliberate** |
| **(Lagerwey, 1999)** | **1** | **Narrative not planned or deliberate** |
| **(Gail M. Lindsay, Mior, Côté, Carroll, & Shearer, 2016)** | **3** | |
| **(Anne Manchester, 2016)** | **3** | |
| **(McLntyre, 2013)** | | **Intervention more related to media** |
| **(Z. F. Meisel & J. Karlawish, 2011)** | **4** | **Commentary** |
| **(Mitchell, 2013)** | **4** | **New article** |
| **(Morgan, 2005)** | **3** | |
| **(Norko, 2010)** | **4** | **Commentary** |
| **(Novak, 1998)** | **1** | |
| **(Ofuji, 2007)** | **2** | |

| | | |
|---|---|---|
| **(O'Neill, 2007)** | **2** | |
| **(B. Paterson, 2006)** | **1** | **Intervention more related to media** |
| **(Peabody, Hesketh, & Steinberg, 1996)** | **1** | |
| **(Robertson, Walkom, Bevan, & Newby, 2013)** | **1** | **Intervention more related to media** |
| **(Russell, Voas, Dejong, & Chaloupka, 1995)** | **1** | |
| **(Sherer, 2008)** | **1** | **Narrative not planned or deliberate** |
| **(Solleder, 1981)** | **1** | |
| **(Speaks, 1996)** | **3** | |
| **(Spear, 2006)** | **1** | **Narrative not planned or deliberate** |
| **(Thomas, 2017)** | **1** | |
| **(Vega, 2011)** | **3** | |
| **(von Klan, 2015)** | **2** | |
| **(Wagner, 2010)** | **3** | **General advocacy; not linked to policy changes** |
| **(C. White, 2006)** | **2** | |
| **(Zwelling, 2001)** | **4** | |
| **(Zwelling, 2002)** | **4** | |
| **("Power and Effectiveness of Nurses," 2016)** | **1** | |
| **(Bodai, 2001)** | **3** | |
| **(Hill, 2009)** | **3** | |

| | | |
|---|---|---|
| **(Illman, 2000)** | **3** | |
| **(Interlandi & Springen, 2008)** | **1** | **Narrative not planned or deliberate** |
| **(Lewis et al., 2004)** | **1** | |
| **("Taking their side: the power of storytelling," 2013)** | **3** | |
| **(Angelelli et al., 2002)** | **1** | |
| **(Belliveau, 2011)** | **2** | |
| **(Black, 2011)** | **1** | |
| **(Bright, Marsh, Smith, & Bishop, 2008)** | **1** | **Media-related** |
| **(C. E. Clarke, Niederdeppe, & Lundell, 2012)** | **3** | |
| **(Cullinane, 2005)** | **3** | |
| **(Davis-Berman, 2011)** | **3** | |
| **(Evans, 2010)** | **3** | **General discussion with no link to specific policy change** |
| **(Elwood, 2005)** | **1** | |
| **(Goodwin et al., 2013)** | **1** | |
| **(Harbridge, 2012)** | **3** | |
| **(Hooker & Chapman, 2006)** | **3** | **General discussion with no link to specific policy change** |
| **(Hsu & McCormack, 2012)** | **3** | |
| **(Kendall, 2013 )** | **3** | |
| **(Kovacs Burns, 2008)** | **1** | |

| | | |
|---|---|---|
| **(Krinks, Kendall, Whitty, & Scuffham, 2016)** | **1** | |
| **(Lokugamage & Pathberiya, 2017)** | **3** | |
| **(Lonne & Gillespie, 2014)** | **1** | **Media-related** |
| **(Lublinski et al., 2016)** | **1** | **Investigative journalism** |
| **(MacGregor, 2013a)** | **1** | |
| **(A. Manchester & O'Connor, 2008)** | **3** | |
| **(McConnell-Henry, 2012)** | **3** | |
| **(McDonough, 2001)** | **4** | |
| **(McGee, 2005)** | **3** | |
| **(Zachary F. Meisel & Jason Karlawish, 2011)** | **4** | |
| **(E. A. Miller, Tyler, Rozanova, & Mor, 2012)** | **1** | **Media-related** |
| **(Niechajev & Frame, 2012)** | **1** | **Narrative not planned or deliberate** |
| **(Niederdeppe, Shapiro, & Porticella, 2010)** | **3** | |
| **(Pandya, 2012)** | **3** | |
| **(Pullman, Zarzeczny, & Picard, 2013)** | **1** | |
| **(Sinclair, Makin, Tang, Brozek, & Rock, 2014)** | **5** | See MacKenzie, 2008 |
| **(Spear, 2006)** | **1** | |
| **(Stewart, 2004)** | **3** | |
| **(Troy & Kietzman, 2016)** | **3** | |

| | | |
|---|---|---|
| **(Usdin, 2009)** | **1** | |
| **(Williams, Powell, Hoskins, & Neville, 2008)** | **1** | |
| **(Wynn, 2010)** | **2** | |
| **(Mullan, Ficklen, & Rubin, 2006)** | **4** | |
| **(Tanne, 2006)** | **3** | |
| **(Yoshikawa & Olazagasti, 2011)** | **3** | |
| **(Penn, 2014)** | **3** | |
| **(Martinez, 2005)** | **3** | |
| **(M. Mattson, 2010)** | **1** | **More focused on advocacy (narrative was not part of intervention)** |
| **(Murray, 2001)** | **4** | |
| **(M. L. Smith & Flamm, 2011)** | **3** | |
| **(Brownson, Chriqui, & Stamatakis, 2009)** | **3** | |
| **(Brownson et al., 2007)** | **1** | |
| **(Brownson, Dodson, Kerner, & Moreland-Russell, 2016)** | **3** | |
| **(Brownson et al., 2011)** | **3** | |
| **(Purtle, Dodson, & Brownson, 2016)** | **3** | |
| **(Price, 2011)** | **3** | |
| **(E. Mullen, 1999)** | **1** | **More focused on advocacy (narrative** |

| | | |
|---|---|---|
| | | **was not part of intervention)** |

## References for excluded studies


Abeysinghe, S., & White, K. (2011). The avian influenza pandemic: Discourses of risk, contagion and preparation in Australia. *Health, Risk & Society, 13*(4), 311-326. doi:10.1080/13698575.2011.575457

Abou Taam, M., Rossard, C., Cantaloube, L., Bouscaren, N., Roche, G., Pochard, L., . . . Bagheri, H. (2014). Analysis of patients' narratives posted on social media websites on benfluorex's (Mediator ) withdrawal in France. *Journal of Clinical Pharmacy & Therapeutics, 39*(1), 53-55. doi:https://dx.doi.org/10.1111/jcpt.12103

Adams, F. B. (1993). Socialized lumber. *Journal - South Carolina Medical Association, 89*(12), 596-597.

Afeef, M., & Alkhoulli, L. (2010). Surprised by joy: a journey through suffering. *Asian Pacific Journal of Cancer Prevention: Apjcp, 11 Suppl 1*, 125-126.

Akintola, O., Lavis, J. N., & Hoskins, R. (2015). Print media coverage of primary healthcare and related research evidence in South Africa. *Health Research Policy & Systems, 13*, 68. doi:https://dx.doi.org/10.1186/s12961-015-0051-6

Allison, J. J., Nguyen, H. L., Ha, D. A., Chiriboga, G., Ly, H. N., Tran, H. T., . . . Goldberg, R. J. (2016). Culturally adaptive storytelling method to improve hypertension control in Vietnam - "We talk about our hypertension": study protocol for a feasibility cluster-randomized controlled trial. *Trials [Electronic Resource], 17*, 26. doi:https://dx.doi.org/10.1186/s13063-015-1147-6

Anaf, J., Newman, L., Baum, F., Ziersch, A., & Jolley, G. (2013). Policy environments and job loss: Lived experience of retrenched Australian automotive workers. *Critical Social Policy, 33*(2), 325-347. doi:10.1177/0261018312457858

Andersen, A. E. (2013). It's War Over There. *Eating Disorders, 21*(2), 181-183. doi:10.1080/10640266.2013.761093

Anderson, K. A. (2011). *The impact that leadership practices of the nurse manager and nursing practice environments have on job satisfaction of registered nurses in two urban teaching hospitals.* (Ph.D.), University of Maryland Eastern Shore. Retrieved from http://search.ebscohost.com/login.aspx?direct=true&db=rzh&AN=109863934&site=ehost-live Available from EBSCOhost rzh database.

Anderson, M., & Asnani, M. (2016). 'The white blood cell always eat the red': how Jamaicans with sickle cell disease understand their illness. *Ethn Health, 21*(2), 103-117.

Angelelli, J., Gifford, D., Intrator, O., Gozalo, P., Laliberte, L., & Mor, V. (2002). Access to postacute nursing home care before and after the BBA. Balanced Budget Act. *Health Affairs, 21*(5), 254-264.

Anonymous. (1997a). Genetic testing for cystic fibrosis. *NIH Consensus Statement, 15*(4), 1-37.

Anonymous. (1997b). Interventions to prevent HIV risk behaviors. *NIH Consensus Statement, 15*(2), 1-41.

Anonymous. (1998a). Effective medical treatment of opiate addiction. National Consensus Development Panel on Effective Medical Treatment of Opiate Addiction. *JAMA, 280*(22), 1936-1943.

Anonymous. (1998b). Rehabilitation of persons with traumatic brain injury. *NIH Consensus Statement, 16*(1), 1-41.

Anonymous. (1999). Bangladesh: "success story" in health and family welfare. *Cirdap Development Digest (Cdd)*(77), 20.

Anonymous. (2000a). Adjuvant therapy for breast cancer. *NIH Consensus Statement, 17*(4), 1-35.

Anonymous. (2000b). Antenatal corticosteroids revisited: repeat courses. *NIH Consensus Statement, 17*(2), 1-18.

Anonymous. (2000c). Phenylketonuria (PKU): screening and management. *NIH Consensus Statement, 17*(3), 1-33.

Anonymous. (2002). NIH State-of-the-Science Statement on symptom management in cancer: pain, depression, and fatigue. *NIH Consensus & State-of-the-Science Statements, 19*(4), 1-29.

Anonymous. (2003). NIH Consensus Statement on total knee replacement. *NIH Consensus & State-of-the-Science Statements, 20*(1), 1-34.
Anonymous. (2009). Naked in front of the eyedoctor: life through the eyes of a glaucoma patient. *Bulletin de la Societe Belge d Ophtalmologie*(312), 55-58; discussion 53.
Armitage, P. (1995). Changes in long-term mental health nursing. *Nursing Standard, 9*(38), 28-30.
Arnold, L. M. (1997). In your best interest. Lynda's story: campaigning for health care worker safety... this article was excerpted from Asepsis, with permission. *Today's Surgical Nurse, 19*(1), 48-49.
Atkinson, S., & Rubinelli, S. (2012). Narrative in cancer research and policy: voice, knowledge and context. *Critical Reviews in Oncology-Hematology, 84 Suppl 2*, S11-16. doi:https://dx.doi.org/10.1016/S1040-8428(13)70004-0
Bailey, J. (2010). An assessment of the use of chimpanzees in hepatitis C research past, present and future: 1. Validity of the chimpanzee model. *ATLA-Alternatives to Laboratory Animals, 38*(5), 387-418.
Baker, J. I., Griffin, R., Brauneis, P. F., Rue, L. W., 3rd, & McGwin, G., Jr. (2010). A comparison of wakeboard-, water skiing-, and tubing-related injuries in the United States, 2000-2007. *Journal of Sports Science & Medicine, 9*(1), 92-97.
Baker, T. A., & Wang, C. C. (2006). Photovoice: use of a participatory action research method to explore the chronic pain experience in older adults. *Qualitative Health Research, 16*(10), 1405-1413. doi:https://dx.doi.org/10.1177/1049732306294118
Ball, D. A. (2014). *A phenomenological study of the nurse leader: before, during, and after merger.* (Ed.D.), Bowling Green State University. Retrieved from http://search.ebscohost.com/login.aspx?direct=true&db=rzh&AN=109786403&site=ehost-live Available from EBSCOhost rzh database.
Bartlett, A. (2005). Scripts and meanings for breastfeeding in popular culture. *Birth Issues, 14*(1), 7-12.
Belliveau, M. (2011). Gendered matters: undocumented mexican mothers in the current policy context. *Affilia: Journal of Women & Social Work, 26*(1), 32-46. doi:10.1177/0886109910392534
Bertholds, E. (2007). [Anecdotes not sufficient as arguments in the debate on profits in health care]. *Lakartidningen, 104*(35), 2450.
Black, L. M. (2011). Tragedy into policy: a quantitative study of nurses' attitudes toward patient advocacy activities. *American Journal of Nursing, 111*(6), 26-35; quiz 36-27. doi:https://dx.doi.org/10.1097/01.NAJ.0000398537.06542.c0
Blackman, T., Harrington, B., Elliott, E., Greene, A., Hunter, D. J., Marks, L., . . . Williams, G. (2012). Framing health inequalities for local intervention: comparative case studies. *Sociology of Health & Illness, 34*(1), 49-63. doi:https://dx.doi.org/10.1111/j.1467-9566.2011.01362.x
Blevins, D., & Werth, J. L., Jr. (2009). Introduction. In J. L. Werth, Jr., D. Blevins, J. L. Werth, Jr., & D. Blevins (Eds.), *Decision making near the end-of-life: Issues, developments, and future directions.* (pp. 1-7). New York, NY, US: Routledge/Taylor & Francis Group.
Bodai, E. (2001). In their own words. One man's mission against cancer. *Bulletin of the American College of Surgeons, 86*(2), 28-30.
Bohensky, M. A., Jolley, D., Sundararajan, V., Evans, S., Pilcher, D. V., Scott, I., & Brand, C. A. (2010). Data linkage: a powerful research tool with potential problems. *BMC Health Services Research, 10*, 346. doi:https://dx.doi.org/10.1186/1472-6963-10-346
Booth, K. M. (2000). 'JUST TESTING'. *Gender & Society, 14*(5), 644.
Bovbjerg, R. R. (2001). Medical safety: from stories to policy. *Health Affairs, 20*(2), 241-242.
Bowler, M. (2009). Exploring patients' experiences of a community matron service using storybooks. *Nursing Times, 105*(24), 19-21.

Braun, J. A. (2007). The imperatives of narrative: health interest groups and morality in network news. *American Journal of Bioethics, 7*(8), 6-14. doi:https://dx.doi.org/10.1080/15265160701462244
Bright, S. J., Marsh, A., Smith, L. M., & Bishop, B. (2008). What can we say about substance use? Dominant discourses and narratives emergent from Australian media. *Addiction Research & Theory, 16*(2), 135-148.
Brownson, R. C., Chriqui, J. F., & Stamatakis, K. A. (2009). Understanding evidence-based public health policy. *American Journal of Public Health, 99*(9), 1576-1583. doi:https://dx.doi.org/10.2105/AJPH.2008.156224
Brownson, R. C., Diem, G., Grabauskas, V., Legetic, B., Potemkina, R., Shatchkute, A., . . . Waller, M. (2007). Training practitioners in evidence-based chronic disease prevention for global health. *Promotion et Education, 14*(3), 159-163.
Brownson, R. C., Dodson, E. A., Kerner, J. F., & Moreland-Russell, S. (2016). Framing research for state policymakers who place a priority on cancer. *Cancer Causes & Control, 27*(8), 1035-1041. doi:https://dx.doi.org/10.1007/s10552-016-0771-0
Brownson, R. C., Dodson, E. A., Stamatakis, K. A., Casey, C. M., Elliott, M. B., Luke, D. A., . . . Kreuter, M. W. (2011). Communicating evidence-based information on cancer prevention to state-level policy makers. *Journal of the National Cancer Institute, 103*(4), 306-316. doi:https://dx.doi.org/10.1093/jnci/djq529
Burda, D. (1993). Reform's impact on people lacking in news stories--study. *Modern Healthcare, 23*(41), 30.
Burger, J. (2003). Consistency among methods of assessing concerns about the Los Alamos National Laboratory. *J Toxicol Environ Health A, 66*(2), 199-210.
Burton, L. R. (2003). The Mersey Basin: an historical assessment of water quality from an anecdotal perspective. *Science of the Total Environment, 314-316*, 53-66.
Butchko, H. H., Tschanz, C., & Kotsonis, F. N. (1994). Postmarketing surveillance of food additives. *Regulatory Toxicology & Pharmacology, 20*(1 Pt 1), 105-118. doi:https://dx.doi.org/10.1006/rtph.1994.1039
Caldwell, P. H. (2003). *Lives of rural women after myocardial infarction: A critical ethnography.* (Ph.D.), McMaster University (Canada). Retrieved from http://search.ebscohost.com/login.aspx?direct=true&db=rzh&AN=109844088&site=ehost-live Available from EBSCOhost rzh database.
Callahan, D. (2005). Before he wakes. *Hastings Center Report, 35*(4), 16-16.
Calnan, M., Wainwright, D., O'Neill, C., Winterbottom, A., & Watkins, C. (2007). Illness action rediscovered: A case study of upper limb pain. *Sociology of Health & Illness, 29*(3), 321-346. doi:10.1111/j.1467-9566.2007.00543.x
Cambell, S. C. (2003). Sharing stories moves Congress to act. *Exceptional Parent, 33*(7), 50-55.
Cameron, L. (2015). The thing is, we all have stories don't we? *Tizard Learning Disability Review, 20*(1), 37-40. doi:10.1108/TLDR-09-2014-0031
Cardey, S. P. (2013). Review of Development and public health communication. *Mass Communication & Society, 16*(2), 295-298. doi:10.1080/15205436.2012.760739
Casey, N. (1995). Editorial... much more can be uncovered through simply recounting an experience. *Nursing Standard, 9*(15), 3-3.
Caulfield, T., Cook-Deegan, R. M., Kieff, F. S., & Walsh, J. P. (2006). Evidence and anecdotes: an analysis of human gene patenting controversies. *Nature Biotechnology, 24*(9), 1091-1094. doi:https://dx.doi.org/10.1038/nbt0906-1091
Cayton, H. (2004). Telling stories: choices and challenges on the journey of dementia. *Dementia (14713012), 3*(1), 9-17.
Charon, R. (2004). Narrative and medicine. *New England Journal of Medicine, 350*(9), 862-864.

Checkland, K., & Harrison, S. (2010). The impact of the Quality and Outcomes Framework on practice organisation and service delivery: summary of evidence from two qualitative studies. *Quality in Primary Care, 18*(2), 139-146.
Clarke, C. E., Niederdeppe, J., & Lundell, H. C. (2012). Narratives and images used by public communication campaigns addressing social determinants of health and health disparities. *International Journal of Environmental Research & Public Health [Electronic Resource], 9*(12), 4254-4277.
Clarke, J., & van Amerom, G. (2008). Mass print media depictions of cancer and heart disease: community versus individualistic perspectives? *Health & Social Care in the Community, 16*(1), 96-103. doi:https://dx.doi.org/10.1111/j.1365-2524.2007.00731.x
Cohen, J. S. (2001). EMTALA: is the cure worse than the disease? *Journal of Emergency Medicine, 21*(4), 439-441.
Conversation with Martin Jarvis. (2011). *Addiction, 106*(2), 260-266. doi:10.1111/j.1360-0443.2010.03093.x
Coughlin, C. N. (2009). Reviews in health law. An examination of the power of narrative: a review of Health law and bioethics: cases in context. *Journal of Law, Medicine & Ethics, 37*(4), 857-859. doi:10.1111/j.1748-720X.2009.00458.x
Crow, D. A., & Berggren, J. (2014). Using the narrative policy framework to understand stakeholder strategy and effectiveness: A multi-case analysis. *The science of stories: Applications of the Narrative Policy Framework in public policy analysis*, 131-156.
Cullinane, J. (2005). Tainted blood and vengeful spirits: the legacy of Japan's yakugai eizu (AIDS) trial. *Culture, Medicine & Psychiatry, 29*(1), 5-31.
Davis-Berman, J. (2011). Older men in the homeless shelter: in-depth conversations lead to practice implications. *Journal of Gerontological Social Work, 54*(5), 456-474. doi:https://dx.doi.org/10.1080/01634372.2011.570863
Dawson, P., Farmer, J., & Thomson, E. (2011). The power of stories to persuade: The storying of midwives and the financial narratives of central policy makers. *Journal of Management & Organization, 17*(2), 146-164. doi:10.5172/jmo.2011.17.2.146
DiCenso, A., Housden, L., Heale, R., Carter, N., Canitz, B., MacDonald-Rencz, S., & Buckley, C. R. (2012). Incorporating a health policy practicum in a graduate training program to prepare advanced practice nursing health services researchers. *Policy, Politics, & Nursing Practice, 13*(4), 224-233. doi:https://dx.doi.org/10.1177/1527154413484067
Dickman, R. L. (2000). Bending the rules to get a medication. *American Family Physician, 61*(5), 1563-1564.
Doggett, G. (2009). Making 'meaningful use' meaningful: the importance of the narrative for quality of care and EHR adoption. *Health Data Matrix, 28*(4), 34-36.
Donovan, M. C. (1997). The problem with making AIDS comfortable: federal policy making and the rhetoric of innocence. *Journal of Homosexuality, 32*(3-4), 115-144. doi:https://dx.doi.org/10.1300/J082v32n03_05
Edwards, G. (2003). Cannabis and the evidence that led to its international control: A cautionary tale. *Addiction, 98*(2), 141-141. doi:10.1046/j.1360-0443.2003.00325.x
El Feki, S., Avafia, T., Fidalgo, T. M., Divan, V., Chauvel, C., Dhaliwal, M., & Cortez, C. (2014). The Global Commission on HIV and the Law: recommendations for legal reform to promote sexual and reproductive health and rights. *Reproductive Health Matters, 22*(44), 125-136. doi:https://dx.doi.org/10.1016/S0968-8080(14)44807-9
Elwood, T. W. (2005). The influence of words as determinants of U.S. international and domestic health policy--part I. *International Quarterly of Community Health Education, 24*(2), 99-109. doi:https://dx.doi.org/10.2190/A0PL-TEWP-DWU4-D54J
England, S. K. (2007). From laboratory to legislation. *Physiologist, 50*(3), 91.
Epstein, D., Farina, C., & Heidt, J. (2014). The value of words: Narrative as evidence in policy making. *Evidence and Policy, 10*(2), 243-258. doi:10.1332/174426514X13990325021128

Evans, G. (2010). Patient stories: MRSA is gut-wrenchingly humanized, and lawmakers are listening. *Hospital Infection Control & Prevention, 37*(11), 121-125.
Falk-Rafael, A., & Bradley, P. A. (2014). "Towards justice in health": an exemplar of speaking truth to power. *Advances in Nursing Science, 37*(3), 224-234. doi:https://dx.doi.org/10.1097/ANS.0000000000000034
Ficklen, E. (2009). Narrative Matters turns ten. *Health Affairs, 28*(4), 1161-1162. doi:10.1377/hlthaff.28.4.1161
Forsyth, S. (2007). Telling stories: nurses, politics and Aboriginal Australians, circa 1900-1980s. *Contemporary Nurse, 24*(1), 33-44. doi:https://dx.doi.org/10.5555/conu.2007.24.1.33
Gardner, K. E. (2009). Disruption and cancer narratives: from awareness to advocacy. *Literature & Medicine, 28*(2), 333-350.
Garzón-Díaz, K. (2015). De la inclusión a la convivencia: las narrativas de niños y niñas en diálogo con políticas públicas en discapacidad. [From the inclusion to the coexistence: The narratives of children in dialogue with public policies on disability]. *Rev Fac Med Univ Nac Colomb, 63*(supl.1), 67-74.
Gavin, M., & Rogers, A. (2006). Narratives of suicide in psychological autopsy: bringing lay knowledge back in. *Journal of Mental Health, 15*(2), 135-144.
Gerbensky-Kerber, A. (2015). What we (don't) talk about when we talk about breasts. *Health Communication, 30*(6), 624-626. doi:10.1080/10410236.2014.914122
Giglione, L. (2009). Sharing our thoughts. Patient stories -- the key to successful Medicare legislation. *Infusion, 15*(2), 46-47.
Goldwyn, R. M. (2010). Laws of plastic surgery. *Plastic & Reconstructive Surgery, 126*(2), 700.
Gollust, S. E., Kite, H. A., Benning, S. J., Callanan, R. A., Weisman, S. R., & Nanney, M. S. (2014). Use of research evidence in state policymaking for childhood obesity prevention in Minnesota. *American Journal of Public Health, 104*(10), 1894-1900. doi:https://dx.doi.org/10.2105/AJPH.2014.302137
Goodwin, D. M., Cummins, S., Sautkina, E., Ogilvie, D., Petticrew, M., Jones, A., . . . White, M. (2013). The role and status of evidence and innovation in the healthy towns programme in England: a qualitative stakeholder interview study. *Journal of Epidemiology & Community Health, 67*(1), 106-112. doi:https://dx.doi.org/10.1136/jech-2012-201481
Grant, C., Anderson, N., & Machila, N. (2015). Stakeholder Narratives on Trypanosomiasis, Their Effect on Policy and the Scope for One Health. *PLoS Neglected Tropical Diseases [electronic resource], 9*(12), e0004241. doi:https://dx.doi.org/10.1371/journal.pntd.0004241
Greenhalgh, T., Collard, A., & Begum, N. (2005). Sharing stories: complex intervention for diabetes education in minority ethnic groups who do not speak English. *BMJ, 330*(7492), 628. doi:https://dx.doi.org/10.1136/bmj.330.7492.628
Hall, K. (2002). Medical decision-making: an argument for narrative and metaphor. *Theoretical Medicine & Bioethics, 23*(1), 55-73.
Harbridge, E. (2012). Taking on the council. *Community Living, 25*(3), 18-19.
Hayes, K. A. (2012). *Barriers to Hospice Enrollment for Patients with Advanced Cancer in an Academic Cancer Center.* (D.N.P.), University of Virginia. Retrieved from http://search.ebscohost.com/login.aspx?direct=true&db=rzh&AN=109858597&site=ehost-live Available from EBSCOhost rzh database.
Healy, J. (2003). Analysing health care systems performance: the story behind the statistics. *Australian & New Zealand Journal of Public Health, 27*(6), 642-644.
Heisey, R., Clemons, M., Granek, L., Fergus, K., Hum, S., Lord, B., . . . Fitzgerald, B. (2011). Health care strategies to promote earlier presentation of symptomatic breast cancer: perspectives of women and family physicians. *Current Oncology, 18*(5), e227-237.
Heschel, R. T., Crowley, A. A., & Cohen, S. S. (2005). State policies regarding nursing delegation and medication administration in child care settings: a case study. *Policy, Politics, & Nursing Practice, 6*(2), 86-98. doi:https://dx.doi.org/10.1177/1527154405275884

Higgs, R. (2004). The contribution of narrative ethics to issues of capacity in psychiatry. *Health Care Analysis, 12*(4), 307-272. doi:10.1007/s10728-004-6638-7

Hill, S. L. (2009). Helping policymakers see through the eyes of the infant. *Zero to Three, 29*(6), 4-7.

Hitchcock, J. (2013). Publisher's Note relating to 'Letter from New Zealand: Individualising Breast Feeding Policies' [Journal of Neonatal Nursing 18(3) (2012) 88-89]. *Journal of Neonatal Nursing, 19*(1), 25-25.

Hodgetts, D., & Chamberlain, K. (2003). Narrativity and the mediation of health reform agendas. *Sociology of Health & Illness, 25*(6), 553-570.

Hodgetts, K., Elshaug, A. G., & Hiller, J. E. (2012). What counts and how to count it: physicians' constructions of evidence in a disinvestment context. *Social Science & Medicine, 75*(12), 2191-2199. doi:https://dx.doi.org/10.1016/j.socscimed.2012.08.016

Hoftyzer, M. K. (2013). *Narrative, ethos, and artificial fluoridation: The 'storying' of a public health policy.* (74), ProQuest Information & Learning, US. Retrieved from http://search.ebscohost.com/login.aspx?direct=true&db=psyh&AN=2013-99211-132&site=ehost-live Available from EBSCOhost psyh database.

Holtz, A. (2007). Scriptdoctor: medicine in the media. How an ER storyline helped pass the Patient Navigator Act. *Oncology Times, 29*(4), 24-28.

Hooker, C., & Chapman, S. (2006). Deliberately personal: tobacco control debates and deliberative democracy in New South Wales. *Critical Public Health, 16*(1), 35-46.

Horga, M., Gerdts, C., & Potts, M. (2013). The remarkable story of Romanian women's struggle to manage their fertility. *Journal of Family Planning & Reproductive Health Care, 39*(1), 2-4. doi:https://dx.doi.org/10.1136/jfprhc-2012-100498

Hsu, M. Y., & McCormack, B. (2012). Using narrative inquiry with older people to inform practice and service developments. *Journal of Clinical Nursing, 21*(5-6), 841-849. doi:https://dx.doi.org/10.1111/j.1365-2702.2011.03851.x

Hui, L., & Hilsenrath, P. (2016). Organization and Finance of China's Health Sector: Historical Antecedents for Macroeconomic Structural Adjustment. *Inquiry (00469580), 53*, 1-8. doi:10.1177/0046958015620175

Illman, J. (2000). Patients' voices grow louder in Great Britain. *Journal of the National Cancer Institute, 92*(17), 1373-1375.

Interlandi, J., & Springen, K. (2008). The woman who died in the waiting room. *Newsweek, 152*(3), 48-50.

Itkonen, T., & Ream, R. (2013). Autism advocacy: A network striving for equity. *Peabody Journal of Education, 88*(1), 48-59. doi:10.1080/0161956X.2013.752631

Jensen, E. (2012). Mediating subpolitics in US and UK science news. *Public Understanding of Science, 21*(1), 68-83.

Jones, R. (2007). We've got a story to tell. *Community Care*(1682), 6-6.

Kelly, R. P., Cooley, S. R., & Klinger, T. (2014). Narratives can motivate environmental action: the Whiskey Creek ocean acidification story. *Ambio, 43*(5), 592-599. doi:https://dx.doi.org/10.1007/s13280-013-0442-2

Kendall, J. (2013 ). Taking their side: The power of storytelling. *The Journal of Dementia Care, 21*(1).

Keown, O. P., & Darzi, A. (2015). The quality narrative in health care. *Lancet, 385*(9976), 1367-1368. doi:https://dx.doi.org/10.1016/S0140-6736(15)60639-0

Kovacs Burns, K. (2008). Canadian patient safety champions: collaborating on improving patient safety. *Healthcare Quarterly, 11*(3 Spec No.), 95-100.

Krinks, R., Kendall, E., Whitty, J. A., & Scuffham, P. A. (2016). Do consumer voices in health-care citizens' juries matter? *Health Expectations, 19*(5), 1015-1022. doi:https://dx.doi.org/10.1111/hex.12397

Krischke, M. M. (2014). Her Family's Story: Nurse-Author Advocates for Patient-centered Changes. *NurseZone Newsletter*, 1-1.

Kupfer, T. (2010). CMFE – Community Media Forum Europe. *Telematics & Informatics, 27*(2), 187-192. doi:10.1016/j.tele.2009.06.009
Kurlantzick, L. (1977). 'Medical Story': some (mis?) conceptions about law. *Medicine, Science & the Law, 17*(1), 71-73.
Lafferton, E. (2006). Death by hypnosis: an 1894 Hungarian case and its European reverberations. *Endeavour, 30*(2), 65-70. doi:https://dx.doi.org/10.1016/j.endeavour.2006.04.005
Lagerwey, M. D. (1999). Nursing, social contexts, and ideologies in the early United States birth control movement. *Nursing Inquiry, 6*(4), 250-258.
Laite, J. (2014). Justifiable Sensationalism. *Media History, 20*(2), 126-145. doi:10.1080/13688804.2014.898896
Lamb, M. E. (2015). Toward developmentally aware practices in the legal system: Progress, challenge, and promise. *Am Psychol, 70*(8), 686-693.
LaMontagne, A. D., Hunter, C. E., Vallance, D., & Holloway, A. J. (2008). Asbestos disease in Australia: looking forward and looking back. *New Solutions, 18*(3), 361-373. doi:https://dx.doi.org/10.2190/NS.18.3.j
Lantos, J. D. (2012). Ethics for the pediatrician: the evolving ethics of cochlear implants in children. *Pediatrics in Review, 33*(7), 323-326. doi:https://dx.doi.org/10.1542/pir.33-7-323
Lasagna, L. (1980). The Halcion story: trial by media. *Lancet, 1*(8172), 815-816.
Lee, S. (2002). Socio-cultural and global health perspectives for the development of future psychiatric diagnostic systems. *Psychopathology, 35*(2-3), 152-157. doi:https://dx.doi.org/65136
Leichter, H. M. (1994). Health care reform in Vermont: the next chapter. *Health Affairs, 13*(5), 78-103.
Lemke, A. A., & Harris-Wai, J. N. (2015). Stakeholder engagement in policy development: challenges and opportunities for human genomics. *Genetics in Medicine, 17*(12), 949-957. doi:https://dx.doi.org/10.1038/gim.2015.8
Len-Rios, M., Onyebadi, U., Qiu, Q., & Phillips, M. (2007 2007 Annual Meeting). *The Role of the South African Elite Press in Setting the Agenda For Discussion of HIV/AIDS: Biomedical or Traditional Solutions*.
Leonard, J. (2006). *The role of professional nursing in the origin of the Newborns' and Mothers' Health Protection Act of 1996 from a feminist perspective, 1981--1996.* (Ph.D.), University of Massachusetts Amherst. Retrieved from http://search.ebscohost.com/login.aspx?direct=true&db=rzh&AN=109846608&site=ehost-live Available from EBSCOhost rzh database.
Levi, R. (2009). Making counter-law: On having no apparent purpose in Chicago. *British Journal of Criminology, 49*(2), 131-149. doi:10.1093/bjc/azn080
Levin, I. (2015). Review of Women voicing resistance: Discursive and narrative explorations. *Journal of Family Theory & Review, 7*(3), 340-343.
Levinson, C. J., & Richardson, D. C. (1976). The Dalkon shield story. *Advances in Planned Parenthood, 11*(2), 53-63.
Lewis, S. D., Johnson, V. R., Farris, R. P., & Will, J. C. (2004). Using success stories to share knowledge and lessons learned in health promotion. *Journal of Women's Health, 13*(5), 616-624. doi:https://dx.doi.org/10.1089/1540999041280954
Lieberman, T. (1995). This is the story of the vested interest that hired the firm that fronted the study that skewed the numbers that spread through the press and finished off a vital piece of health care reform. *Columbia Journalism Review, 33*(5), 28-31.
Limb, M. (2009). The story in Wales...Policy in Focus series. *Physiotherapy Frontline, 15*(15), 12-14.
Lindsay, G. M. (2001). *Nothing personal? Narrative reconstruction of Registered Nurses' experience in healthcare reform.* (Ph.D.), University of Toronto (Canada). Retrieved from http://search.ebscohost.com/login.aspx?direct=true&db=rzh&AN=109876203&site=ehost-live Available from EBSCOhost rzh database.

Lindsay, G. M., Mior, S. A., Côté, P., Carroll, L. J., & Shearer, H. M. (2016). Patients' Experiences With Vehicle Collision to Inform the Development of Clinical Practice Guidelines: A Narrative Inquiry. *J Manipulative Physiol Ther, 39*(3), 218-228.

Lindsay, R., & Graham, H. (2000). Relational narratives: solving an ethical dilemma concerning an individual's insurance policy. *Nursing Ethics, 7*(2), 148-157.

Lindsay, S., Selvaraj, S., Macdonald, J. W., & Godden, D. J. (2004). Injuries to Scottish farmers while tagging and clipping cattle: a cross-sectional survey. *Occupational Medicine (Oxford), 54*(2), 86-91.

Lipschitz, D. (2010). *A transpersonal exploration of Ginn Fourie and Letlapa Mphahlele's journey of forgiveness.* (71), ProQuest Information & Learning, US. Retrieved from http://search.ebscohost.com/login.aspx?direct=true&db=psyh&AN=2010-99220-311&site=ehost-live Available from EBSCOhost psyh database.

Lockey, R., Maidment, L., & O'Brien, M. (2011). Plenary 9: Maternity monologues: Stories from Aboriginal and Torres Strait Islander women in the Northern Territory, Australia and their relationship to research and policy. *Women & Birth, 24*, S27-S27. doi:10.1016/j.wombi.2011.07.092

Lokugamage, A. U., & Pathberiya, S. D. (2017). Human rights in childbirth, narratives and restorative justice: a review. *Reproductive Health, 14*(1), 17. doi:https://dx.doi.org/10.1186/s12978-016-0264-3

Lombardo, P. A. (2008). Legal archaeology: discovering the stories behind the cases. *Journal of Law, Medicine & Ethics, 36*(3), 589-593. doi:https://dx.doi.org/10.1111/j.1748-720X.2008.308.x

Long, M. (1998). For a healthier community. *Seminars for Nurse Managers, 6*(1), 51-53.

Lonne, B., & Gillespie, K. (2014). How do Australian print media representations of child abuse and neglect inform the public and system reform?: stories place undue emphasis on social control measures and too little emphasis on social care responses. *Child Abuse & Neglect, 38*(5), 837-850. doi:https://dx.doi.org/10.1016/j.chiabu.2014.04.021

Lovgren, G., Engstrom, B., & Norberg, A. (1996). Patients' narratives concerning good and bad caring. *Scandinavian Journal of Caring Sciences, 10*(3), 151-156.

Lublinski, J., Spurk, C., Fleury, J.-M., Labassi, O., Mbarga, G., Nicolas, M. L., & Rizk, T. A. (2016). Triggering change – How investigative journalists in Sub-Saharan Africa contribute to solving problems in society. *Journalism, 17*(8), 1074-1094. doi:10.1177/1464884915597159

Lüngen, M., Schröer-Günther, M.-A., Passon, A. M., Gerber, A., & Lauterbach, K. W. (2009). Evidenz der Wirksamkeit internationaler Präventionsmassnahmen und Auswirkungen auf ein deutsches Präventionsgesetz. [[Does the lack of evidence of effectiveness among international studies on interventions in prevention and health promotion have an impact on a German Act of Prevention?].]. *Med Klin (Munich), 104*(2), 101-107.

Macdonnell, J. A. (2011). Gender, sexuality and the participatory dimensions of a comparative life history policy study. *Nursing Inquiry, 18*(4), 313-324. doi:https://dx.doi.org/10.1111/j.1440-1800.2011.00524.x

MacDougall, C., Riggs, E., & Lee, V. (2014). Writing a new story for Australia's children. *Australian & New Zealand Journal of Public Health, 38*(3), 203-204. doi:10.1111/1753-6405.12248

Macfarlane, F., Exworthy, M., Wilmott, M., & Greenhalgh, T. (2011). Plus ca change, plus c'est la meme chose: senior NHS managers' narratives of restructuring. *Sociology of Health & Illness, 33*(6), 914-929. doi:https://dx.doi.org/10.1111/j.1467-9566.2011.01338.x

MacGregor, S. (2013a). Barriers to the influence of evidence on policy: Are politicians the problem? *Drugs: Education, Prevention & Policy, 20*(3), 225-233. doi:10.3109/09687637.2012.754403

MacGregor, S. (2013b). Review of Drink and the city: Alcohol and alcohol problems in urban UK since the 1950s. *Drugs: Education, Prevention & Policy, 20*(4), 343-344. doi:10.3109/09687637.2013.776731

Maclennan, B., Kypri, K., Room, R., & Langley, J. (2013). Local government alcohol policy development: case studies in three New Zealand communities. *Addiction, 108*(5), 885-895. doi:https://dx.doi.org/10.1111/add.12017

Maier, S. L. (2008). "I have heard horrible stories . . .": rape victim advocates' perceptions of the revictimization of rape victims by the police and medical system. *Violence Against Women, 14*(7), 786-808. doi:https://dx.doi.org/10.1177/1077801208320245

Malin, N. A., & Race, D. G. (2010). The impact of social policy on changes in professional practice within learning disability services: different standards for children and adults? A two-part examination: Part 1. The policy foundations: from welfare markets to Valuing People, personalization and Baby P. *Journal of Intellectual Disabilities, 14*(4), 315-328. doi:https://dx.doi.org/10.1177/1744629510395072

Manchester, A. (2016). Telling the human story to improve patient safety. *Kai Tiaki Nursing New Zealand, 22*(7), 28-29.

Manchester, A., & O'Connor, T. (2008). The bigger picture. *Nursing Standard, 23*(9), 20-22.

Manchikanti, L. (2006). Prescription drug abuse: what is being done to address this new drug epidemic? Testimony before the Subcommittee on Criminal Justice, Drug Policy and Human Resources. *Pain Physician, 9*(4), 287-321.

Mannion, R., Freeman, T., Millar, R., & Davies, H. (2016). *NIHR Journals Library, Effective board governance of safe care*, a (theoretically underpinned) cross-sectioned examination of the breadth and depth of relationships through national quantitative surveys and in-depth qualitative case studies Health Services and Delivery Research. doi:https://dx.doi.org/10.3310/hsdr04040

Marmor, T. R. (1991). New York's Blue Cross and Blue Shield, 1934-1990: the complicated politics of nonprofit regulation. *Journal of Health Politics, Policy & Law, 16*(4), 761-792.

Martinez, G. (2005). The phoenix rises again. 'One man's story' continues a year after his liver transplant. *Positively Aware: the Monthly Journal of the Test Positive Aware Network, 16*(3), 44-45.

Martins, S. (2007). Temer o quê? - Sair de casa ou ligar a TV? [What to fear? Leaving home or turning on the TV?]. *Psico (Porto Alegre), 38*(3), 278-284.

Mason, T., Wilkinson, G. W., Nannini, A., Martin, C. M., Fox, D. J., & Hirsch, G. (2011). Winning policy change to promote community health workers: lessons from massachusetts in the health reform era. *American Journal of Public Health, 101*(12), 2211-2216. doi:https://dx.doi.org/10.2105/AJPH.2011.300402

Matorin, S., & Ringel, S. P. (2011). Caring For Patients And Much More...'Narrative matters' [June 2011]. *Health Affairs, 30*(9), 1807-1807. doi:10.1377/hlthaff.2011.0879

Mattson, M. (2010). Health advocacy by accident and discipline. *Health Communication, 25*(6-7), 622-624. doi:https://dx.doi.org/10.1080/10410236.2010.496844

Mattson, M. P. (2011). A lifetime of dedication to the old in his Kentucky home. *NeuroMolecular Medicine, 13*(1), 6-10. doi:https://dx.doi.org/10.1007/s12017-010-8124-8

Mayes, R. (2013). Remedy and Reaction: The Peculiar American Struggle over Health Care Reform/ Power, Politics, and Universal Health Care: The Inside Story of a Century-Long Battle. *Journal of Health Politics, Policy & Law, 38*(1), 200-207. doi:10.1215/03616878-1898884

Mays, V. M. (2012). The Legacy of the U. S. Public Health Services Study of Untreated Syphilis in African American Men at Tuskegee on the Affordable Care Act and Health Care Reform Fifteen Years After President Clinton's Apology. *Ethics & Behavior, 22*(6), 411-418. doi:https://dx.doi.org/10.1080/10508422.2012.730808

McConnell-Henry, T. (2012). Recognised but not known: Learning from a rural organ donation experience. *Transplant Journal of Australasia, 21*(2), 18-21.

McCoyd, J. L. M. (2010). Women in no man's land: the abortion debate in the USA and women terminating desired pregnancies due to foetal anomaly. *British Journal of Social Work, 40*(1), 133-153. doi:10.1093/bjsw/bcn080

McCullough, L. B. (2006). John Gregory's medical ethics and the reform of medical practice in eighteenth-century Edinburgh. *Journal of the Royal College of Physicians of Edinburgh, 36*(1), 86-92.
McDonough, J. E. (2001). Using and misusing anecdote in policy making. *Health Affairs, 20*(1), 207-212.
McFarlane, D. R. (1993). U.S. abortion policy since Roe v. Wade. *The American Journal of Gynecologic Health, 7*(4), 98-106.
McGee, E. M. (2005). Using personal narratives to encourage organ donation. *American Journal of Bioethics, 5*(4), 19-20.
McGowan, B. G. (1990). Family-based services and public policy: Context and implications. In J. K. Whittaker, J. Kinney, E. M. Tracy, C. Booth, J. K. Whittaker, J. Kinney, E. M. Tracy, & C. Booth (Eds.), *Reaching high-risk families: Intensive family preservation in human services.* (pp. 65-85). Hawthorne, NY, US: Aldine de Gruyter.
McLntyre, J. J. (2013). The political narrative of children's media research. In K. E. Dill & K. E. Dill (Eds.), *The Oxford handbook of media psychology.* (pp. 462-473). New York, NY, US: Oxford University Press.
McNutt, S. (2009). How the amputee coalition helps: for the amputee community, the ACA offers resources, publications, advocacy and more. *InMotion, 19*(1), 26-28.
Meade, E. (2010). From sex strangler to model citizen: Mexico's most famous murderer and the defeat of the death penalty. *Mexican Studies-Estudios Mexicanos, 26*(2), 323-377.
Mears, J. (2003). Survival is not enough: violence against older women in Australia. *Violence Against Women, 9*(12), 1478-1489.
Meisel, Z. F., & Karlawish, J. (2011). Narrative vs evidence-based medicine--and, not or. *JAMA, 306*(18), 2022-2023. doi:https://dx.doi.org/10.1001/jama.2011.1648
Meisel, Z. F., & Karlawish, J. (2011). Narratives vs evidence-based medicine—And, not or. *JAMA: Journal of the American Medical Association, 306*(18), 2022-2023. doi:10.1001/jama.2011.1648
Meyer, C. R. (1999). Unwitting consent: "Acres of Skin: Human Experiments at Holmesburg Prison" tells the story of medical researchers who sacrificed the rights of their subjects for personal profit. *Minnesota Medicine, 82*(7), 53-54.
Mi Kyung, W., & Miyoung, K. (2013). Nurses' Experiences of Do-Not-Resuscitate (DNR) by the Narrative Inquiry. *Korean Journal of Adult Nursing, 25*(3), 322-331. doi:10.7475/kjan.2013.25.3.322
Middleton, J. (2011). 'Don't let horror stories put you off speaking out'. *Nursing Times, 107*(42), 1-1.
Miller, D. H. (1999). From one voice a chorus: Elizabeth Cady Stanton's 1860 address to the New York state legislature. *Women's Studies in Communication, 22*(2), 152.
Miller, E. A., Tyler, D. A., Rozanova, J., & Mor, V. (2012). National newspaper portrayal of U.S. nursing homes: periodic treatment of topic and tone. *Milbank Quarterly, 90*(4), 725-761. doi:https://dx.doi.org/10.1111/j.1468-0009.2012.00681.x
Miller, J. G. (1997). The press, experts and welfare reform. *Nieman Reports, 51*(1), 49.
Minot, I. H. (2005). Personal accounts: from the depths of despair to a mission of advocacy. *Psychiatric Services, 56*(8), 905-906. doi:https://dx.doi.org/10.1176/appi.ps.56.8.905
Miser, W. F. (2005). Aspirin use for prevention of CHD: an FP's story. *American Family Physician, 71*(2), 250-251, 253.
Mitchell, G. (2013). My epilepsy story: an interview with Gay Mitchell by Astrid Nehlig and Gary Mathern. *Epilepsia, 54*(10), 1711-1712. doi:https://dx.doi.org/10.1111/epi.12370
Monteith, S. K. (1997). AIDS: the untold story... reprinted with permission of the Medical Sentinel Summer 1997 issue pages 97-100. *Revolution: The Journal of Nurse Empowerment, 7*(4), 58-62.
Monterrosa, E. C., Campirano, F., Tolentino Mayo, L., Frongillo, E. A., Hernandez Cordero, S., Kaufer-Horwitz, M., & Rivera, J. A. (2015). Stakeholder perspectives on national policy for regulating

the school food environment in Mexico. *Health Policy & Planning, 30*(1), 28-38. doi:https://dx.doi.org/10.1093/heapol/czt094
Moodie, R. (2004). [Measuring the effectiveness of health promotion policy: what can be learnt from Australian success stories?]. *Promotion et Education, Spec no 1*, 28-32, 49.
Moore, M. (2012). The global dimensions of public health preparedness and implications for US action. *American Journal of Public Health, 102*(6), e1-7. doi:https://dx.doi.org/10.2105/AJPH.2011.300644
Morantz, G., & Heymann, J. (2010). Life in institutional care: the voices of children in a residential facility in Botswana. *AIDS Care, 22*(1), 10-16. doi:https://dx.doi.org/10.1080/09540120903012601
Morgan, J. (2005). The Power of Storytelling: A Quest for a Public Discourse on Sexual Harassment. *International Journal of Discrimination and the Law, 7*(1-4), 5-28. doi:10.1177/135822910500700402
Morrison, M., & Gillett, G. (2014). Is a cleft lip and palate a serious "handicap"? Jepson v Chief Constable of West Mercia--a legal and ethical critique. *Journal of Law & Medicine, 22*(2), 290-301.
Mucciaroni, G., & Killian, M. L. (2004). Immutability, science, and legislative debate over gay, lesbian and bisexual rights. *Journal of Homosexuality, 47*(1), 53-77. doi:https://dx.doi.org/10.1300/J082v47n01_04
Muench, S. D. (2010). Losing everything...a sad, but true story. *Pennsylvania Dental Journal, 77*(3), 29-31.
Mullan, F., Ficklen, E., & Rubin, K. (2006). *Narrative matters: The power of personal essay in health policy*. Baltimore, MD, US: Johns Hopkins University Press.
Mullen, E. (1999). Delivering a promise: one woman's story of partnership and hope. *Plastic Surgical Nursing, 19*(3), 117-120.
Mullen, K. (1989). Glasgow's temperance story: changing approaches towards the control of alcohol abuse. *Health Bulletin, 47*(6), 304-310.
Murphy, A. M. (2009). Early childhood development: One developmental paediatrician's story. *Paediatrics & Child Health, 14*(10), 669-670.
Murray, J. E. (2001). Supreme Court decision paves way for home care. *Caring, 20*(6), 24-29.
Nedlund, A. C., & Nordh, J. (2015). Crafting citizen(ship) for people with dementia: How policy narratives at national level in Sweden informed politics of time from 1975 to 2013. *Journal of Aging Studies, 34*, 123-133. doi:https://dx.doi.org/10.1016/j.jaging.2015.06.003
Nettleton, S., & Harding, G. (1994). Protesting patients: a study of complaints submitted to a Family Health Service Authority. *Sociology of Health & Illness, 16*(1), 38-61.
Neville, S., & Chenery, K. (1996). "Once upon a time ..." story telling: a paediatric perspective. *Nursing Praxis in New Zealand, 11*(3), 14-18.
Newdick, C., & Danbury, C. (2015). Culture, compassion and clinical neglect: probity in the NHS after Mid Staffordshire. *Journal of Medical Ethics, 41*(12), 956-962. doi:10.1136/medethics-2012-101048
Newman, C., & Persson, A. (2009). Fear, complacency and the spectacle of risk: the making of HIV as a public concern in Australia. *Health: An Interdisciplinary Journal for the Social Study of Health, Illness & Medicine, 13*(1), 7-23. doi:https://dx.doi.org/10.1177/1363459308097358
Nichols, L. T. (1997). Social Problems as Landmark Narratives: Bank of Boston, Mass Media and "Money Laundering.". *Social Problems, 44*(3), 324-341.
Nichols, S. L., Friedland, L. A., Rojas, H., Cho, J., & Shah, D. V. (2006). EXAMINING THE EFFECTS OF PUBLIC JOURNALISM ON CIVIL SOCIETY FROM 1994 TO 2002: ORGANIZATIONAL FACTORS, PROJECT FEATURES, STORY FRAMES, AND CITIZEN ENGAGEMENT. *Journalism & Mass Communication Quarterly, 83*(1), 77-100.
Niechajev, I., & Frame, J. (2012). A plea to control medical tourism. *Aesthetic Plastic Surgery, 36*(1), 202-206. doi:https://dx.doi.org/10.1007/s00266-011-9766-0

Niederdeppe, J., Heley, K., & Barry, C. L. (2015). Inoculation and narrative strategies in competitive framing of three health policy issues. *Journal of Communication, 65*(5), 838-862. doi:10.1111/jcom.12162

Niederdeppe, J., Kim, H. K., Lundell, H., Fazili, F., & Frazier, B. (2012). Beyond counterarguing: Simple elaboration, complex integration, and counterelaboration in response to variations in narrative focus and sidedness. *Journal of Communication, 62*(5), 758-777. doi:10.1111/j.1460-2466.2012.01671.x

Niederdeppe, J., Robert, S. A., & Kindig, D. A. (2011). Qualitative research about attributions, narratives, and support for obesity policy, 2008. *Preventing Chronic Disease, 8*(2), A39.

Niederdeppe, J., Roh, S., & Dreisbach, C. (2016). How Narrative Focus and a Statistical Map Shape Health Policy Support Among State Legislators. *Health Communication, 31*(2), 242-255. doi:https://dx.doi.org/10.1080/10410236.2014.998913

Niederdeppe, J., Roh, S., & Shapiro, M. A. (2015). Acknowledging individual responsibility while emphasizing social determinants in narratives to promote obesity-reducing public policy: a randomized experiment. *PLoS ONE [Electronic Resource], 10*(2), e0117565. doi:https://dx.doi.org/10.1371/journal.pone.0117565

Niederdeppe, J., Roh, S., Shapiro, M. A., & Kim, H. K. (2013). Effects of messages emphasizing environmental determinants of obesity on intentions to engage in diet and exercise behaviors. *Preventing Chronic Disease, 10*, E209. doi:https://dx.doi.org/10.5888/pcd10.130163

Niederdeppe, J., Shapiro, M., & Porticella, N. (2010). Effects of a Narrative and Summary of Scientific Evidence on Attributions of Responsibility for Obesity. *Conference Papers -- International Communication Association*, 1.

Norko, M. A. (2010). Commentary: the dynamic evolution of forensic psychiatry at Yale and the Zonanian sphere of influence. *Journal of the American Academy of Psychiatry & the Law, 38*(4), 577-580.

Novak, S. J. (1998). Second thoughts on psychedelic drugs. *Endeavour, 22*(1), 21-23.

Nunes, M., Juca, V. J., & Valentim, C. P. (2007). [Mental health care in the Family Health Program: consensus and dissent in practices and principles under the psychiatric reform and health reform in Brazil]. *Cadernos de Saude Publica, 23*(10), 2375-2384.

Nunn, A., Dickman, S., Nattrass, N., Cornwall, A., & Gruskin, S. (2012). The impacts of AIDS movements on the policy responses to HIV/AIDS in Brazil and South Africa: a comparative analysis. *Global Public Health, 7*(10), 1031-1044. doi:https://dx.doi.org/10.1080/17441692.2012.736681

O'Connor, M. (2005). *The Center for Nursing Leadership: A qualitative study describing the impact and meaning of life changes of individual members.* (Ph.D.), George Mason University. Retrieved from http://search.ebscohost.com/login.aspx?direct=true&db=rzh&AN=109845146&site=ehost-live Available from EBSCOhost rzh database.

O'Donovan, D. (2006). Moving Away from "Failing Boys" and "Passive Girls": Gender meta-narratives in gender equity policies for Australian schools and why micro-narratives provide a better policy model. *Discourse: Studies in the Cultural Politics of Education, 27*(4), 475-494. doi:10.1080/01596300600988655

O'Neill, K. L. (2007). Armed Citizens and the Stories They Tell. *Men & Masculinities, 9*(4), 457-475. doi:10.1177/1097184X05281390

Ofuji, K. (2007). Sexual minority issues and human rights education in Japan. *Journal of Gay & Lesbian Issues In Education, 4*(4), 131-135.

Ohemeng, F. L. K. (2013). Review of Governing narratives: Symbolic politics and policy change. *The American Review of Public Administration, 43*(6), 752-754. doi:10.1177/0275074013482749

Oliveira Friestino, J. K. (2016). ACTIONS FOR COPING DOMESTIC VIOLENCE AGAINST CHILDREN AND TEENAGERS: A REFLECTIVE ANALYSIS. *Journal of Nursing UFPE / Revista de Enfermagem UFPE*, 341-346. doi:10.5205/reuol.7901-80479-1-SP.1001sup201620

Oman, K. J. (2009). *Framing the picture: Racial profiling and the public discourse.* (70), ProQuest Information & Learning, US. Retrieved from http://search.ebscohost.com/login.aspx?direct=true&db=psyh&AN=2009-99231-064&site=ehost-live Available from EBSCOhost psyh database.

Pachem, T. (2008). 'A true role model'. *Behavioral Healthcare, 28*(3), 14.

Pandya, A. (2012). NAMI in our own voice and NAMI smarts for advocacy: self-narrative as advocacy tool. *Journal of Psychiatric Practice, 18*(6), 448-450. doi:https://dx.doi.org/10.1097/01.pra.0000422744.79871.1a

Paterson, B. (2006). Newspaper representations of mental illness and the impact of the reporting of "events" on social policy: the "framing" of Isabel Schwarz and Jonathan Zito. *Journal of Psychiatric & Mental Health Nursing, 13*(3), 294-300. doi:https://dx.doi.org/10.1111/j.1365-2850.2006.00953.x

Paterson, R. (2015). “Just ask Judi” - a patient's story of care at the end of life...Judi Strid. *Midwifery News*(78), 36-39.

Pavlish, C., & Ceronsky, L. (2009). Oncology nurses' perceptions of nursing roles and professional attributes in palliative care. *Clinical Journal of Oncology Nursing, 13*(4), 404-412. doi:https://dx.doi.org/10.1188/09.CJON.404-412

Payne, C. M., Debbink, M. P., Steele, E. A., Buck, C. T., Martin, L. A., Hassinger, J. A., & Harris, L. H. (2013). Why women are dying from unsafe abortion: narratives of Ghanaian abortion providers. *African Journal of Reproductive Health, 17*(2), 118-128.

Peabody, J. W., Hesketh, T. M., & Steinberg, P. S. (1996). Translating clinical practice into health policy: an example from China. *Health Policy, 35*(2), 107-121.

Penn, C. L. (2014). Kathy Webb. Longtime advocate fights her own battle with breast cancer. *Journal of the Arkansas Medical Society, 111*(5), 80-81.

Penn, C. L. (2015). The Story of the Telemedicine Act. AMS-Supported Legislation Becomes Law. *Journal of the Arkansas Medical Society, 112*(5), 54-56.

Pentecost, M. J. (2004). Medicare reform legislation: a new chapter in a long story. *Journal of the American College of Radiology, 1*(5), 308-309. doi:https://dx.doi.org/10.1016/j.jacr.2004.01.012

Perez-Botella, M., & Downe, S. (2006). Stories as evidence: why do midwives still use directed pushing? *British Journal of Midwifery, 14*(10), 596-599.

Pestka, E. L., & Shea, C. E. (2016). Advocating for the Use of Pharmacogenomics: One Nurse's Story. *Journal of Psychosocial Nursing & Mental Health Services, 54*(7), 38-42. doi:https://dx.doi.org/10.3928/02793695-20160616-07

Petrunik, M., & Weisman, R. (2005). Constructing Joseph Fredericks: competing narratives of a child sex murderer. *International Journal of Law & Psychiatry, 28*(1), 75-96. doi:https://dx.doi.org/10.1016/j.ijlp.2004.12.005

Pickering, B. A. (2003). WOMEN'S VOICES AS EVIDENCE: PERSONAL TESTIMONY IS PRO-CHOICE FILMS. *Argumentation & Advocacy, 40*(1), 1-22.

Poltorak, M., Leach, M., Fairhead, J., & Cassell, J. (2005). 'MMR talk' and vaccination choices: an ethnographic study in Brighton. *Social Science & Medicine, 61*(3), 709-719. doi:https://dx.doi.org/10.1016/j.socscimed.2004.12.014

Power and Effectiveness of Nurses. (2016). *Tennessee Nurse, 79*(2), 11-12.

Price, K. (2011). The quest for purity: The role of policy narratives in determining teen girls’ access to emergency contraception in the USA. *Sexuality Research & Social Policy: A Journal of the NSRC, 8*(4), 282-293. doi:10.1007/s13178-011-0068-8

Pulkingham, J., Fuller, S., & Kershaw, P. (2010). Lone motherhood, welfare reform and active citizen subjectivity. *Critical Social Policy, 30*(2), 267-291. doi:10.1177/0261018309358292

Pullman, D., Zarzeczny, A., & Picard, A. (2013). "Media, politics and science policy: MS and evidence from the CCSVI Trenches". *BMC Medical Ethics, 14*, 6. doi:https://dx.doi.org/10.1186/1472-6939-14-6
Purtle, J., Dodson, E. A., & Brownson, R. C. (2016). Uses of Research Evidence by State Legislators Who Prioritize Behavioral Health Issues. *Psychiatric Services, 67*(12), 1355-1361. doi:https://dx.doi.org/10.1176/appi.ps.201500443
Ridings, J. E. (2013). The thalidomide disaster, lessons from the past. *Methods in Molecular Biology, 947*, 575-586. doi:https://dx.doi.org/10.1007/978-1-62703-131-8_36
Rifkin, S. (2017). Safeguarding the ADA's Antidiscrimination Mandate: Subjecting Arrests to Title II Coverage. *Duke Law J, 66*(4), 913-941.
Roberts, R. G., Gask, L., Arndt, B., Bower, P., Dunbar, J., van der Feltz-Cornelis, C. M., . . . Anderson, M. I. (2012). Depression and diabetes: the role and impact of models of health care systems. *Journal of Affective Disorders, 142 Suppl*, S80-88. doi:https://dx.doi.org/10.1016/S0165-0327(12)70012-5
Robertson, J., Walkom, E. J., Bevan, M. D., & Newby, D. A. (2013). Medicines and the media: news reports of medicines recommended for government reimbursement in Australia. *BMC Public Health, 13*, 489. doi:https://dx.doi.org/10.1186/1471-2458-13-489
Rochefort, D. A. (1998). The role of anecdotes in regulating managed care. *Health Affairs, 17*(6), 142-149.
Rodkey, E. N. (2016). Far More Than Dutiful Daughter: Milicent Shinn's Child Study and Education Advocacy After 1898. *J Genet Psychol, 177*(6), 209-230.
Rosenberg, H. M. (2009). *Federal policy toward delinquent youth: Legislative and programmatic milestones from Kennedy to Ford, 1960-1976.* (70), ProQuest Information & Learning, US. Retrieved from http://search.ebscohost.com/login.aspx?direct=true&db=psyh&AN=2009-99130-111&site=ehost-live Available from EBSCOhost psyh database.
Rubin, R. (2012). The right to give life. *POZ*, 28-31.
Rubotzky, A. M. (2000). Nursing participation in health care reform efforts of 1993 to 1994: advocating for the national community. *Advances in Nursing Science, 23*(2), 12-33.
Russell, A., Voas, R. B., Dejong, W., & Chaloupka, M. (1995). MADD rates the states: a media advocacy event to advance the agenda against alcohol-impaired driving. *Public Health Reports, 110*(3), 240-245.
Ryan, B. A. (2003). Legally speaking. Do you suspect child abuse? *RN, 66*(9), 73-55.
Sammy, C. M. (2014). Kitui district hospital's newborn unit -- Success story: Transforming a nursery room into an independent newborn unit. *Journal of Neonatal Nursing, 20*(2), 60-64. doi:10.1016/j.jnn.2013.12.001
Sass, R. (1999). The unwritten story of women's role in the birth of occupational health and safety legislation. *Int J Health Serv, 29*(1), 109-145.
Scarparo, H. B. K., Accorssi, A., & Pizzinato, A. (2011). Contextos , processos e memórias: narrativas sobre saúde mental nas décadas de sessenta a oitenta no Brasil. [Contexts, processes and memory: narratives about mental-healthcare from the 1960's to the 1980's in Brazil]. *Estud. pesqui. psicol. (Impr.), 11*(1), 333-352.
Scherger, J. E. (2014). The unfinished story of family medicine transformation. *Family Medicine, 46*(1), 5-6.
Schmied, V., Sheehan, A., & Barclay, L. (2001). Contemporary breast-feeding policy and practice: implications for midwives. *Midwifery, 17*(1), 44-54. doi:https://dx.doi.org/10.1054/midw.2000.0234
Schuftan, C. (2015). COMMENTARY: GLOBALIZATION, HEALTH SECTOR REFORM, AND THE HUMAN RIGHT TO HEALTH: IMPLICATIONS FOR FUTURE HEALTH POLICY. *Int J Health Serv, 45*(1), 187-193.
Sellman, D. (1997). Child B: a case of just care? *European Journal of Cancer Care, 6*(4), 245-248.

Sentell, T. L., Seto, T. B., Young, M. M., Vawer, M., Quensell, M. L., Braun, K. L., & Taira, D. A. (2016). Pathways to potentially preventable hospitalizations for diabetes and heart failure: a qualitative analysis of patient perspectives. *BMC Health Services Research, 16*, 300. doi:https://dx.doi.org/10.1186/s12913-016-1511-6

Shanner, L. (1997). Teaching women's health issues in a government committee: the story of a successful policy group. *Womens Health Issues, 7*(6), 393-399.

Shaw, S. E. (2010). Reaching the parts that other theories and methods can't reach: how and why a policy-as-discourse approach can inform health-related policy. *Health: An Interdisciplinary Journal for the Social Study of Health, Illness & Medicine, 14*(2), 196-212. doi:https://dx.doi.org/10.1177/1363459309353295

Sherer, R. A. (2008). A double standard of care for mental and physical illness -- the story of Esmin Green. *Psychiatric Times, 25*(10), 1-7.

Sheringham, J., Baraitser, P., Simms, I., Hart, G., & Raine, R. (2012). Chlamydia screening in England: a qualitative study of the narrative behind the policy. *BMC Public Health, 12*, 317. doi:https://dx.doi.org/10.1186/1471-2458-12-317

Sherrod, M. M. (2006). Colice's story and the effects of generational loss. *Western Journal of Nursing Research, 28*(7), 754-777; discussion 778-785. doi:https://dx.doi.org/10.1177/0193945906286812

Shickle, D., Day, M., Smith, K., Zakariasen, K., Moskol, J., & Oliver, T. (2014). Mind the public health leadership gap: the opportunities and challenges of engaging high-profile individuals in the public health agenda. *Journal of Public Health, 36*(4), 562-567. doi:https://dx.doi.org/10.1093/pubmed/fdu003

Short, N. (2006). From classroom to Congress. My year on the legislative staff of the U.S. Senate.[Erratum appears in Am J Nurs. 2006 Apr;106(4):15]. *American Journal of Nursing, 106*(2), 70-71.

Siciliano, A. M. (2012). *Policing poverty race, space and the fear of crime after the Year of the Gun (2005) in suburban Toronto.* (72), ProQuest Information & Learning, US. Retrieved from http://search.ebscohost.com/login.aspx?direct=true&db=psyh&AN=2012-99010-488&site=ehost-live Available from EBSCOhost psyh database.

Siegal, D., & Ruoff, G. (2015). Data as a catalyst for change: Stories from the frontlines. *Journal of Healthcare Risk Management, 34*(3), 18-25. doi:10.1002/jhrm.21161

Silvén Hagström, A. (2014). "The self-murderer from Orminge". *Narrative Inquiry, 24*(2), 218-238. doi:10.1075/ni.24.2.03hag

Silver, D. (2001). Songs and storytelling: bringing health messages to life in Uganda. *Education for Health, 14*(1), 51-60. doi:https://dx.doi.org/10.1080/13576280010015362

Sinclair, C. A., Makin, J. K., Tang, A., Brozek, I., & Rock, V. (2014). The role of public health advocacy in achieving an outright ban on commercial tanning beds in Australia. *American Journal of Public Health, 104*(2), e7-9. doi:https://dx.doi.org/10.2105/AJPH.2013.301703

Sistrom, M. G. (2010). Oregon's senate bill 560: practical policy lessons for nurse advocates. *Policy, Politics & Nursing Practice, 11*(1), 29-35. doi:10.1177/1527154410370786

Slasberg, C., & Beresford, P. (2016). The false narrative about personal budgets in England: smoke and mirrors? *Disability & Society, 31*(8), 1132-1137. doi:10.1080/09687599.2016.1235309

Slater, M. D., Hayes, A. F., & Chung, A. H. (2015). Injury news coverage, relative concern, and support for alcohol-control policies: an impersonal impact explanation. *Journal of Health Communication, 20*(1), 51-59. doi:https://dx.doi.org/10.1080/10810730.2014.906523

Slater, M. D., Long, M., & Ford, V. L. (2006). Alcohol, illegal drugs, violent crime, and traffic-related and other unintended injuries in U.S. local and national news. *Journal of Studies on Alcohol, 67*(6), 904-910.

Smidt, C. D. (2012). Not All News Is the Same. *Public Opinion Quarterly, 76*(1), 72-94.

Smith, A. (2008). Whisperings of ovarian cancer: acknowledging women's voices. *Clinical Journal of Oncology Nursing, 12*(6), 913-920. doi:https://dx.doi.org/10.1188/08.CJON.913-920

Smith, C. (1998). Visual evidence in environmental catastrophe TV stories. *Journal of Mass Media Ethics, 13*(4), 247.
Smith, C. J. (2007). Mental health policy: Implications for African Americans. In S. M. L. Logan, R. W. Denby, P. A. Gibson, S. M. L. Logan, R. W. Denby, & P. A. Gibson (Eds.), *Mental health care in the African-American community.* (pp. 233-252). New York, NY, US: Haworth Press.
Smith, D. C. (2014). The Real Story Behind the Nation's First Shield Law: Maryland, 1894–1897. *Communication Law & Policy, 19*(1), 3-53. doi:10.1080/10811680.2014.860828
Smith, J. A. (2014). Panel IV: The Future of the Press and Secrecy. *Communication Law & Policy, 19*(1), 129-139. doi:10.1080/10811680.2014.860836
Smith, K. C., Terry-McElrath, Y., Wakefield, M., & Durrant, R. (2005). Media advocacy and newspaper coverage of tobacco issues: a comparative analysis of 1 year's print news in the United States and Australia. *Nicotine & Tobacco Research, 7*(2), 289-299. doi:https://dx.doi.org/10.1080/14622200500056291
Smith, M. (1993). Two legs to stand on... left above-the-knee amputation. *American Journal of Nursing, 93*(12), 42-44.
Smith, M. L., & Flamm, A. L. (2011). Accommodating religious beliefs in the ICU: a narrative account of a disputed death. *Narrative Inquiry in Bioethics, 1*(1), 55-64. doi:https://dx.doi.org/10.1353/nib.2011.0003
Smith, R. F. (2015). Narratives of Public Health in Dickens's Journalism: The Trouble with Sanitary Reform. *Lit Med, 33*(1), 157-183.
Smyth, I. (1992). The Indonesian Family Planning Programme: a success story for women? *Newsletter: Women's Global Network on Reproductive Rights*(38), 13-15.
Sofer, D. (2017). The Top Health Care Policy News Stories of 2016. *American Journal of Nursing, 117*(1), 14. doi:https://dx.doi.org/10.1097/01.NAJ.0000511549.92253.18
Solleder, M. K. (1981). Legislative action for health education: the North Carolina story. *Health Education, 12*(6), 26-28.
Solomon, J. (1996). Playing god in the newsroom. *Columbia Journalism Review, 34*(5), 12-14.
Speaks, G. E. (1996). Documenting inadequate care in the nursing home: the story of an undercover agent. *Journal of Elder Abuse & Neglect, 8*(3), 37-45.
Spear, S. (2006). The breast implant story...editor's note: this article is reprinted with permission from Lippincott Williams & Wilkins, Annals of Plastic Surgery, 2006;56(5):573-583. *Plastic Surgical Nursing, 26*(3), 132-144.
Spike, J. P. (2007). Memory identity and capacity. *Journal of Clinical Ethics, 18*(3), 252-255.
Spike, J. P. (2011). When ethics consultation and courts collide: a case of compelled treatment of a mature minor. *Narrative Inquiry in Bioethics, 1*(2), 123-131. doi:https://dx.doi.org/10.1353/nib.2011.0035
Spivack, M. P. (1994). Pathways to policy: a personal perspective... the National Head Injury Foundation. *Journal of Head Trauma Rehabilitation, 9*(2), 82-93.
Squier, S. M. (1999). Narrating genetic disabilities: social constructs, medical treatment, and public policy. *Issues in Law & Medicine, 15*(2), 141-158.
Stamm, B. H. (2007). Lift every voice and sing—JRMH's first year. *Journal of Rural Mental Health, 31*(4), 1-4. doi:10.1037/h0095973
Stanley, T., Plessis, R. D., & Austrin, T. (2011). Making Networks Work. *Qualitative Social Work, 10*(1), 49-65. doi:10.1177/1473325010379629
Starr, J., & Zawacki, B. E. (1999). Voices from the silent world of doctor and patient. *Cambridge Quarterly of Healthcare Ethics, 8*(2), 129-138.
Steele, W. R., Mebane, F., Viswanath, K., & Solomon, J. (2005). News media coverage of a women's health contraversy: how newspapers and TV outlets covered a recent debate over screening mammography. *Women & Health, 41*(3), 83-97. doi:https://dx.doi.org/10.1300/J013v41n03_05

Stein, P. (2004). Clinical exemplar. Pushing through barriers to advocate for a patient [corrected] [published erratum appears in AORN J 2005 Jan;81(1):14]. *AORN Journal, 80*(3), 553-558.
Stein, P. S. (1997). Clinical exemplar demonstrates patient advocacy role of perioperative nurses employed in industry. *AORN Journal, 65*(1), 120.
Stein, R. G. (2010). A many-storied gathering: tales from the 2010 Aging in America Conference. *Aging Today, 31*(2), 16-16.
Steiner, J. F. (2005). The use of stories in clinical research and health policy. *JAMA, 294*(22), 2901-2904. doi:https://dx.doi.org/10.1001/jama.294.22.2901
Stelfox, H. T., Lane, D., Boyd, J. M., Taylor, S., Perrier, L., Straus, S., . . . Zuege, D. J. (2015). A scoping review of patient discharge from intensive care: opportunities and tools to improve care. *Chest, 147*(2), 317-327. doi:https://dx.doi.org/10.1378/chest.13-2965
Stephens, S. (2012). It Was a Dark and Stormy Night. *PT in Motion, 4*(2), 18-28.
Stevens, A., & Ritter, A. (2013). How can and do empirical studies influence drug policies? Narratives and complexity in the use of evidence in policy making. *Drugs: Education, Prevention & Policy, 20*(3), 169-174. doi:10.3109/09687637.2013.793892
Stewart, J. (2004). The Shared Terrain of Narrative Medicine and Advocacy Journalism. *Permanente Journal, 8*(2), 80-81.
Stewart, J. (2007). Story-based health policy: an interview with fitzhugh mullan, MD. *Permanente Journal, 11*(4), 76-78.
Stilgoe, J. (2007). The (co-)production of public uncertainty: UK scientific advice on mobile phone health risks. *Public Understanding of Science, 16*(1), 45-61.
Stockton, E. L. (1966). The Pittsburgh air pollution control story. *American Industrial Hygiene Association Journal, 27*(5), 469-474. doi:https://dx.doi.org/10.1080/00028896609342455
Strack, R. D. (2010). Reforms story encourages RN pursuing NP degree. *Nursing Spectrum -- New England Edition, 14*(6), 6-6.
Strauss, B. S. (2009). Genetic counseling for thalassemia in the Islamic Republic of Iran. *Perspectives in Biology & Medicine, 52*(3), 364-376. doi:https://dx.doi.org/10.1353/pbm.0.0093
Sudhanshu, S., Pankaj, A., Sorabh, J., & Nidhi, S. (2014). Dental Diseases of Acid Factory Workers Globally-Narrative Review Article. *Iranian Journal of Public Health, 43*(1), 1-5.
Swann, J. P. (2016). The history of efforts to regulate dietary supplements in the USA. *Drug Testing & Analysis, 8*(3-4), 271-282. doi:https://dx.doi.org/10.1002/dta.1919
Sy, A., & Spinelli, H. (2016). [Political dimensions of an epidemic: the case of influenza A (H1N1) in the Argentine press]. *Cadernos de Saude Publica, 32*(3), e00188414. doi:https://dx.doi.org/10.1590/0102-311X00188414
Szostak, D. C. (2010). Something More to the Story. *Journal of Legal Medicine, 31*(4), 443-454. doi:10.1080/01947648.2010.535429
Taking their side: the power of storytelling. (2013). *Journal of Dementia Care, 21*(1), 23-25.
Tanne, J. H. (2006). Reviews. Can stories of personal tragedy spark a healthcare revolution? *BMJ: British Medical Journal (International Edition), 333*(7574), 924-924. doi:10.1136/bmj.333.7574.924
Tastet, C. (2010). Collin's journey: a 9-year-old's story about being an advocate. *Volta Voices, 17*(5), 44-45.
Thomas, I. (2017). Pushing policy that promotes equity in active living - From the outside and from the inside. *Preventive Medicine, 95S*, S148-S150. doi:https://dx.doi.org/10.1016/j.ypmed.2016.11.026
Tickle, L. (2006). Nobody's talking. *Community Care*(1628), 30-31.
Tiefer, L. (2006). The Viagra Phenomenon. *Sexualities, 9*(3), 273-294.
Tiffany, F. (1891a). The 12,225,000 acre bill (Sessions 1850-51) *Life of Dorothea Lynde Dix.* (pp. 180-186). Boston, MA, US: Houghton, Mifflin and Company.
Tiffany, F. (1891b). At home and at work again *Life of Dorothea Lynde Dix.* (pp. 307-325). Boston, MA, US: Houghton, Mifflin and Company.

Tiffany, F. (1891c). The Channel Islands *Life of Dorothea Lynde Dix.* (pp. 258-277). Boston, MA, US: Houghton, Mifflin and Company.
Tiffany, F. (1891d). My first-born child *Life of Dorothea Lynde Dix.* (pp. 104-120). Boston, MA, US: Houghton, Mifflin and Company.
Tiffany, F. (1891e). Success of the first memorial *Life of Dorothea Lynde Dix.* (pp. 83-93). Boston, MA, US: Houghton, Mifflin and Company.
Tolman, D. L., Hirschman, C., & Impett, E. A. (2005). There is more to the story: The place of qualitative research on female adolescent sexuality in policy making. *Sexuality Research & Social Policy: A Journal of the NSRC, 2*(4), 4-17. doi:10.1525/srsp.2005.2.4.4
Torronen, J. (2003). The Finnish press's political position on alcohol between 1993 and 2000. *Addiction, 98*(3), 281-290.
Trickett, E. J., Trimble, J. E., & Allen, J. (2014). Most of the story is missing: advocating for a more complete intervention story. *American Journal of Community Psychology, 54*(1-2), 180-186. doi:https://dx.doi.org/10.1007/s10464-014-9645-3
Troy, L. M., & Kietzman, K. G. (2016). Enhancing Evidence-Based Public Health Policy: Developing and Using Policy Narratives. *Journal of Gerontological Nursing, 42*(6), 11-17. doi:https://dx.doi.org/10.3928/00989134-20160516-04
True, G., & Phipps, E. J. (1999). Narratives in rehabilitation. *Journal of Head Trauma Rehabilitation, 14*(5), 505-507.
Trupin, S. (1993). A nursing success story: moral support for 'grandparents who care'. *American Journal of Nursing, 93*(4), 52-56.
Tumber, H. (2004). Scandal and Media in the United Kingdom. *American Behavioral Scientist, 47*(8), 1122-1137. doi:10.1177/0002764203262280
Turner, G. (1994). Exemplar. Advocacy in action. *Nursing Praxis in New Zealand, 9*(3), 33-34.
Tutton, R. (2009). Review of Inclusion: The politics of difference in medical research. *Sociology, 43*(4), 792-793. doi:10.1177/00380385090430041403
Udell, T., & Mehta, K. (2008). When two sides go to war: newspaper reporting of 'television food advertising restrictions' as a solution to childhood obesity. *Health, Risk & Society, 10*(6), 535-548.
Usdin, S. (2009). The heparin story. *International Journal of Risk & Safety in Medicine, 21*(1/2), 93-103.
Van Allen, G. (1996). Focus. Career options: the increasing demand for courtroom nurses. *Insight: The Journal of the American Society of Ophthalmic Registered Nurses, 21*(4), 129-130.
van Amerongen, D. (1999). Using anecdotes: having it both ways? *Health Affairs, 18*(4), 202-203.
van Bekkum, J. E., & Hilton, S. (2013). Primary care nurses' experiences of how the mass media influence frontline healthcare in the UK. *BMC Family Practice, 14*, 178. doi:https://dx.doi.org/10.1186/1471-2296-14-178
van Bekkum, J. E., & Hilton, S. (2013). Primary care nurses' experiences of how the mass media influence frontline healthcare in the UK. *BMC Fam Pract, 14*, 178-178.
Vargas, E., Becerril-Montekio, V., Gonzalez-Block, M. A., Akweongo, P., Hazel, C. N., Cuembelo Mde, F., . . . Munoz, F. (2016). Mapping the use of research to support strategies tackling maternal and child health inequities: evidence from six countries in Africa and Latin America.[Erratum appears in Health Res Policy Syst. 2016;14:26; PMID: 27052198]. *Health Research Policy & Systems, 14*, 1. doi:https://dx.doi.org/10.1186/s12961-015-0072-1
Vega, L. (2011). Mary's story: a crisis of care and a call for advocacy. *Aging Today, 32*(4), 11-11.
Vilasboas, A. L., & Paim, J. S. (2008). [Municipal health policy planning and implementation]. *Cadernos de Saude Publica, 24*(6), 1239-1250.
von Klan, A. (2015). Removing the Mask: Hopeless Isolation to Intersex Advocacy. *Narrative Inquiry in Bioethics, 5*(2), E14-17. doi:https://dx.doi.org/10.1353/nib.2015.0041
von Tigerstrom, B. (2000). The "hidden story" of Bill C-54: the Personal Information Protection and Electronic Documents Act and health information. *Health Law Review, 8*(2), 13-21.

Wagner, J. A. (2010). The ever-changing face of breast advocacy. *Breast, 19*(4), 280-283. doi:https://dx.doi.org/10.1016/j.breast.2010.03.016

Wald, H., Richard, A., Dickson, V. V., & Capezuti, E. (2012). Chief nursing officers' perspectives on Medicare's hospital-acquired conditions non-payment policy: implications for policy design and implementation. *Implementation Science, 7*, 78. doi:https://dx.doi.org/10.1186/1748-5908-7-78

Wald, H., Richard, A., Dickson, V. V., & Capezuti, E. (2012). Chief nursing officers' perspectives on Medicare's hospital-acquired conditions non-payment policy: implications for policy design and implementation. *Implement Sci, 7*, 78-78.

Walker, C. A., Cohen, H., & Jenkins, D. (2016). An Older Transgender Woman's Quest for Identity. *J Psychosoc Nurs Ment Health Serv, 54*(2), 31-38.

Walker, N., Bryce, J., & Black, R. E. (2007). Interpreting health statistics for policymaking: the story behind the headlines. *Lancet, 369*(9565), 956-963. doi:https://dx.doi.org/10.1016/S0140-6736(07)60454-1

Wallington, S. F., Blake, K., Taylor-Clark, K., & Viswanath, K. (2010). Antecedents to agenda setting and framing in health news: an examination of priority, angle, source, and resource usage from a national survey of U.S. health reporters and editors.[Erratum appears in J Health Commun. 2010 Jul;15(5):574]. *Journal of Health Communication, 15*(1), 76-94. doi:https://dx.doi.org/10.1080/10810730903460559

Wallington, S. F., Blake, K., Taylor-Clark, K., Viswanath, K., Wallington, S. F., Blake, K., . . . Viswanath, K. (2010). Antecedents to agenda setting and framing in health news: an examination of priority, angle, source, and resource usage from a national survey of U.S. health reporters and editors. *Journal of Health Communication, 15*(1), 76-94. doi:10.1080/10810730903460559

Wallis, A. B., & Guyer, B. (2006). Myron Wegman: Early Days, Lasting Influence. *Maternal and Child Health Journal, 10*(1), 5-11. doi:10.1007/s10995-005-0033-3

Walter, B. (1983). A little music: why the dying aren't allowed to die... a woman's personal story of her father's needlessly prolonged dying. *NursingLife, 3*(5), 52-57.

Walton, M. (2004). Energy BOOST. *American Journalism Review, 26*(5), 52-57.

Wang, H. H., Wang, J. J., Zhou, Z. H., Wang, X. W., & Xu, L. (2013). General practice education and training in southern China: recent development and ongoing challenges under the health care reform. *Malaysian Family Physician, 8*(3), 2-10.

Wang, J. Y., Thistlethwaite, J. R., & Ross, L. F. (2015). Adding Another Voice to the Living Donor Kidney Transplant Discussions at the CIBA Symposium of 1966. *Perspectives in Biology & Medicine, 58*(4), 379-394. doi:https://dx.doi.org/10.1353/pbm.2015.0030

Warner, J. H. (2004). Grand narrative and its discontents: medical history and the social transformation of American medicine. *Journal of Health Politics, Policy & Law, 29*(4/5), 757-780.

Watkins, A. (2011). Commentary on "going against the grain: Liam's story". *Journal of Paediatrics & Child Health, 47*(9), 659-660. doi:https://dx.doi.org/10.1111/j.1440-1754.2011.02173.x

Watkins, J., Stanton, L., Saunders, B., Lasocki, G., Chung, P., & Hibberd, P. (2011). Working in partnership with family carers: the importance of learning from carers' experiences. *Quality in Ageing & Older Adults, 12*(2), 103-108.

Wear, D., & Jones, T. (2010). Bless me reader for I have sinned: physicians and confessional writing. *Perspectives in Biology & Medicine, 53*(2), 215-230. doi:https://dx.doi.org/10.1353/pbm.0.0156

Weaver, R. (1990). Politics of Story-Telling: The Effects of Dramatic Structuring in Daily Current Affairs TV. *Media Information Australia*(57), 12-18.

Weeks, W. B. (2016). Hailey. *JAMA, 316*(19), 1975-1976. doi:https://dx.doi.org/10.1001/jama.2016.10251

Weiler-Ravell, D., Leventhal, A., Berlowitz, Y., Rishpon, S., & Chemtob, D. (2004). Circumstances leading to the formulation and implementation of a new TB control program in Israel: a case study in public health and policy. *Journal of Public Health Policy, 25*(1), 23-37. doi:https://dx.doi.org/10.1057/palgrave.jphp.3190002
Weinstock, M. (2007). In box: storyboard. Legislatures turn up heat on hospital-acquired infections. *H&HN: Hospitals & Health Networks, 81*(4), 24-24.
Weishaar, H., Collin, J., & Amos, A. (2016). Tobacco Control and Health Advocacy in the European Union: Understanding Effective Coalition-Building. *Nicotine & Tobacco Research, 18*(2), 122-129. doi:https://dx.doi.org/10.1093/ntr/ntv016
Weishaar, H., Dorfman, L., Freudenberg, N., Hawkins, B., Smith, K., Razum, O., & Hilton, S. (2016). Why media representations of corporations matter for public health policy: a scoping review. *BMC Public Health, 16*, 899. doi:https://dx.doi.org/10.1186/s12889-016-3594-8
Weiss, K. J., Watson, C., & Xuan, Y. (2014). Frye's Backstory: a tale of murder, a retracted confession, and scientific hubris. *Journal of the American Academy of Psychiatry & the Law, 42*(2), 226-233.
Wenger, R. R. (2015). Environmental Advocacy. The Voices of Sacred Stories. *Health Progress, 96*(2), 25-29.
Wexler, N. S. (2012). Huntington's disease: advocacy driving science. *Annual Review of Medicine, 63*, 1-22. doi:https://dx.doi.org/10.1146/annurev-med-050710-134457
Whelan, E., Asbridge, M., & Haydt, S. (2011). Representations of OxyContin in North American newspapers and medical journals. *Pain Research & Management, 16*(4), 252-258.
White, C. (2006). The spanner trials and the changing law on sadomasochism in the UK. *Journal of Homosexuality, 50*(2-3), 167-187. doi:https://dx.doi.org/10.1300/J082v50n02_08
White, M. L., & Fletcher, J. C. (1990). The story of Mr. and Mrs. Doe: "You can't tell my husband he's dying; it will kill him.". *Journal of Clinical Ethics, 1*(1), 59-62.
Whitehead, M., Petticrew, M., Graham, H., Macintyre, S. J., Bambra, C., & Egan, M. (2004). Evidence for public health policy on inequalities: 2: assembling the evidence jigsaw. *Journal of Epidemiology & Community Health, 58*(10), 817-821. doi:https://dx.doi.org/10.1136/jech.2003.015297
Whitworth, A. (2016). Neoliberal paternalism and paradoxical subjects: Confusion and contradiction in UK activation policy. *Critical Social Policy, 36*(3), 412-431. doi:10.1177/0261018315624442
Wiethoff, C. (2002). Naming, blaming, and claiming in public disputes: the 1998 Maine referendum on civil rights protection for gay men and lesbians. *Journal of Homosexuality, 44*(1), 61-82.
Williams, B., Powell, A., Hoskins, G., & Neville, R. (2008). Exploring and explaining low participation in physical activity among children and young people with asthma: a review. *BMC Family Practice, 9*, 40. doi:https://dx.doi.org/10.1186/1471-2296-9-40
Willis, D. J., DeLeon, P. H., Haldane, S., & Heldring, M. B. (2014). A Policy Article--Personal Perspectives on the Public Policy Process: Making a Difference. *Professional Psychology: Research & Practice, 45*(2), 143-151. doi:10.1037/a0036234
Wilson-Clay, B., Rourke, J. W., Bolduc, M. B., Stagg, J. D., Flatau, G., & Vaugh, B. (2005). Learning to lobby for probreastfeeding legislation: the story of a Texas bill to create a breastfeeding-friendly physician designation. *Journal of Human Lactation, 21*(2), 191-198. doi:https://dx.doi.org/10.1177/0890334405275372
Wilson, M., & Johnson, L. H. (2003). How you can make a difference in the political process. *PT: Magazine of Physical Therapy, 11*(4), 44-76.
Winakur, J. (2010). The power of story in long-term care. *Caring for the Ages, 11*(1), 18-18.
Winchester, M. S. (2016). Marriage, violence and HIV: the shifting policy context in Uganda. *Culture, Health & Sexuality*, 1-14. doi:https://dx.doi.org/10.1080/13691058.2016.1184313
Winett, L. B., & Lawrence, R. G. (2005). The Rest of the Story: Public Health, the News, and the 2001 Anthrax Attacks. *Harvard International Journal of Press/Politics, 10*(3), 3-25. doi:10.1177/1081180X05279532

Witson, J., Fan, D., Harwood, E., & Wagenaar, A. (2004). Media advocacy and underage drinking policies: A study of Louisiana print media from 1994 through 2003. *Conference Papers -- International Communication Association*, 1.
Wittenstein, S. H. (2013). The Manliest Man: Samuel G. Howe and the Contours of Nineteenth-Century American Reform. *Journal of Visual Impairment & Blindness, 107*(4), 317-320.
Woeppel, S. (2013). Stories of a Medical Writing Expatriate: My Transition into Regulatory Affairs. *AMWA Journal: American Medical Writers Association Journal, 28*(2), 72-73.
Wohlberg, S. H. (2013). My life with RE. *Epilepsia, 54*(12), 2023-2024. doi:https://dx.doi.org/10.1111/epi.12467
Wohlers, A. E. (2013). Labeling of genetically modified food: closer to reality in the United States? *Politics & the Life Sciences, 32*(1), 73-84. doi:https://dx.doi.org/10.2990/32_1_73
Woldeguiorguis, I. M. (2003). Racism and sexism in child welfare: effects on women of color as mothers and practitioners. *Child Welfare, 82*(2), 273-288.
Wolfe-Dawson, L. (2009). *A biographical study of namesake John F. Tinker on the landmark legal case Tinker et al. v.The Des Moines Independent Community School District et al.* (70), ProQuest Information & Learning, US. Retrieved from http://search.ebscohost.com/login.aspx?direct=true&db=psyh&AN=2009-99150-170&site=ehost-live Available from EBSCOhost psyh database.
Wollaston, S. (2013). Sarah Wollaston: from GP to MP. Interview by Krishna Chinthapalli. *BMJ, 347*, f6905. doi:https://dx.doi.org/10.1136/bmj.f6905
Wood, A. (2003). Come Visit With Sister Sally, Hawk and Spenser; Hone Your Occam's Razor and Your Ukulele. *Communication World, 20*(5), 46.
Wood, J. F. (2005). Living by Parental Narratives: A Narrative Criticism of Marian Wright Edelman's The Measure of Our Success: A Letter to My Children and Yours. *Texas Speech Communication Journal, 29*(2), 106-117.
Wood, W. (2007). Associate editor's note—The sustaining power of ideas. *American Journal of Occupational Therapy, 61*(5), 597-598. doi:10.5014/ajot.61.5.597
Worthy, S. L. (2016). Don't sell out safety: a call to preserve risk evaluation and mitigation strategies to reduce harm to patients and the public in the U.S. *Journal of Pharmaceutical Policy & Practice, 9*, 2. doi:https://dx.doi.org/10.1186/s40545-016-0051-0
Wright, M. (2015). Is there value in the Relative Value Study? Caution before Australian Medicare reform. *Medical Journal of Australia, 203*(8), 331-333.
Wright, N., Bartlett, P., & Callaghan, P. (2008). A review of the literature on the historical development of community mental health services in the United Kingdom. *Journal of Psychiatric & Mental Health Nursing, 15*(3), 229-237. doi:https://dx.doi.org/10.1111/j.1365-2850.2007.01218.x
Wye, L., Brangan, E., Cameron, A., Gabbay, J., Klein, J. H., & Pope, C. (2015). Evidence based policy making and the 'art' of commissioning - how English healthcare commissioners access and use information and academic research in 'real life' decision-making: an empirical qualitative study. *BMC Health Services Research, 15*, 430. doi:https://dx.doi.org/10.1186/s12913-015-1091-x
Wylie, K., & Nebauer, M. (2011). The fragmented story of pain: a saga of economic discourse, confusion and lack of holistic assessment in the residential care of older people. *Collegian: Journal of the Royal College of Nursing, Australia, 18*(1), 11-18.
Wynia, M. K. (2007). Public health, public trust and lobbying. *American Journal of Bioethics, 7*(6), 4-7. doi:https://dx.doi.org/10.1080/15265160701429599
Wynn, M. (2010). Ellen's Hand. *Violence Against Women, 16*(9), 1055-1060. doi:https://dx.doi.org/10.1177/1077801210379377
Yankah, K. (1992). Traditional lore in population communication: the case of the Akan in Ghana. *Africa Media Review, 6*(1), 15-24.

Yanovitzky, I., & Stryker, J. (2001). Mass media, social norms, and health promotion efforts: A longitudinal study of media effects on youth binge drinking. *Communication Research, 28*(2), 208-239. doi:10.1177/009365001028002004

Yapp, K. E., Hopcraft, M. S., & Parashos, P. (2011). Articaine: a review of the literature. *British Dental Journal, 210*(7), 323-329. doi:https://dx.doi.org/10.1038/sj.bdj.2011.240

Yarborough, M. (2005). Deciding for others at the end of life: storytelling and moral agency. *Journal of Clinical Ethics, 16*(2), 127-143.

Yaris, F., Dikici, M., Akbulut, T., Yaris, E., & Sabuncu, H. (2004). Story of benzene and leukemia: epidemiologic approach of Muzaffer Aksoy. *Journal of Occupational Health, 46*(3), 244-247.

Yeboah, T. (2000). Improving the provision of traditional health knowledge for rural communities in Ghana. *Health Libraries Review, 17*(4), 203-208.

Ying, Z. H. U. (2016). Corruption and its (dis)content: the rise and fall of Chinese officialdom television dramas (Vol. 57, pp. 235-249).

Yoon, Y., & Lee, S. (2008). Frame Building of Prescription Drug Imports From Canada: An Analysis of Policy Actors' Message Frames in News Stories. *Conference Papers -- International Communication Association*, 1-26.

Yoshikawa, H., & Olazagasti, M. A. R. (2011). The neglected role of community narratives in culturally anchored prevention and public policy. In M. S. Aber, K. I. Maton, E. Seidman, M. S. Aber, K. I. Maton, & E. Seidman (Eds.), *Empowering settings and voices for social change.* (pp. 173-192). New York, NY, US: Oxford University Press.

Zebregs, S., van den Putte, B., de Graaf, A., Lammers, J., & Neijens, P. (2015). The effects of narrative versus non-narrative information in school health education about alcohol drinking for low educated adolescents. *BMC Public Health, 15*, 1085. doi:https://dx.doi.org/10.1186/s12889-015-2425-7

Zwald, M., Jernigan, J., Payne, G., & Farris, R. (2013). Developing stories from the field to highlight policy, systems, and environmental approaches in obesity prevention. *Preventing Chronic Disease, 10*, 120141. doi:https://dx.doi.org/10.5888/pcd10.120141

Zwelling, E. (2001). The history of lamaze continues: an interview with sunnye strickland. *Journal of Perinatal Education, 10*(1), 13-20. doi:https://dx.doi.org/10.1624/105812401X88011

Zwelling, E. (2002). Activist for change: an interview with suzanne arms. *Journal of Perinatal Education, 11*(4), 11-24. doi:https://dx.doi.org/10.1624/105812402X88920
