# Supplementary material for: Using narratives to impact health policy-making: a systematic review
Source: Health Res Policy Syst. 2019 Mar 5;17:26. doi: 10.1186/s12961-019-0423-4 (PMC6402129; doi:10.1186/s12961-019-0423-4)
Supplement: Supplementary file 3 — Assessment of the reporting of included case studies. (PDF 338 kb) [file 12961_2019_423_MOESM3_ESM.pdf]

Additional file 3: Assessment of the reporting of included case studies

Items	Johnson 2014[1]	Shi, 2013[2]	Krueger 2007[3]	Wilcock 2003[4]	Fitzgerald 2013[5]	Umuhoza, 2013[6]	Leith, 2006[7]	Neuhausen 2013[8]	Slaton 2012[9]	Sharf, 2001[10]	Trossman, 1999[11]	MacKenzie, 2008[12]	*MacGregor, 2011[13]	*Marcus, 2010[14]	Rosenbaum, 2016[15]
**Describing the design**															
Define the research as a case study	yes	no	no	yes	yes	no	yes	no	no	no	no	yes	no	no	no
State the broad aims of the study	yes	yes	yes	yes	yes	yes	yes	yes	yes	yes	yes	yes	yes	yes	no
State the research question(s)/hypotheses	yes	yes	yes	yes	yes	no	no	no	yes	no	no	yes	yes	no	no
Identify the specific case(s) and justify the selection	no	yes	no	no	no	no	no	yes	yes	no	no	yes	yes	yes	yes
**Describing the data collection**															
Describe how data were collected	Yes	no	no	yes	yes	no	no	no	no	no	no	yes	no	no	no
Describe the sources of evidence used	yes	yes	Yes	yes	yes	yes	no	no	no	yes	no	yes	yes	yes	no
Describe any ethical considerations and obtainment of relevant approvals, access and permissions	no	no	no	yes	no	no	no	no	no	no	no	no	no	no	no
**Describing the data analysis**															
Describe the analysis methods	no	no	no	yes	yes	no	no	no	no	no	no	yes	no	no	no
**Interpreting the results**															
Describe any inherent	yes	no	no	no	yes	no	no	no	no	no	no	no	no	no	no

shortcomings in the design and analysis and how these might have influenced the findings															
Consider the appropriateness of methods used for the question and subject matter and why it was that qualitative methods were appropriate	yes	no	no	yes	yes	no	yes	no	yes	no	no	no	no	no	no
Discuss the data analysis	yes	no	no	yes	yes	no	no	no	no	no	no	yes	no	no	no
Ensure that the assertions are sound, neither over- nor under-interpreting the data	yes	no	no	yes	yes	yes	yes	no	yes	yes	no	yes	no	no	yes
State any caveats about the study	yes	no	no	no	yes	no	no	no	no	no	no	no	no	no	no

*Assessment was done only for the section related to impact of narratives on health policymaking (which is relevant to our question)

1. Johnson K, Minogue, G., & Hopklins, R. Inclusive research: Making a difference to policy and legislation. . *Journal of Applied Research in Intellectual Disabilities* 2014;27(1):76-84.
2. Shi L. Micro-blogs, online forums, and the birth-control policy: social media and the politics of reproduction in China. *Cult Med Psychiatry* 2014;38(1):115-32. doi: 10.1007/s11013-013-9351-x
3. Krueger G. "For Jimmy and the boys and girls of America": publicizing childhood cancers in twentieth-century America. *Bull Hist Med* 2007;81(1):70-93. doi: 10.1353/bhm.2007.0004
4. Wilcock PM, Brown GC, Bateson J, et al. Using patient stories to inspire quality improvement within the NHS Modernization Agency collaborative programmes. *J Clin Nurs* 2003;12(3):422-30.
5. Fitzgerald JL. Supervised injecting facilities: A case study of contrasting narratives in a contested health policy arena. *Critical Public Health* 2013;23(1): 77-94.
6. Umuhoza C, Oosters B, van Reeuwijk M, et al. Advocating for safe abortion in Rwanda: how young people and the personal stories of young women in prison brought about change. *Reprod Health Matters* 2013;21(41):49-56. doi: 10.1016/S0968-8080(13)41690-7
7. Leith KH, & Phillips, L. . In Their Own Voices" Using Qualitative Research and Consumer Narratives for Systems Change. . *The Social Policy Journal* 2006;4(3-4): 19-35.
8. Neuhausen K. Awakening advocacy: how students helped save a safety-net hospital in Georgia. *Health Aff (Millwood)* 2013;32(6):1161-4. doi: 10.1377/hlthaff.2012.0662
9. Slaton AE, Cecil CW, Lambert LE, et al. What a difference family-driven makes: stories of success and lessons learned. *Am J Community Psychol* 2012;49(3-4):538-45. doi: 10.1007/s10464-011-9449-7
10. Sharf BF. Out of the closet and into the legislature: breast cancer stories. *Health Aff (Millwood)* 2001;20(1):213-8.
11. Trossman S. MNA president uses personal story to push for needlestick legislation. *Am Nurse* 1999;31(3):13.
12. MacKenzie R, Imison M, Chapman S, et al. Mixed messages and a missed opportunity: Australian news media coverage of Clare Oliver's campaign against solaria. *Med J Aust* 2008;189(7):371-4.
13. Macgregor H, Mills E. Framing rights and responsibilities: accounts of women with a history of AIDS activism. *BMC Int Health Hum Rights* 2011;11 Suppl 3:S7. doi: 10.1186/1472-698X-11-S3-S7
14. Marcus PM, Huang GC, Beck V, et al. The impact of a primetime cancer storyline: from individual knowledge and behavioral intentions to policy-level changes. *J Cancer Educ* 2010;25(4):484-9. doi: 10.1007/s13187-010-0093-y
15. Rosenbaum L. N-of-1 Policymaking--Tragedy, Trade-offs, and the Demise of Morcellation. *N Engl J Med* 2016;374(10):986-90. doi: 10.1056/NEJMms1516161
